# Supplementary material for: Design, Synthesis, and Biological Evaluation of Phenyloxadiazole Sulfoxide Derivatives as Potent Pseudomonas aeruginosa Biofilm Inhibitors
Source: Molecules. 2023 May 4;28(9):3879. doi: 10.3390/molecules28093879 (PMC10180516; doi:10.3390/molecules28093879)
Supplement: Supplementary file 1 [file molecules-28-03879-s001.zip › molecules-2351158-supplementary.pdf]

Supporting Information

Article

Design, Synthesis, and Biological Evaluation of Phenyloxadiazole Sulfoxide Derivatives as Potent *Pseudomonas aeruginosa* Biofilm Inhibitors

Xinyi Ye *,†, Shen Mao †, Yasheng Li ‡, Zhikun Yang, Aoqi Du and Hong Wang *

College of Pharmaceutical Science & Collaborative Innovation Center of Yangtze River Delta Region Green
Pharmaceuticals, Key Laboratory of Marine Fishery Resources Exploitment & Utilization of Zhejiang
Province, Zhejiang University of Technology, 18 Chaowang Road, Hangzhou 310014, China;
1111923009@zjut.edu.cn (S.M.); liyasheng@ahmu.edu.cn (Y.L.); yangzk@zjut.edu.cn (Z.Y.);
2112007103@zjut.edu.cn (A.D.)

* Correspondence: xinyiye1020@zjut.edu.cn (X.Y.); hongw@zjut.edu.cn (H.W.); Tel.: +86-0571-8832-0622 (H.W.)

† These authors contributed equally to this work.

‡ Current Address: Department of Infectious Diseases & Anhui Center for Surveillance of Bacterial
Resistance, The First Affiliated Hospital of Anhui Medical University, Hefei 230022, China.

Pages S2-S54:

Figures S1-S53 ^{1}H NMR and ^{13}C NMR spectra of title target compounds **4a-4o**, **5a-5o**, **6a-6o**, **7a-7o**.

Pages S55-S72:

Figures S54-S105 HRMS spectrum of the compounds **4a-4o**, **5a-5o**, **6a-6o**, **7a-7o**.

Page S72-S76:

Figures S106-S113 IR (KBr) spectrum of the compound **5a**, **5b**, **5c**, **5f**, **5g**, **5i**, **5j**, **5k**.

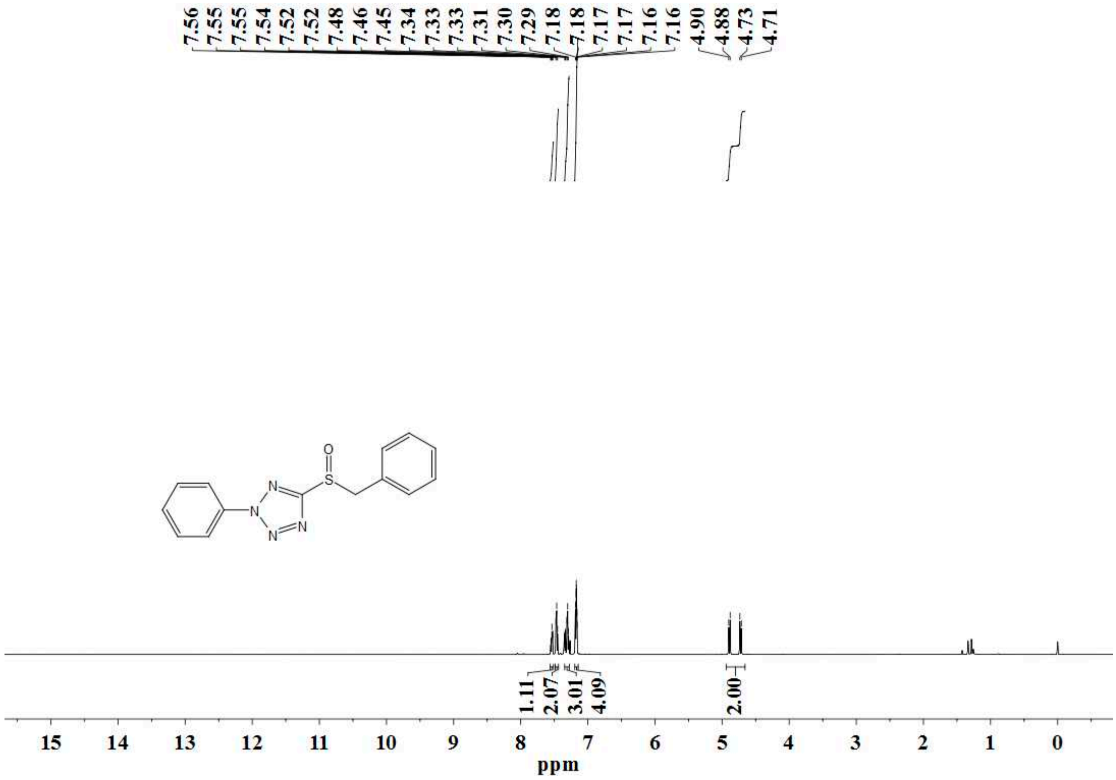

Figure S1-1. ^{1}H NMR spectrum of compound **4a**.

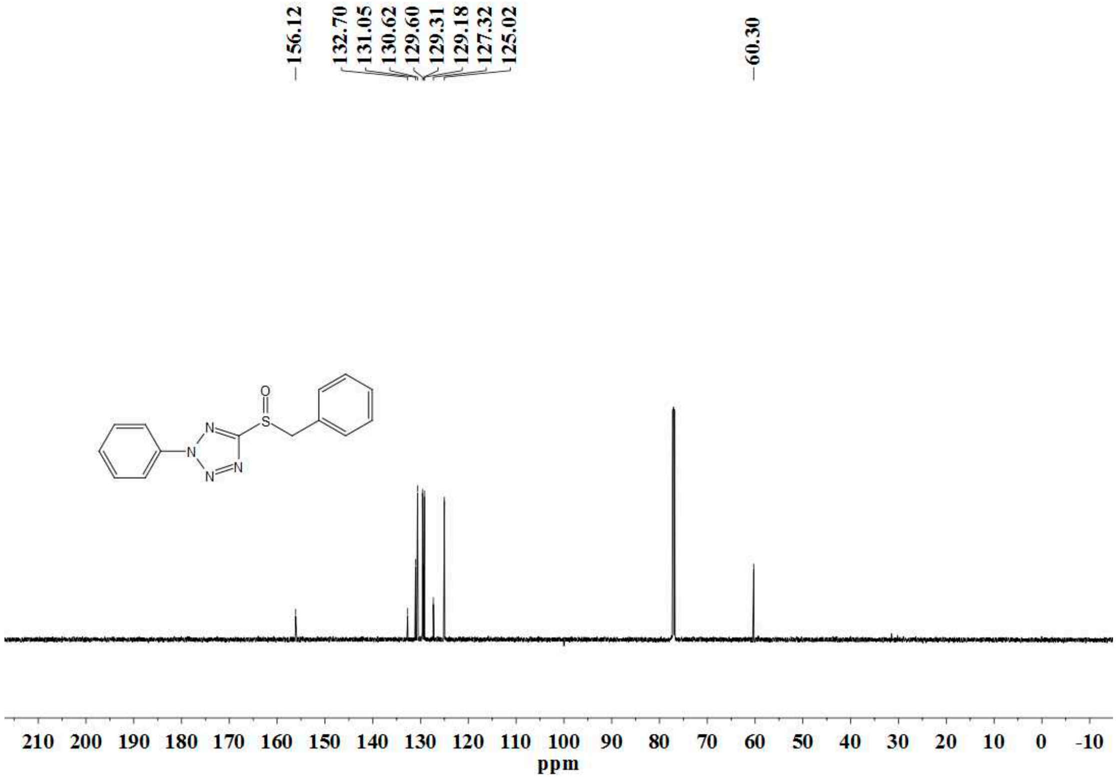

Figure S1-2. ^{13}C NMR spectrum of compound **4a**.

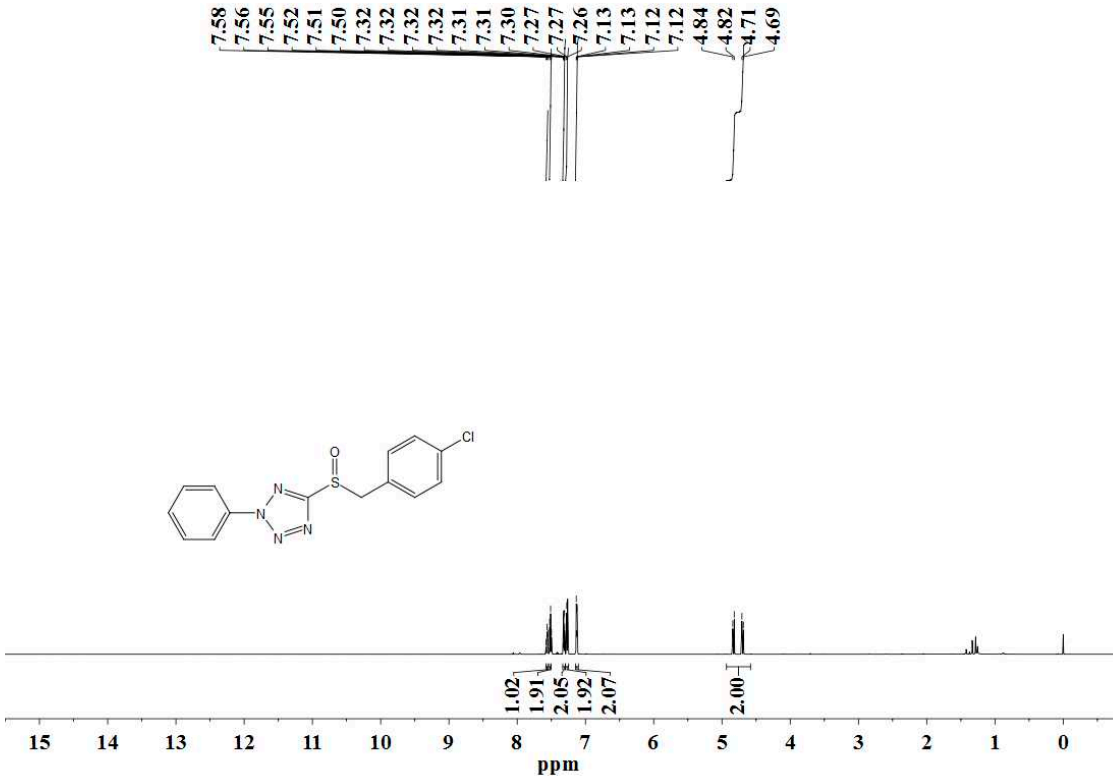

Figure S2-1. ^{1}H NMR spectrum of compound **4b**.

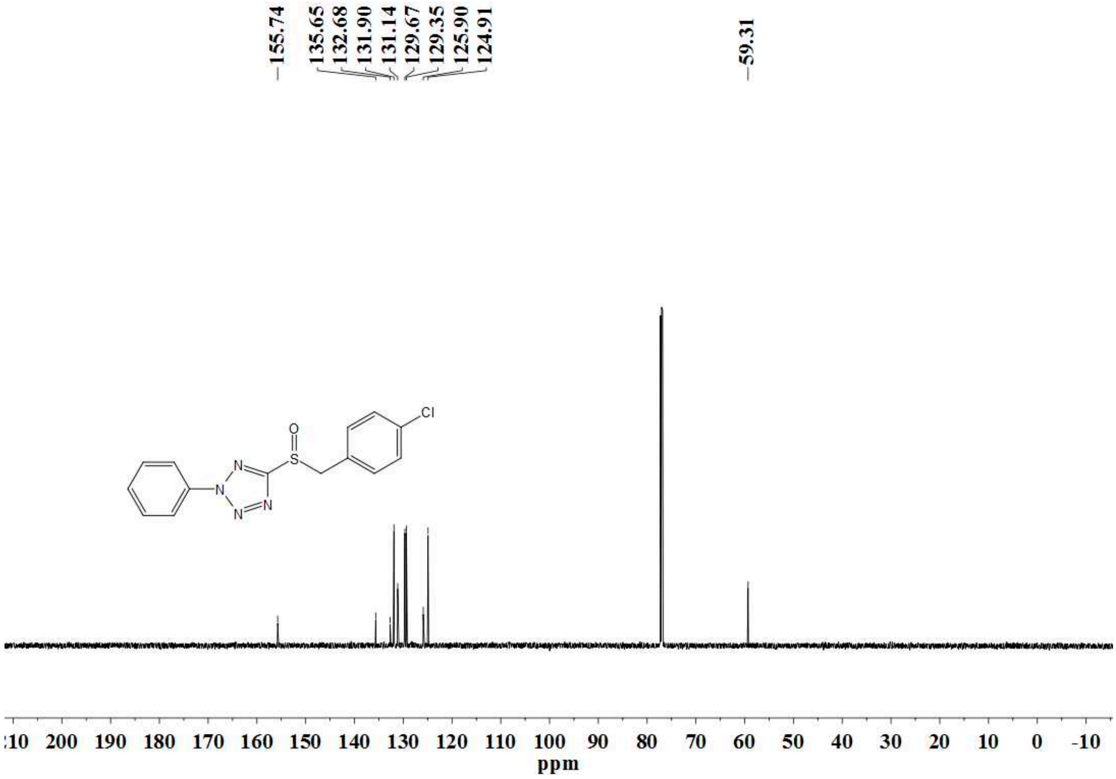

Figure S2-2. ^{13}C NMR spectrum of compound **4b**.

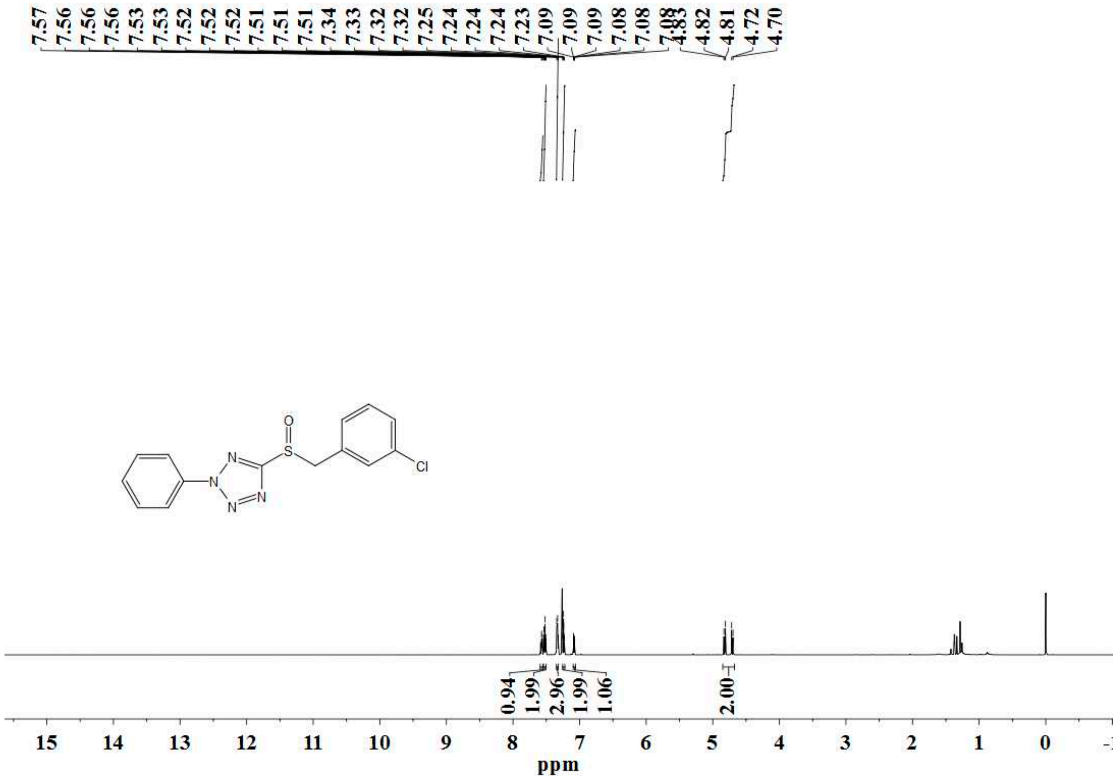

Figure S3-1. ^{1}H NMR spectrum of compound **4c**.

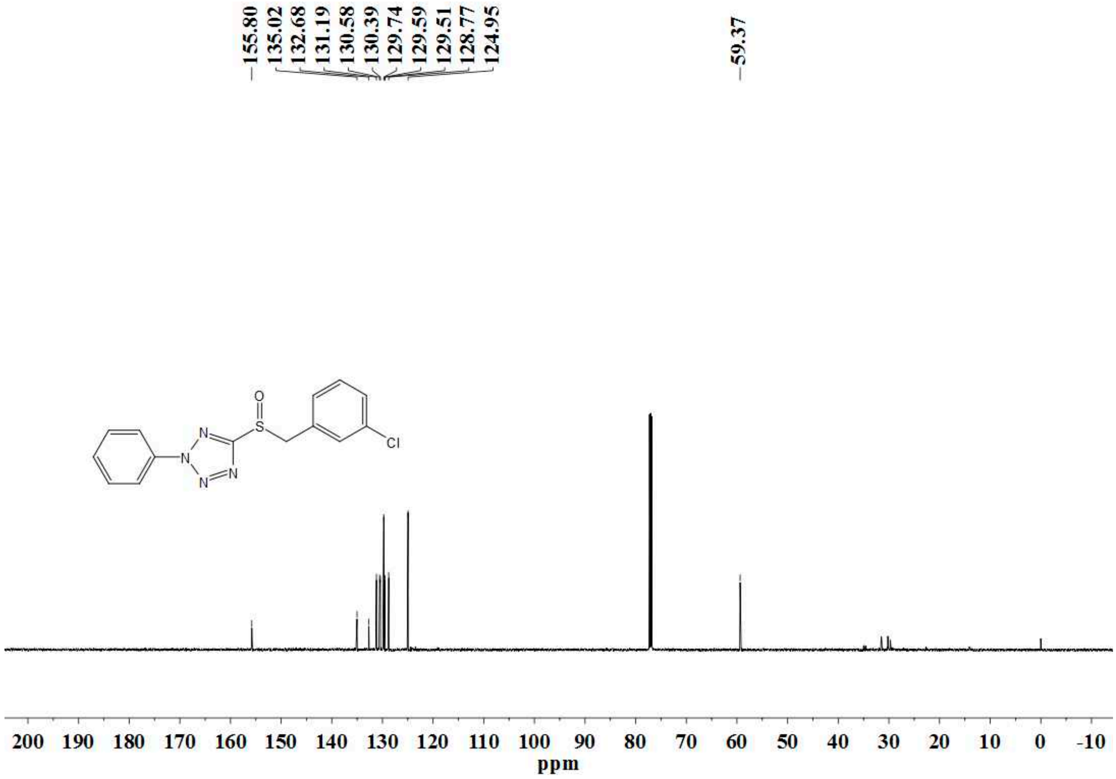

Figure S3-2. ^{13}C NMR spectrum of compound **4c**.

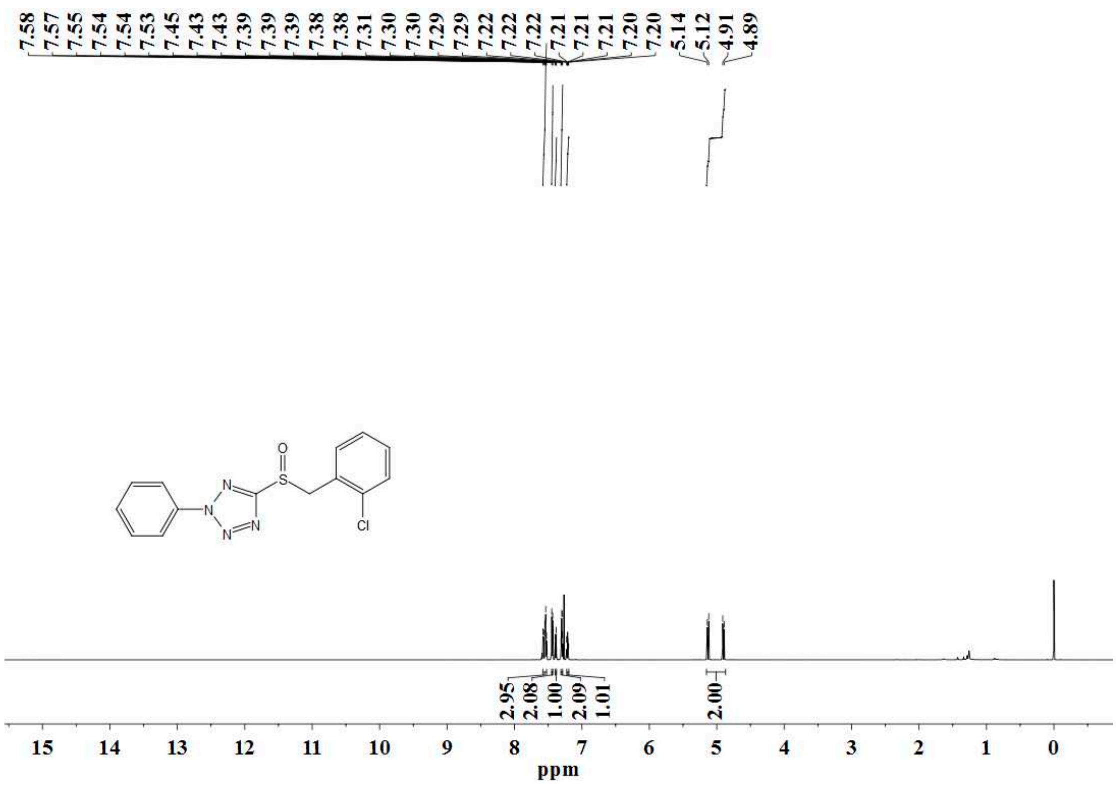

Figure S4-1. ^{1}H NMR spectrum of compound **4d**.

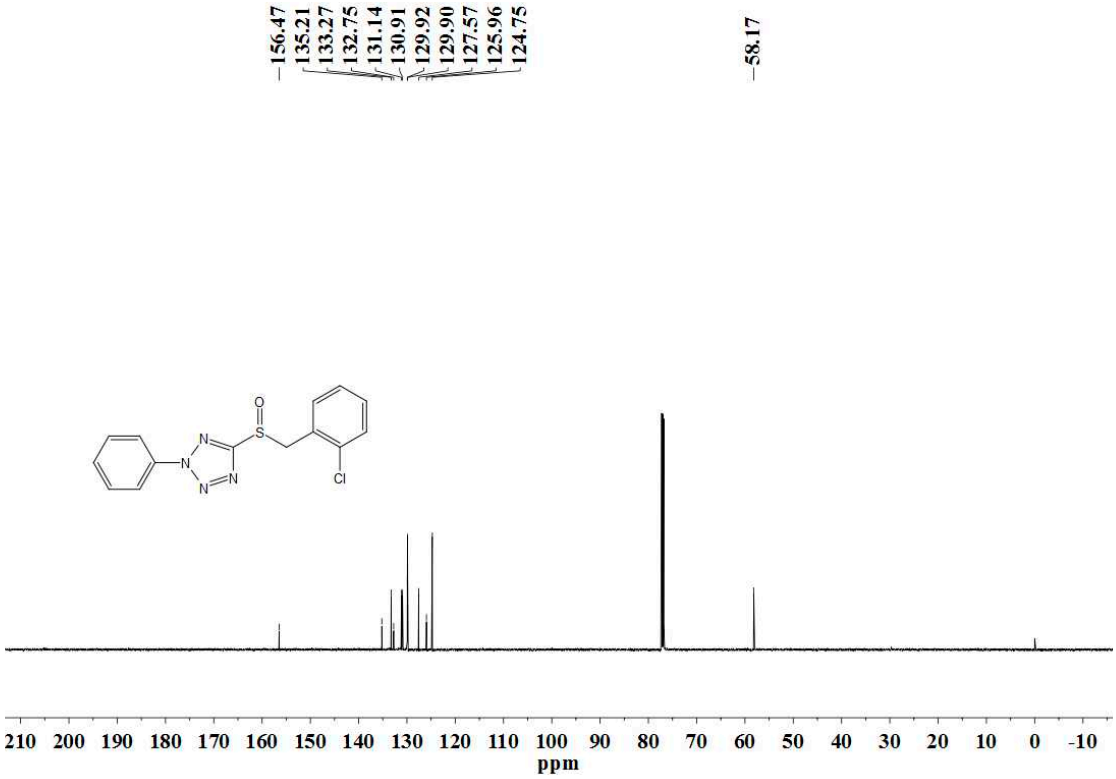

Figure S4-2. ^{13}C NMR spectrum of compound **4d**.

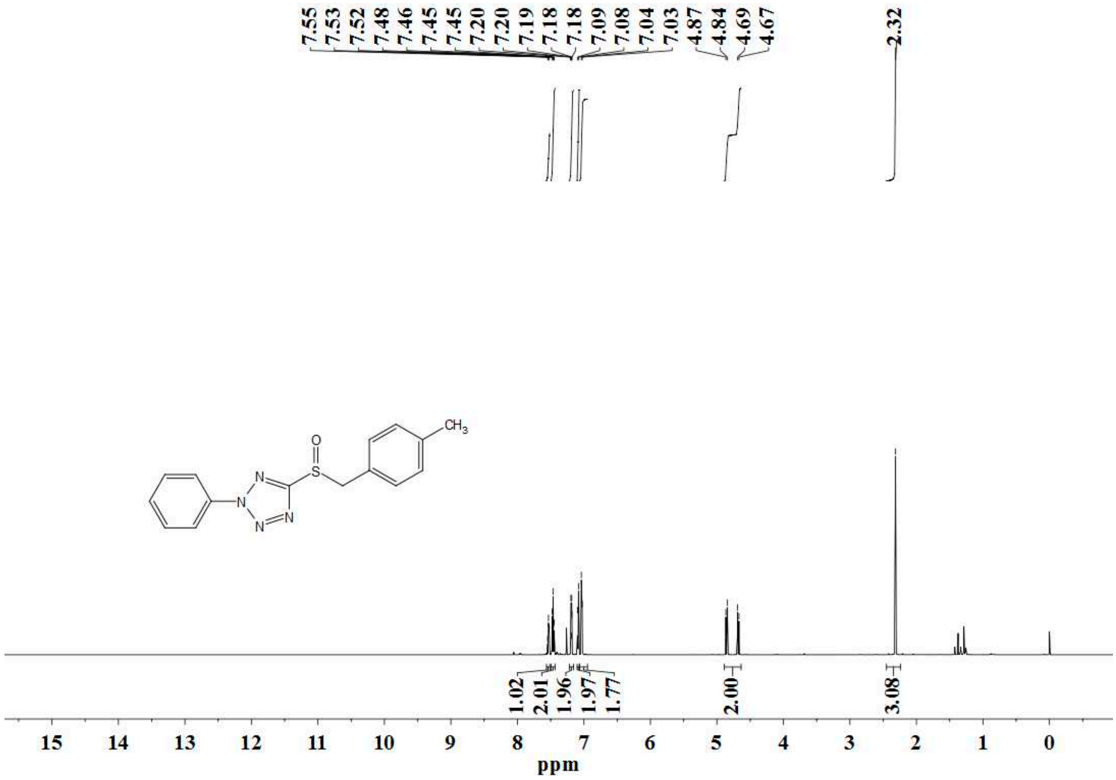

Figure S5-1. ^{1}H NMR spectrum of compound **4e**.

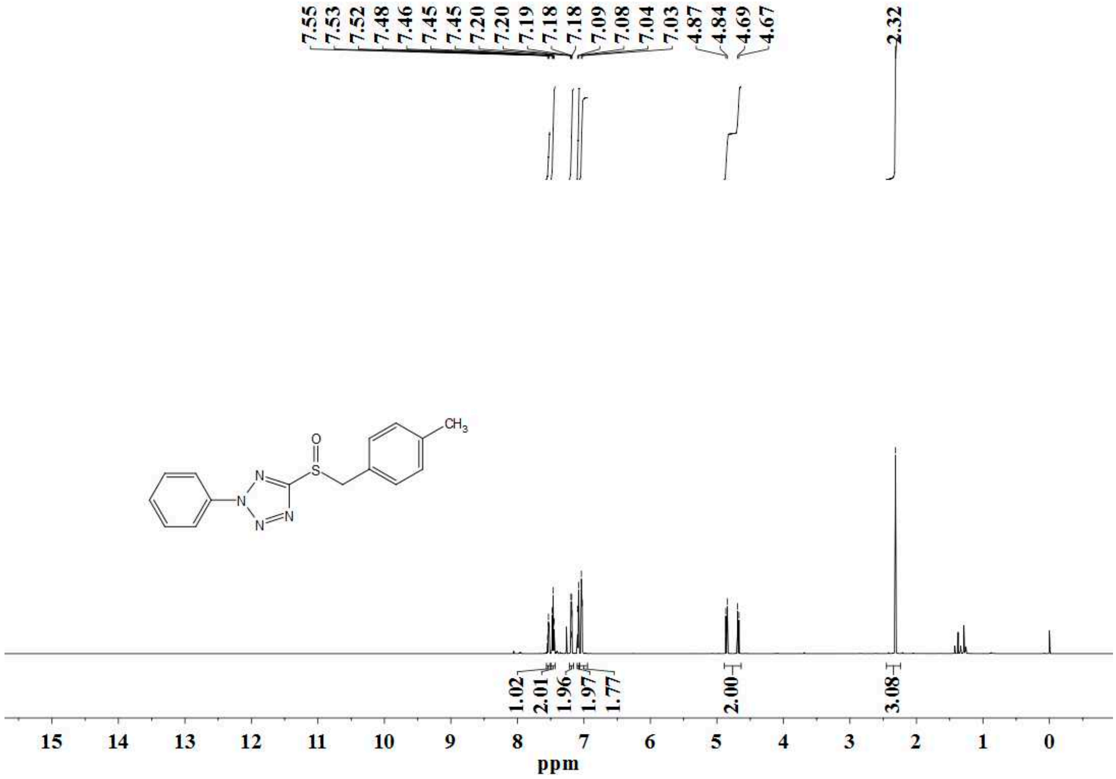

Figure S5-2. ^{13}C NMR spectrum of compound **4e**.

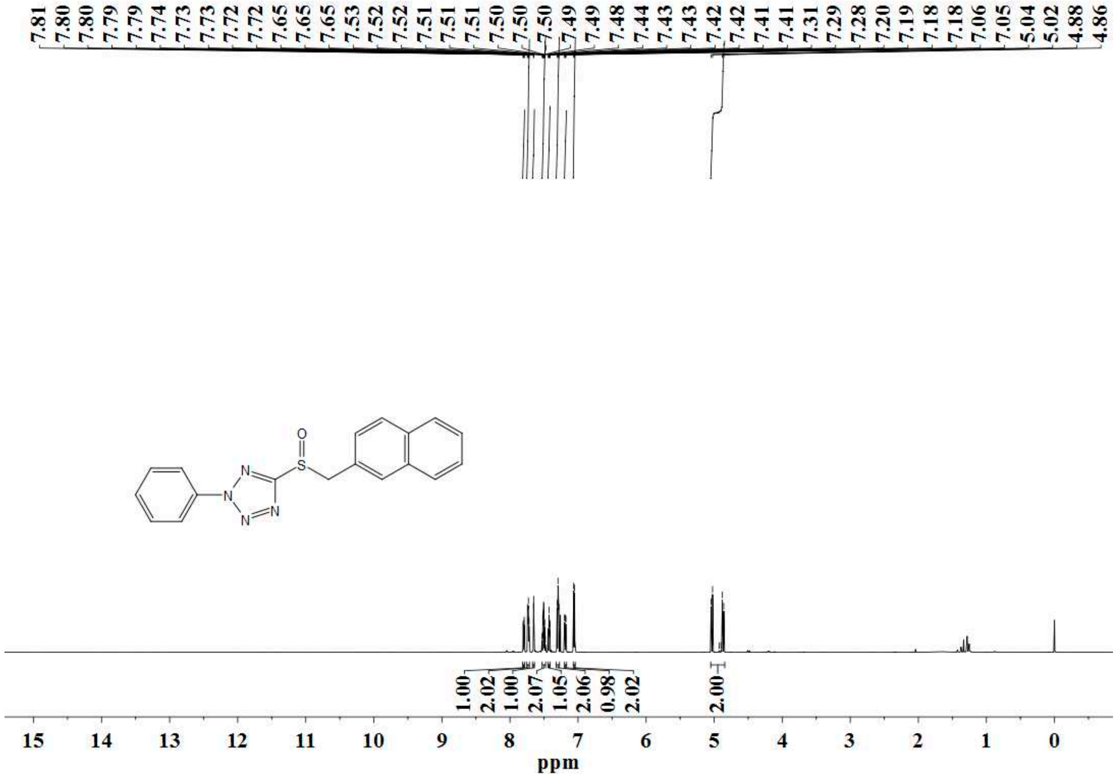

Figure S6-1. ^{1}H NMR spectrum of compound **4f**.

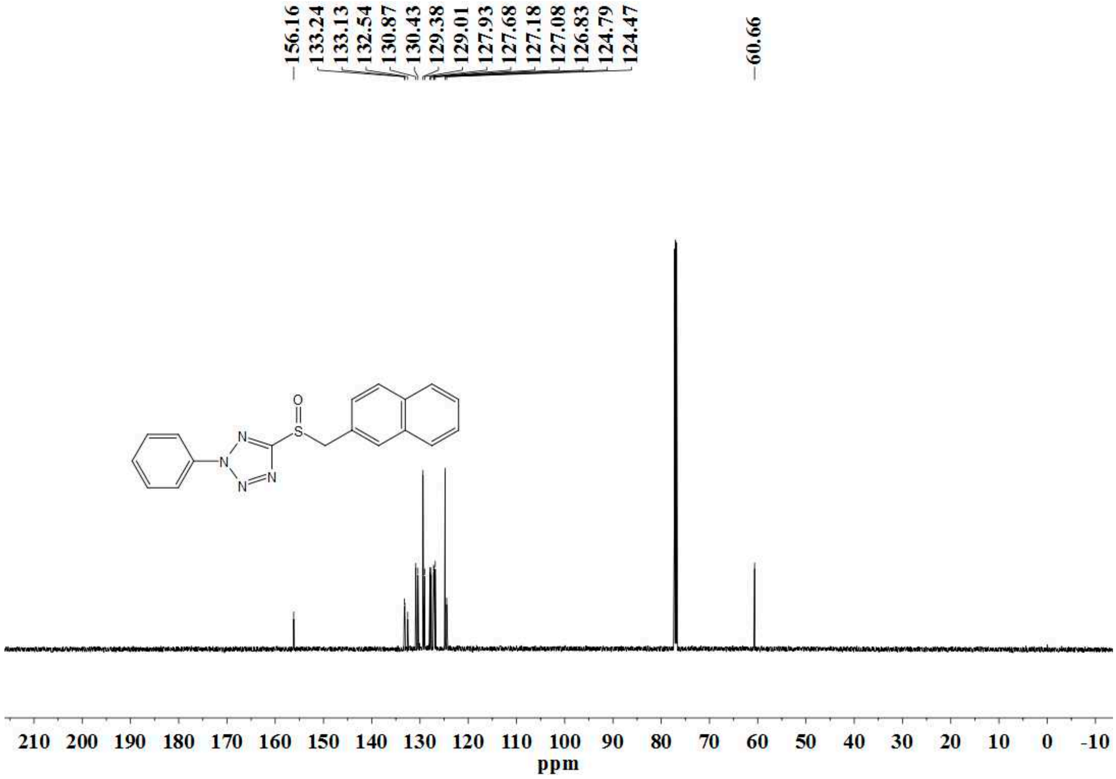

Figure S6-2. ^{13}C NMR spectrum of compound **4f**.

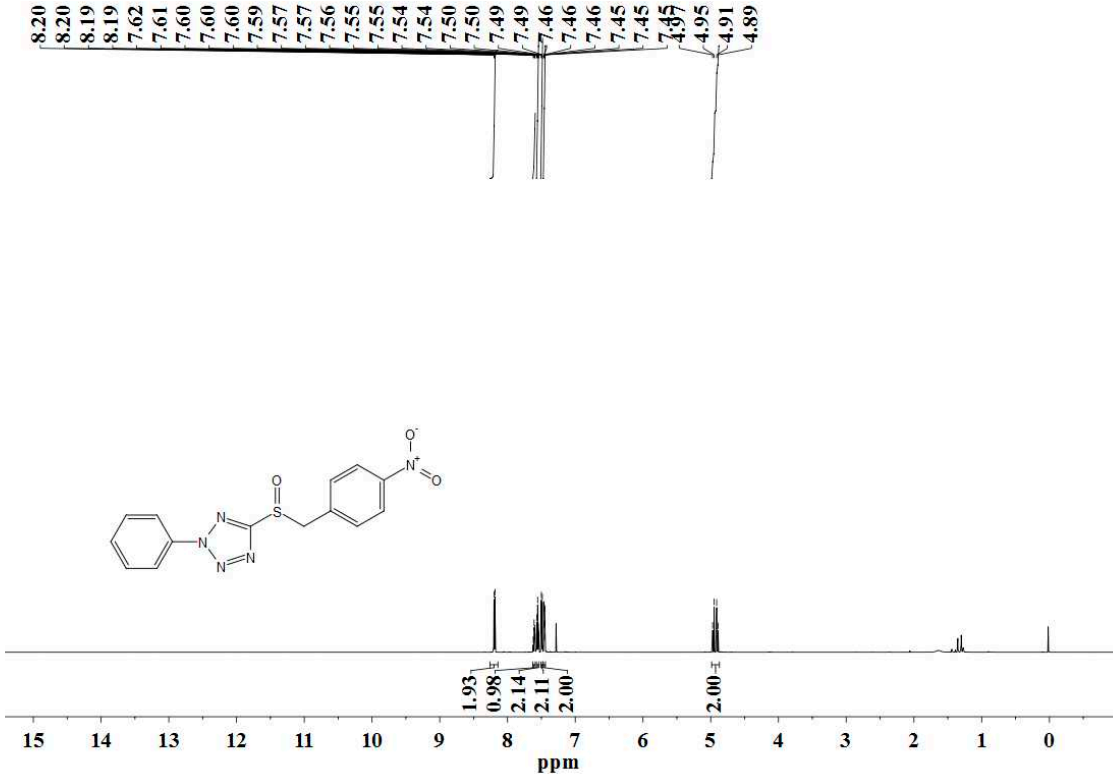

Figure S7-1. ^{1}H NMR spectrum of compound **4g**.

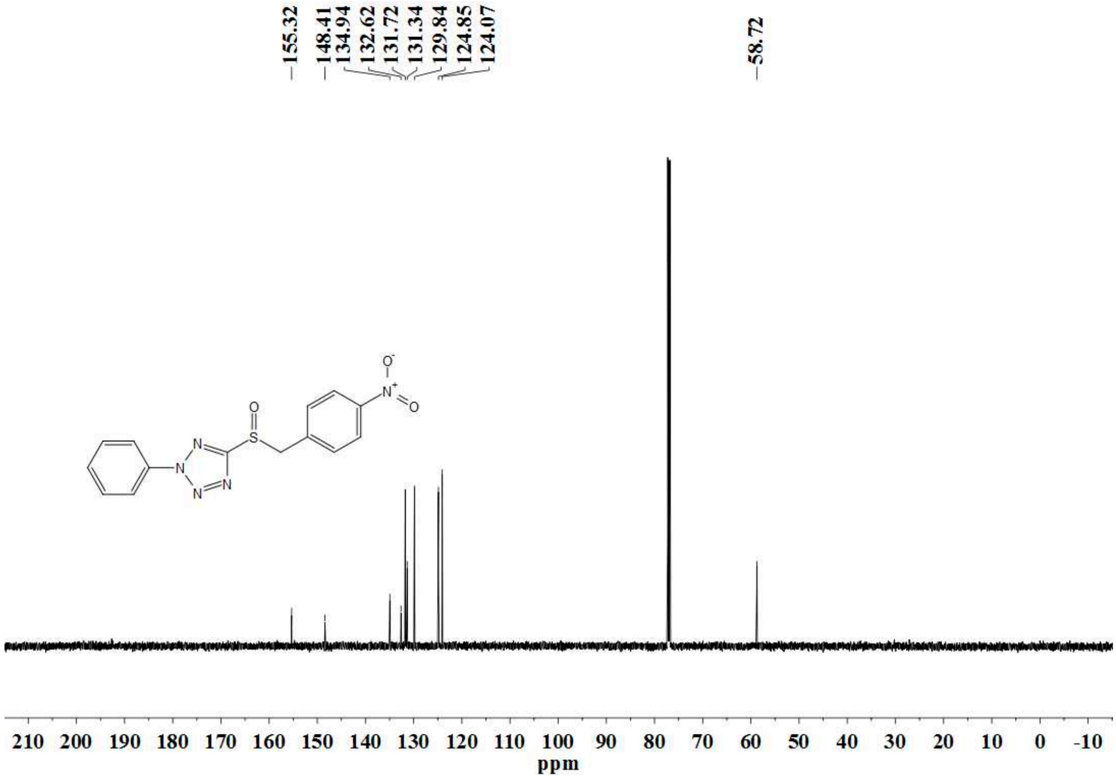

Figure S7-2. ^{13}C NMR spectrum of compound **4g**.

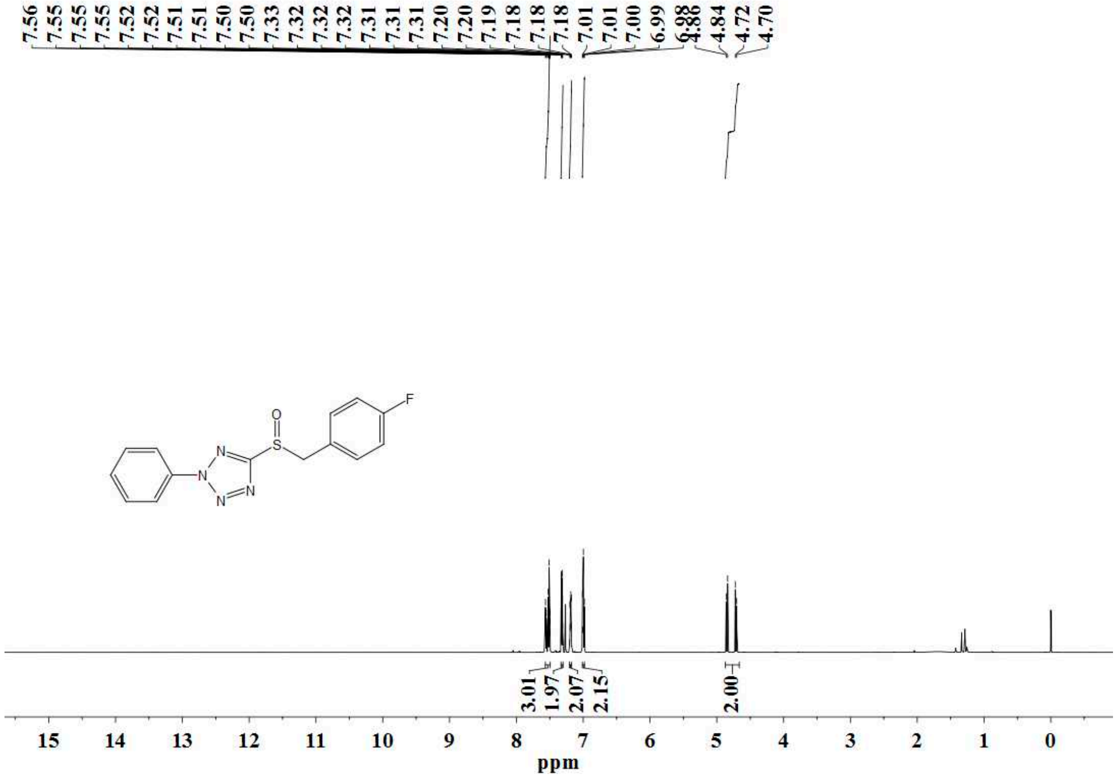

Figure S8-1. ^{1}H NMR spectrum of compound **4h**.

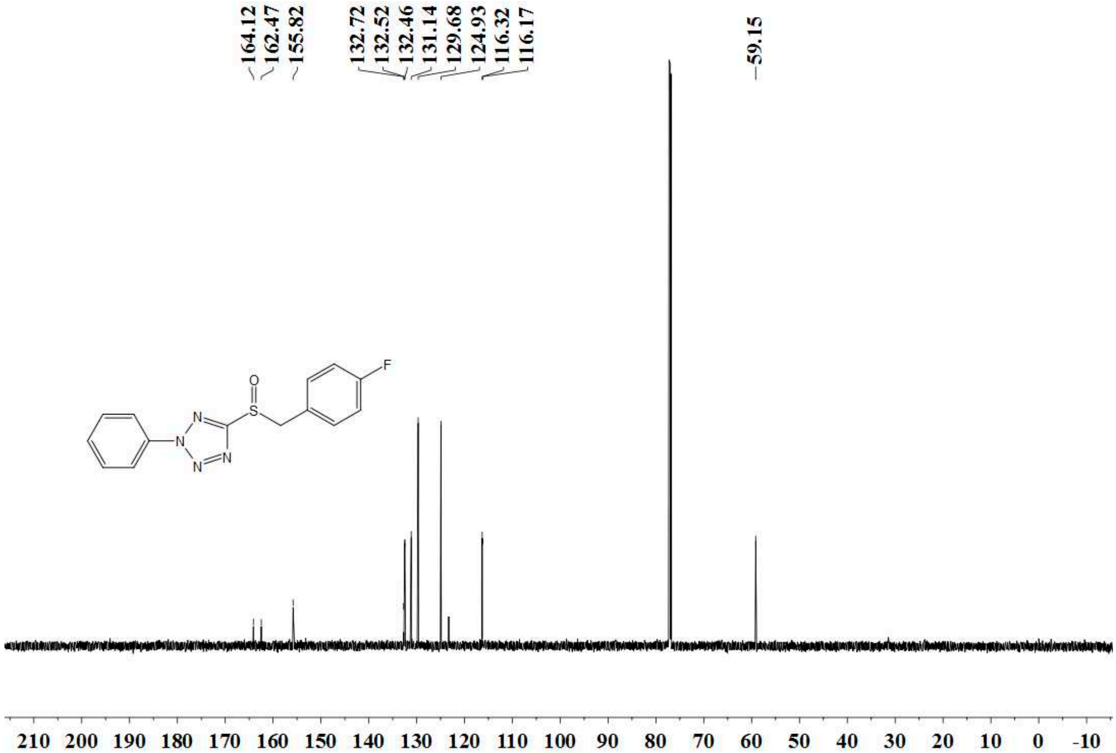

Figure S8-2. ^{13}C NMR spectrum of compound **4h**.

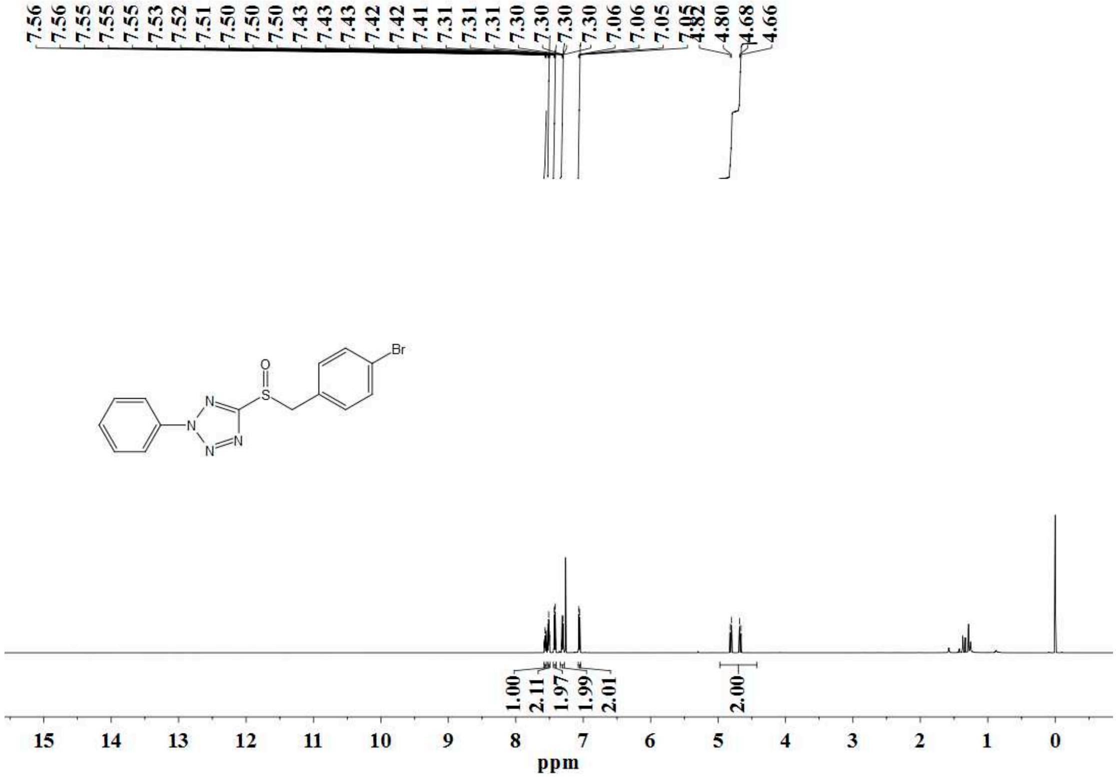

Figure S9-1. ^{1}H NMR spectrum of compound **4i**.

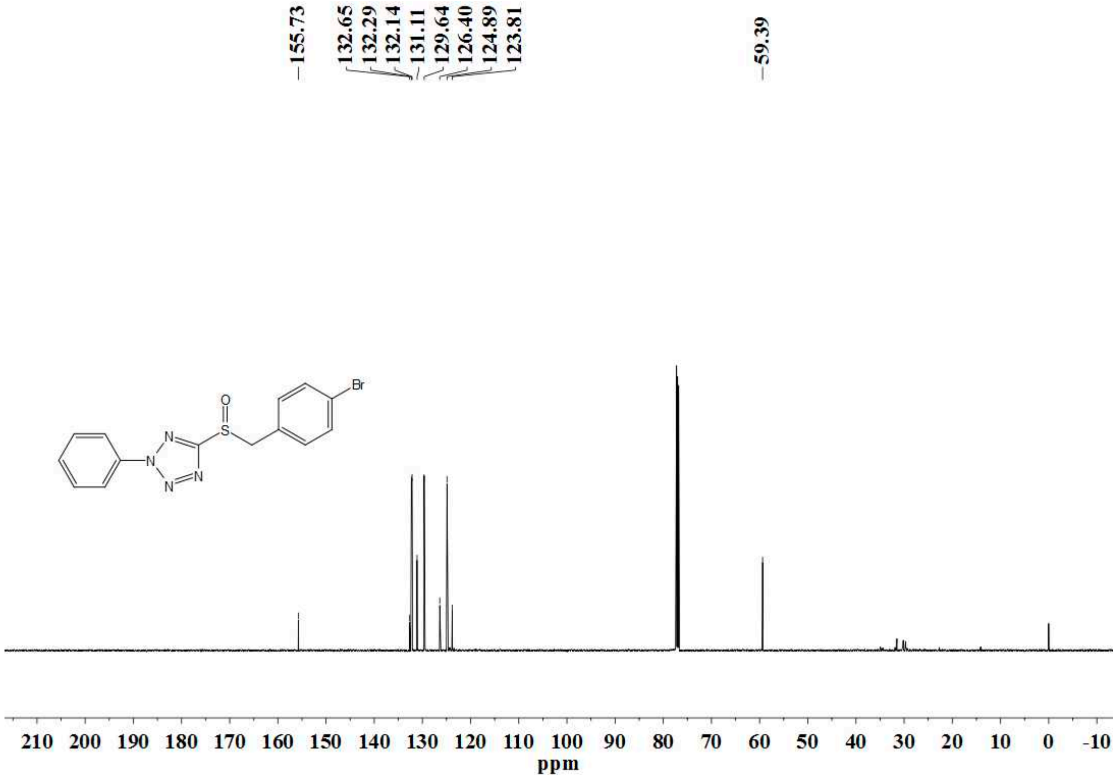

Figure S9-2. ^{13}C NMR spectrum of compound **4i**.

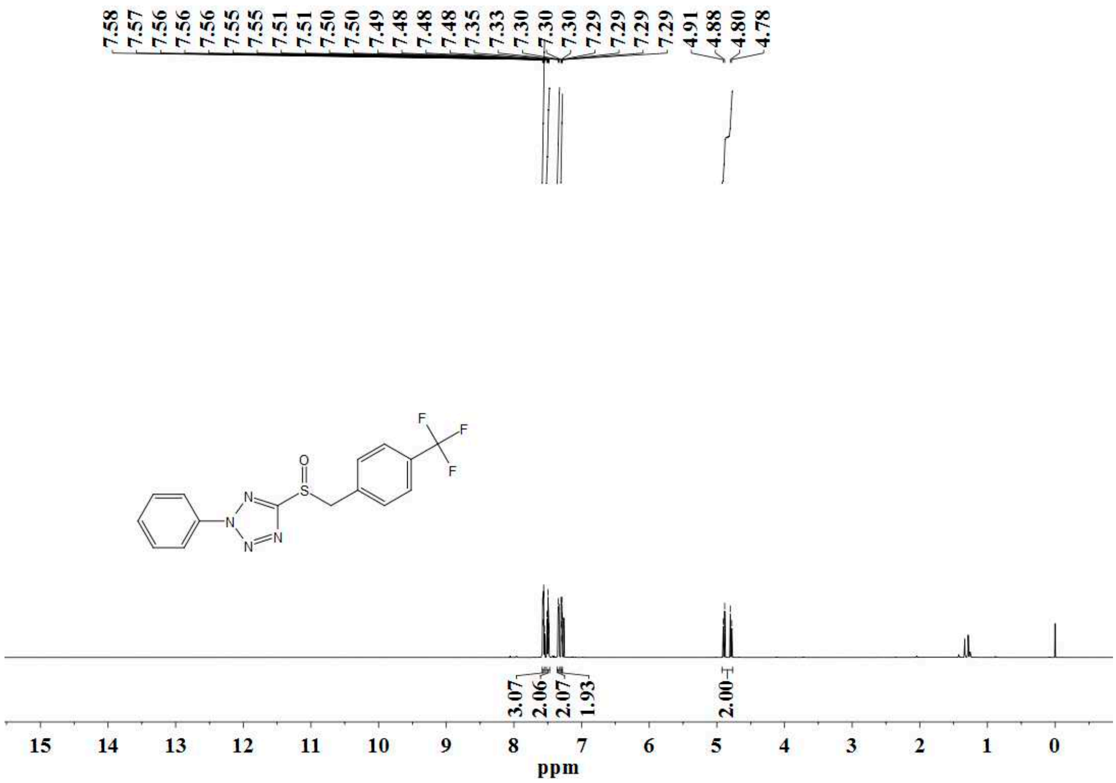

Figure S10-1. ^{1}H NMR spectrum of compound **4j**.

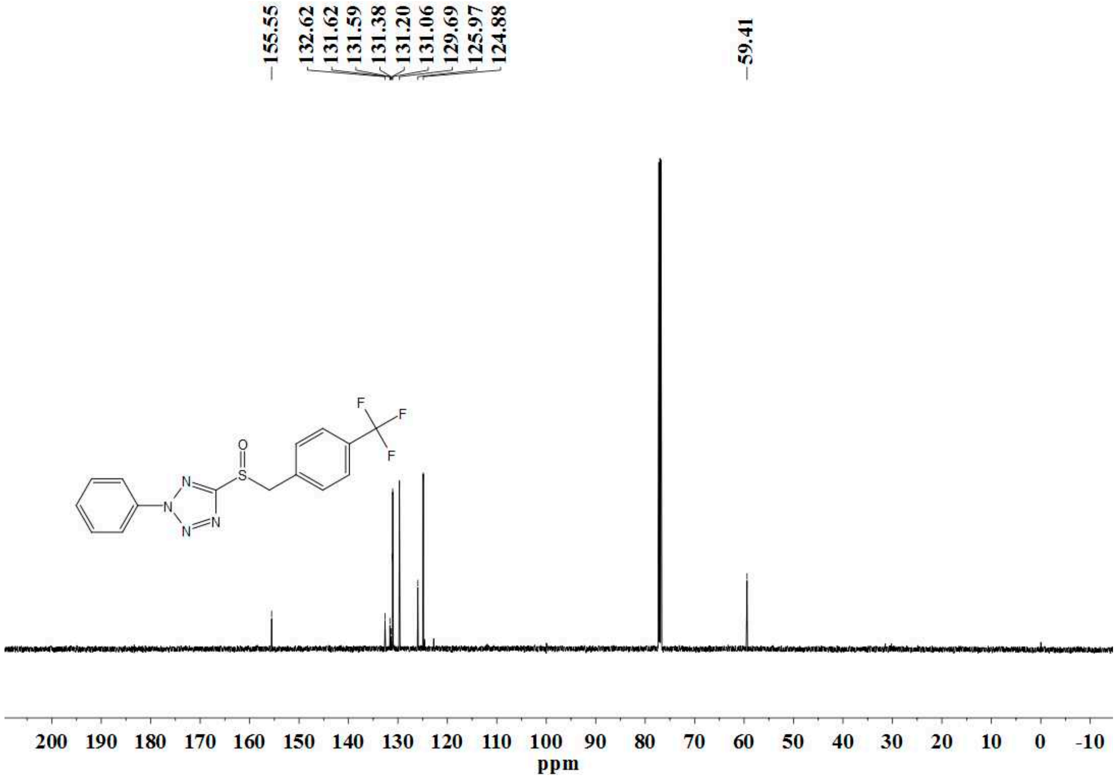

Figure S10-2. ^{13}C NMR spectrum of compound **4j**.

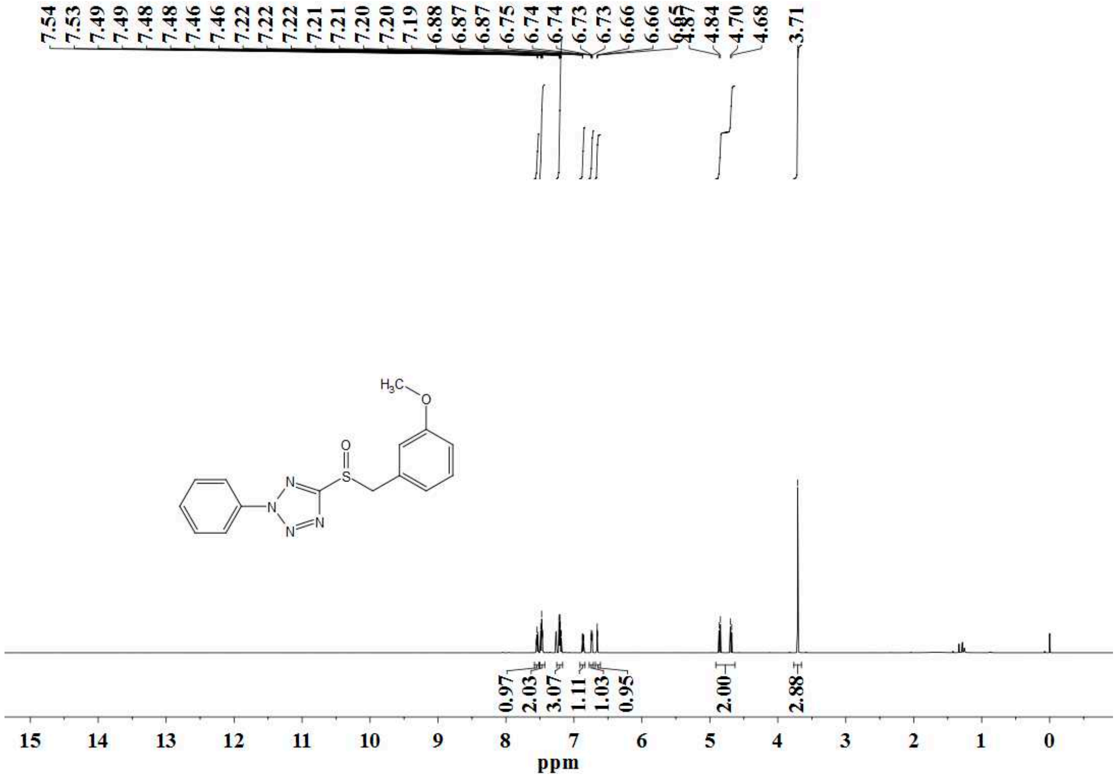

Figure S11-1. ^{1}H NMR spectrum of compound **4k**.

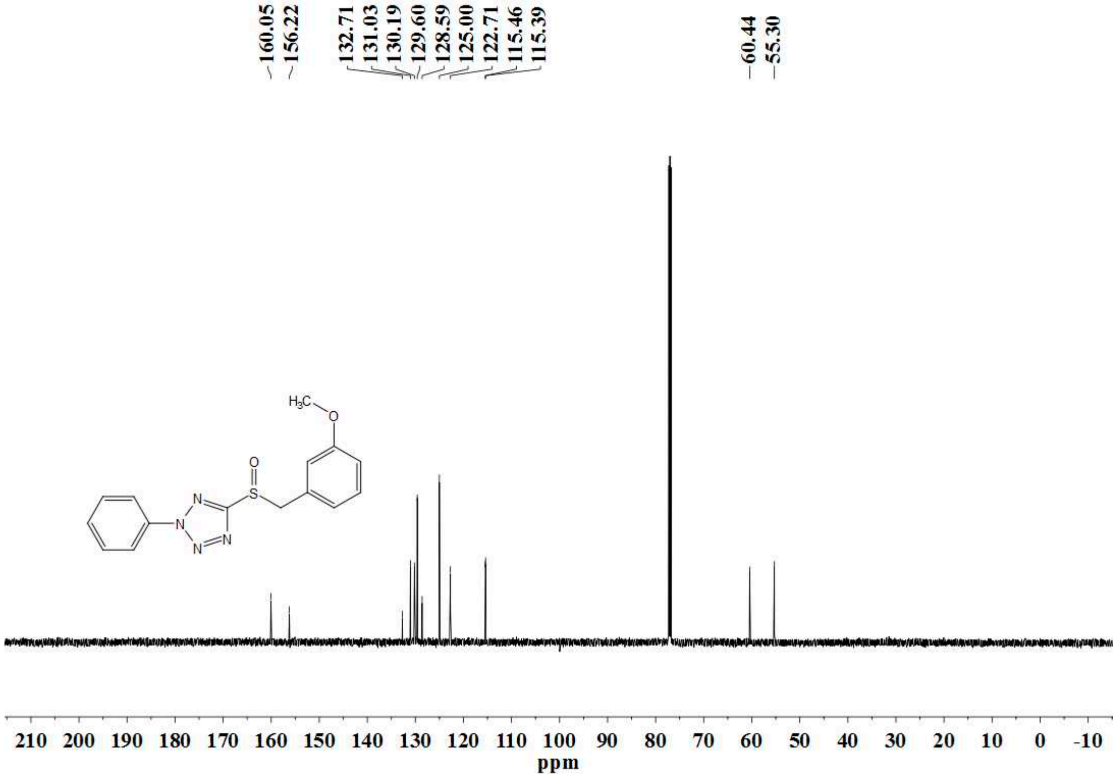

Figure S11-2. ^{13}C NMR spectrum of compound **4k**.

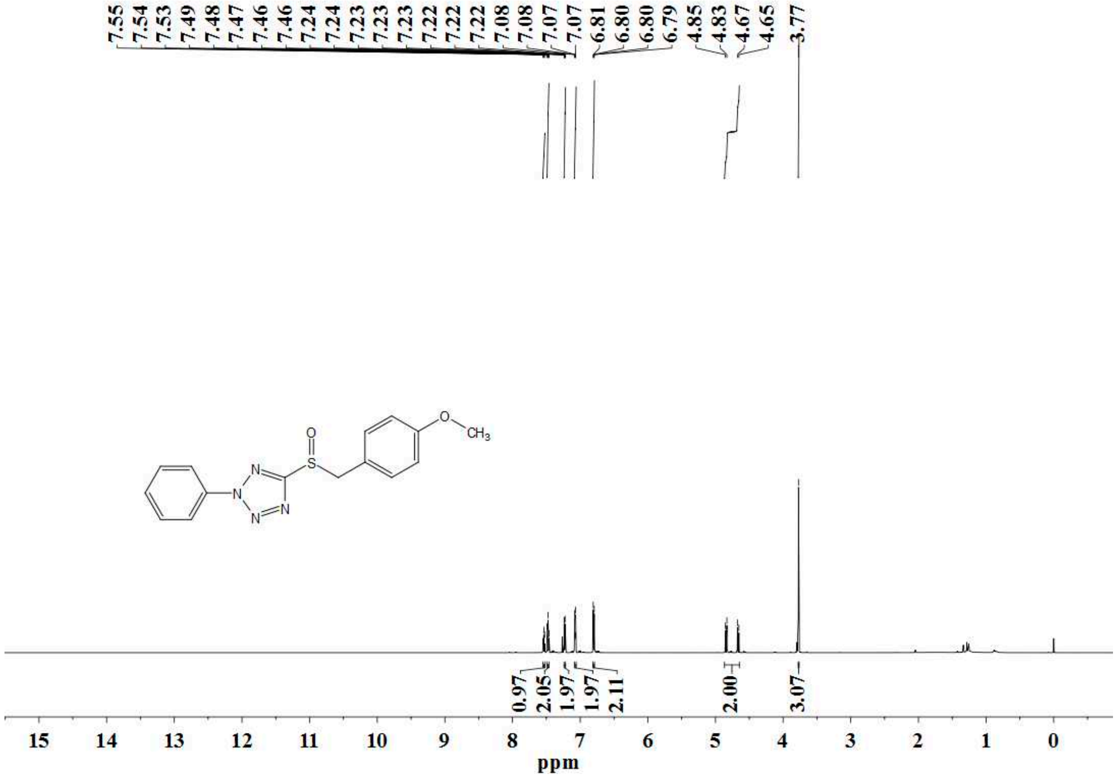

Figure S12-1. ^{1}H NMR spectrum of compound **4l**.

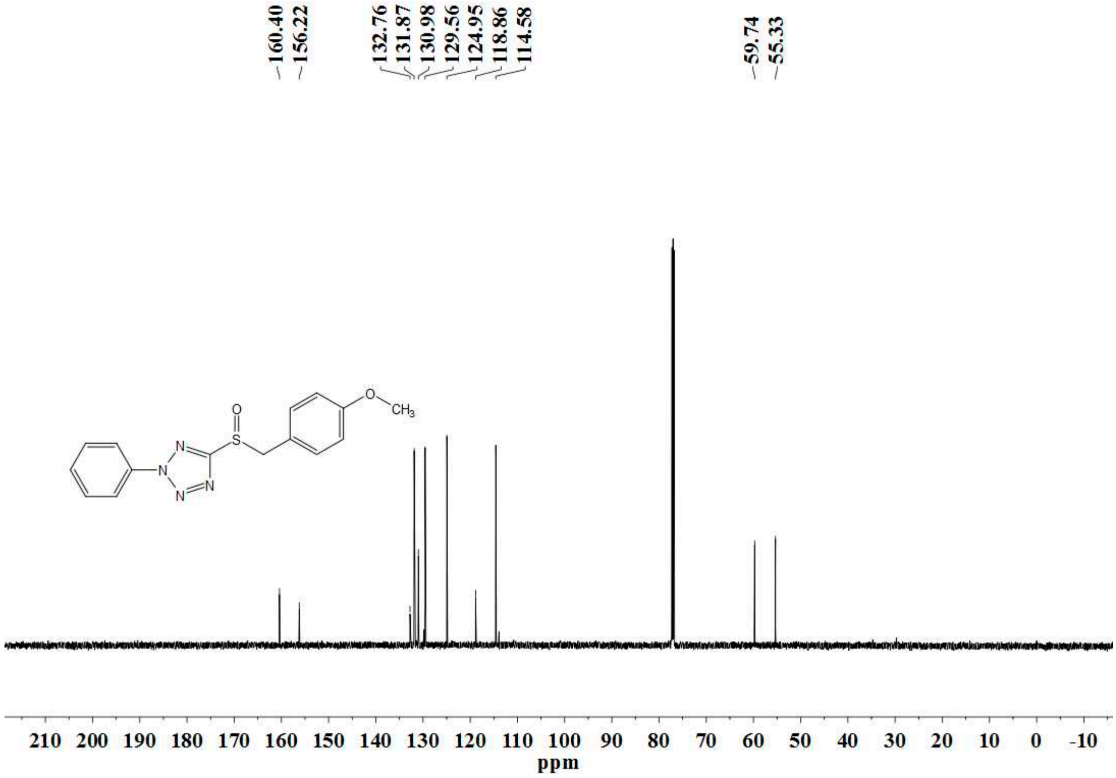

Figure S12-2. ^{13}C NMR spectrum of compound **4l**.

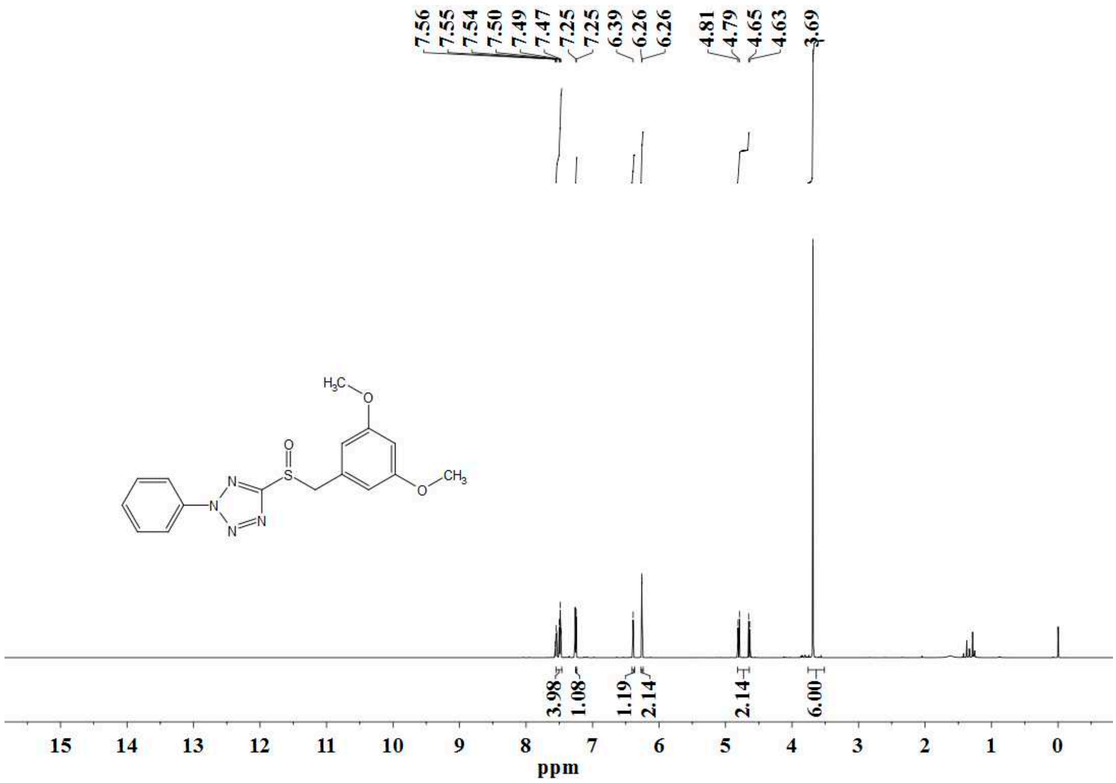

Figure S13-1. ^{1}H NMR spectrum of compound **4m**.

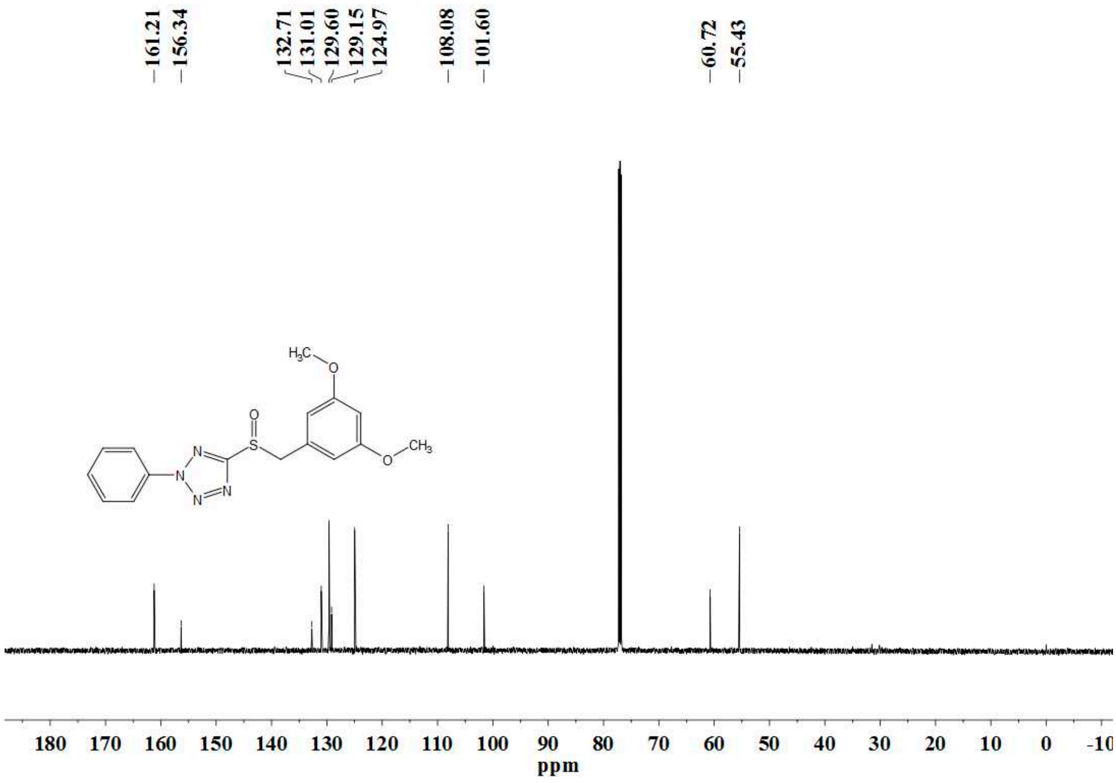

Figure S13-2. ^{13}C NMR spectrum of compound **4m**.

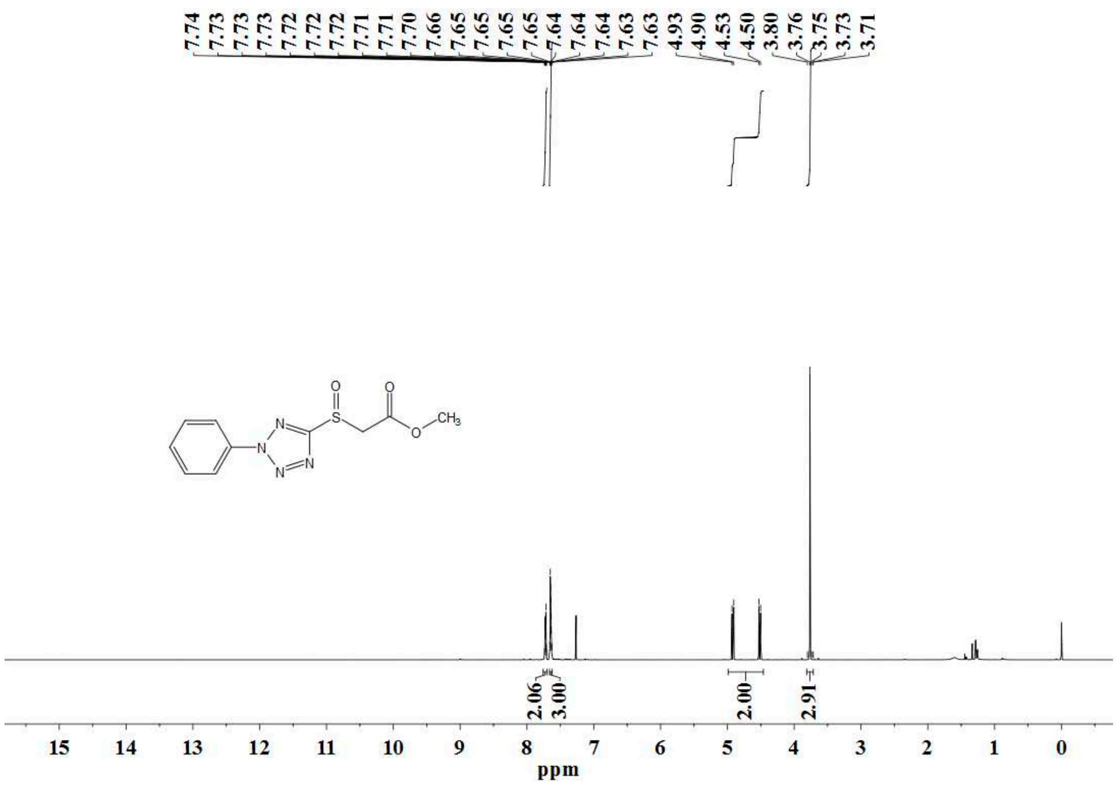

Figure S14-1. ^{1}H NMR spectrum of compound **4n**.

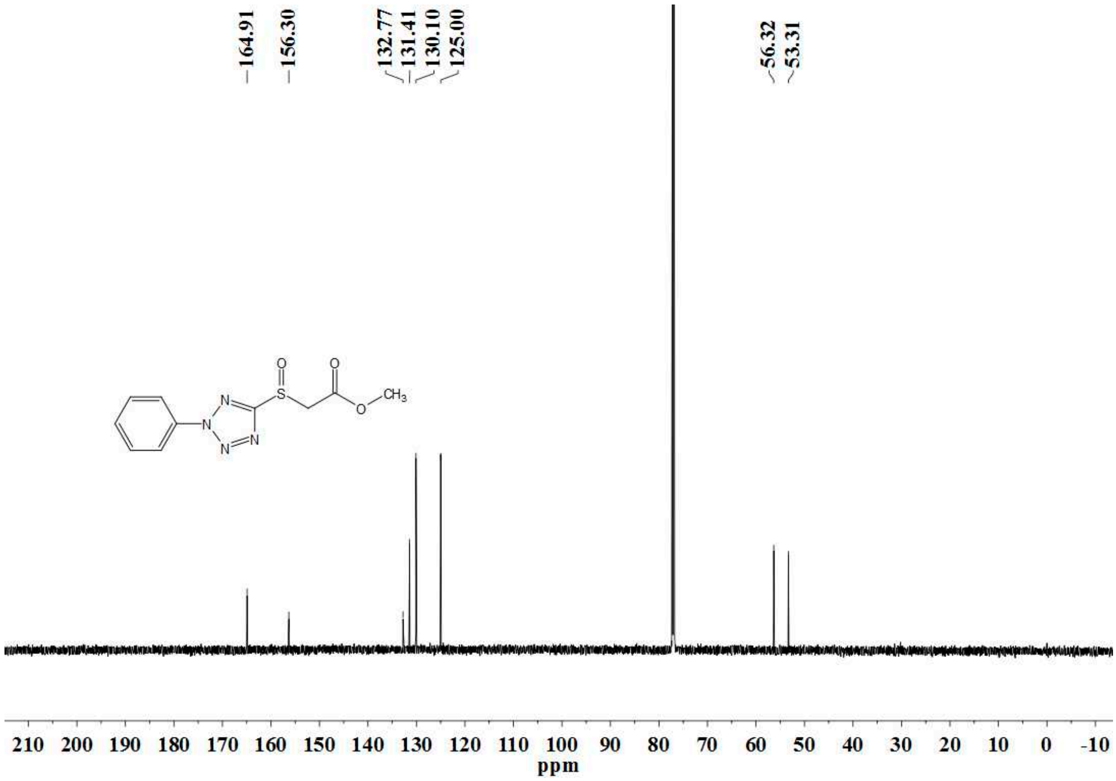

Figure S14-2. ^{13}C NMR spectrum of compound **4n**.

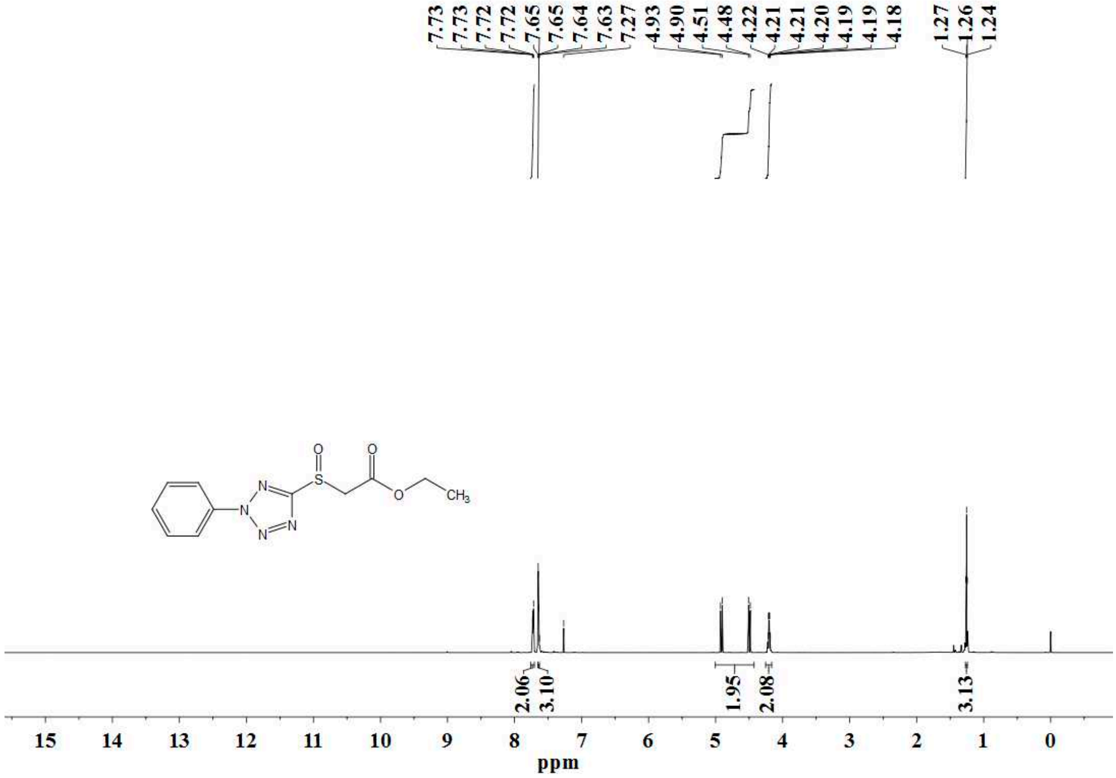

Figure S15-1. ^{1}H NMR spectrum of compound **4o**.

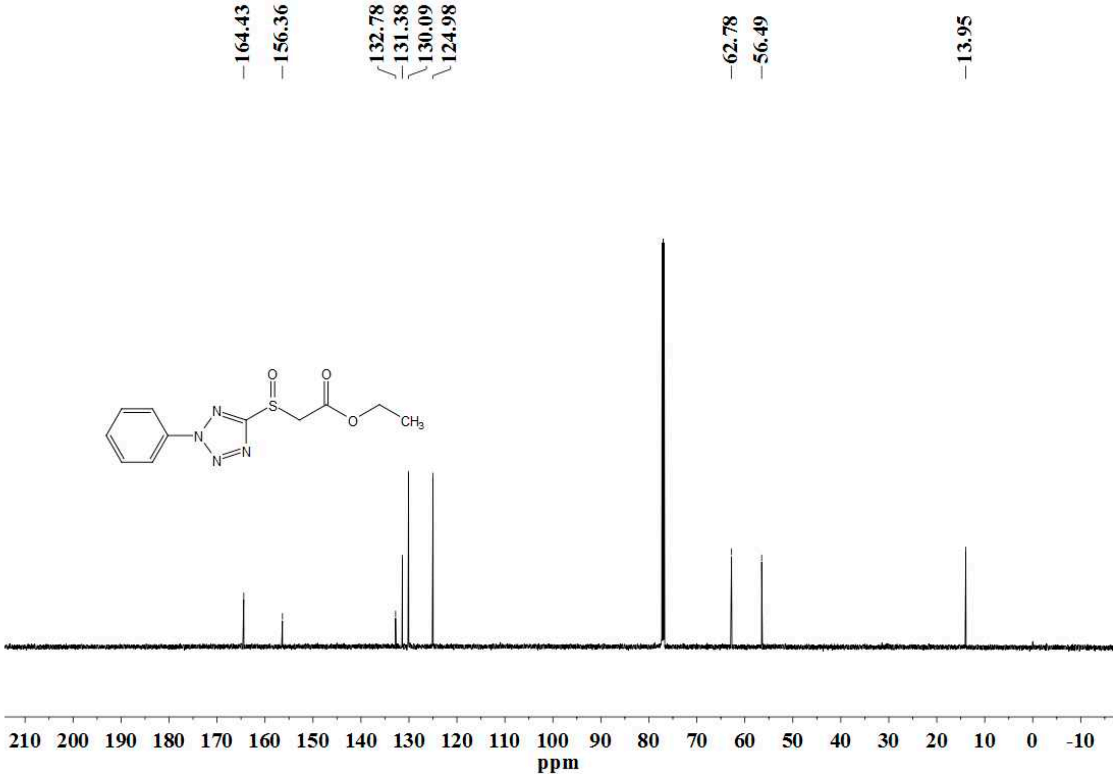

Figure S15-2. ^{13}C NMR spectrum of compound **4o**.

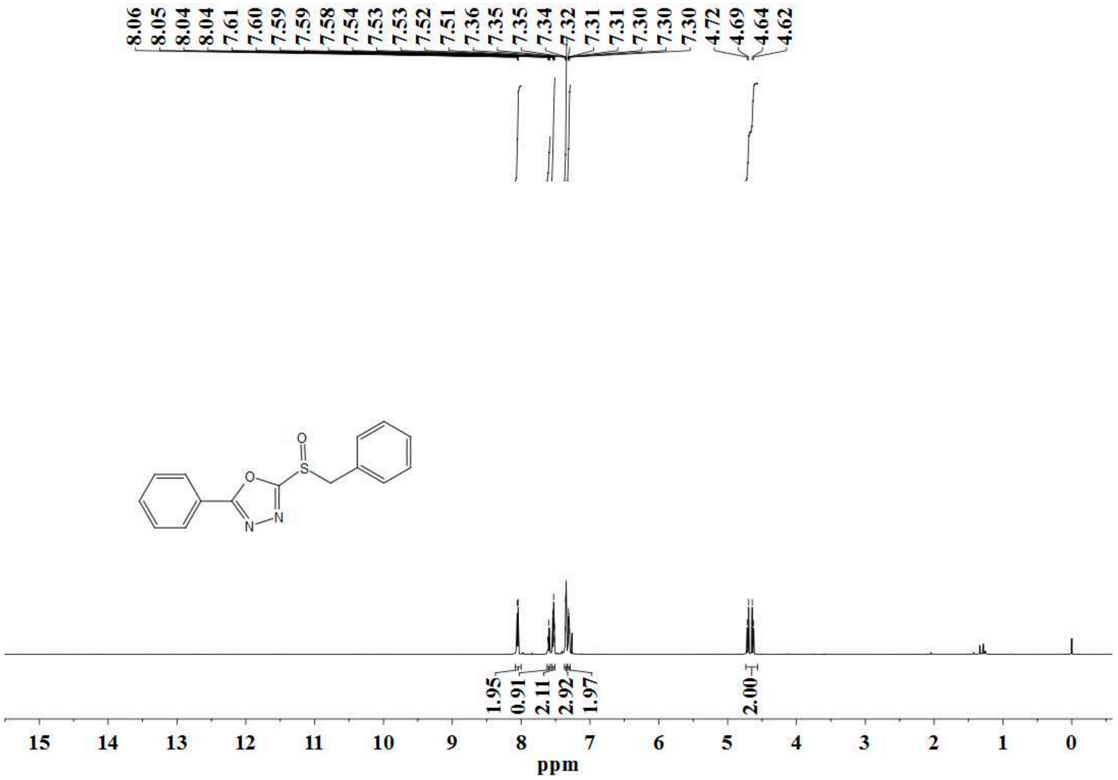

Figure S16-1. ^{1}H NMR spectrum of compound **5a**.

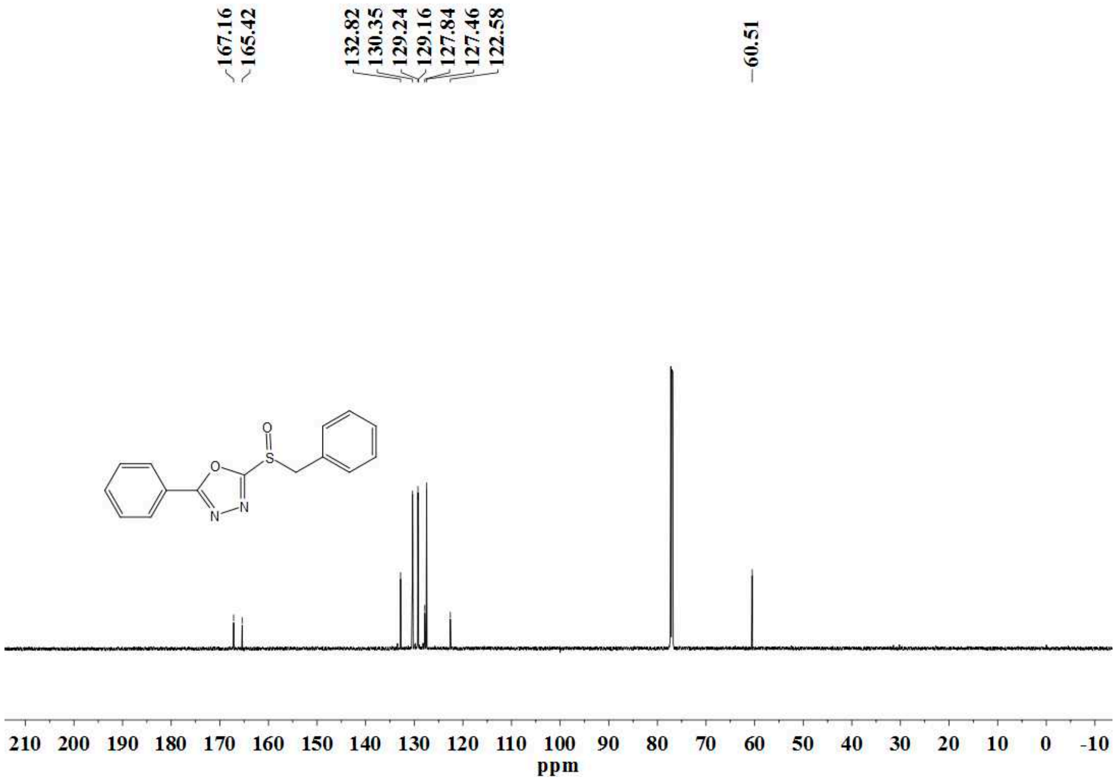

Figure S16-2. ^{13}C NMR spectrum of compound **5a**.

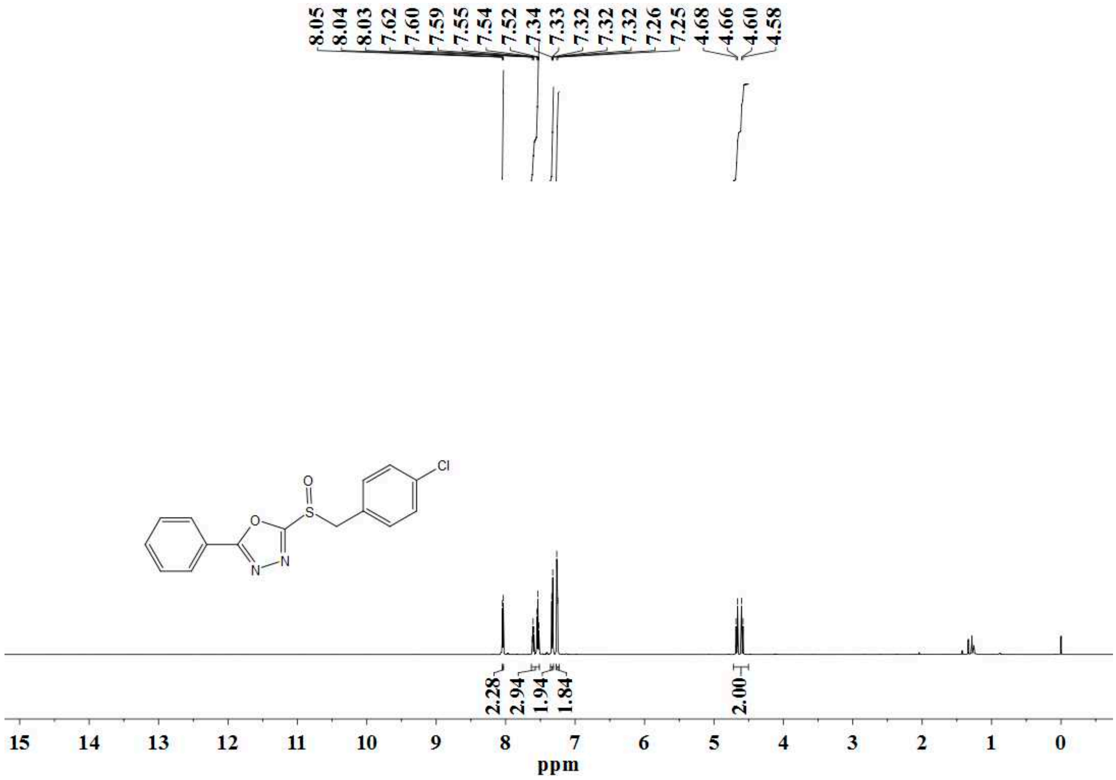

Figure S17-1. ^{1}H NMR spectrum of compound **5b**.

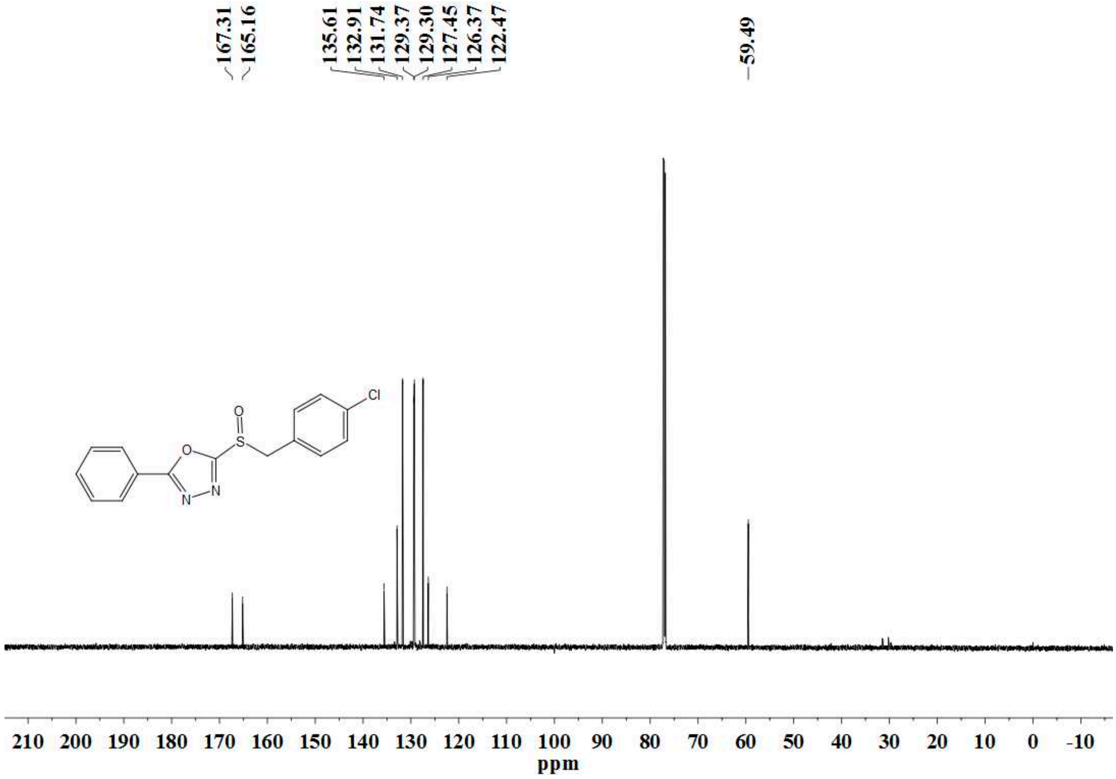

Figure S17-2. ^{13}C NMR spectrum of compound **5b**.

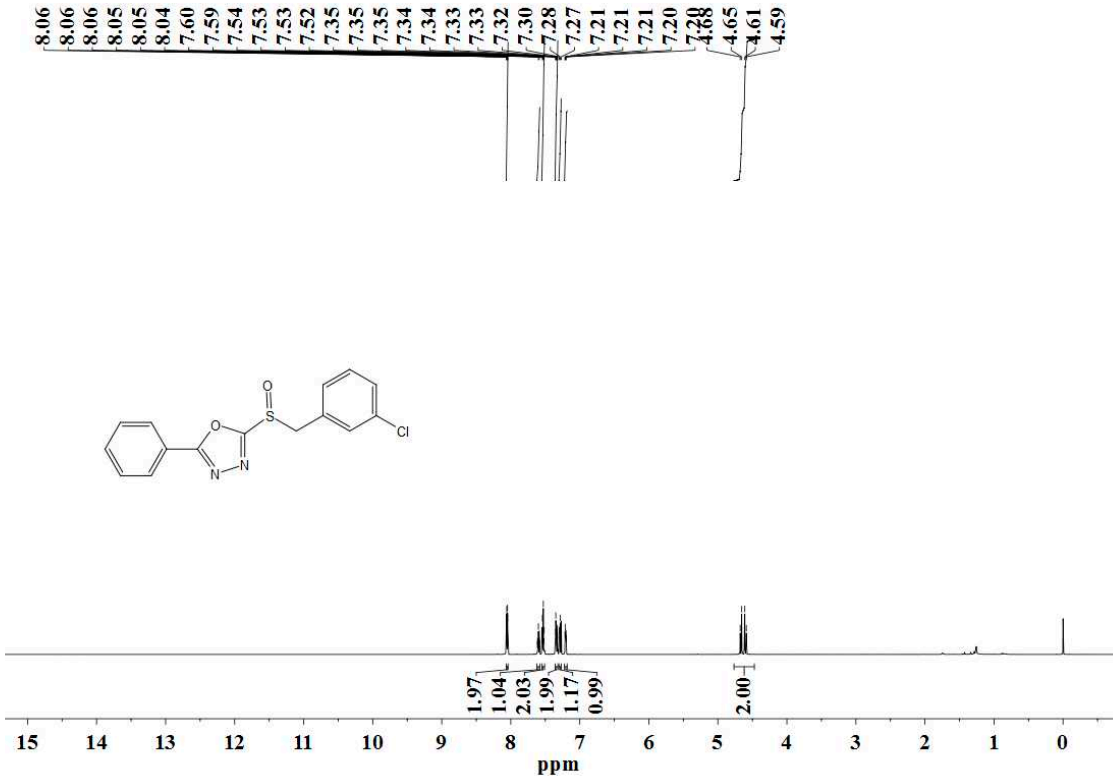

Figure S18-1. ^{1}H NMR spectrum of compound **5c**.

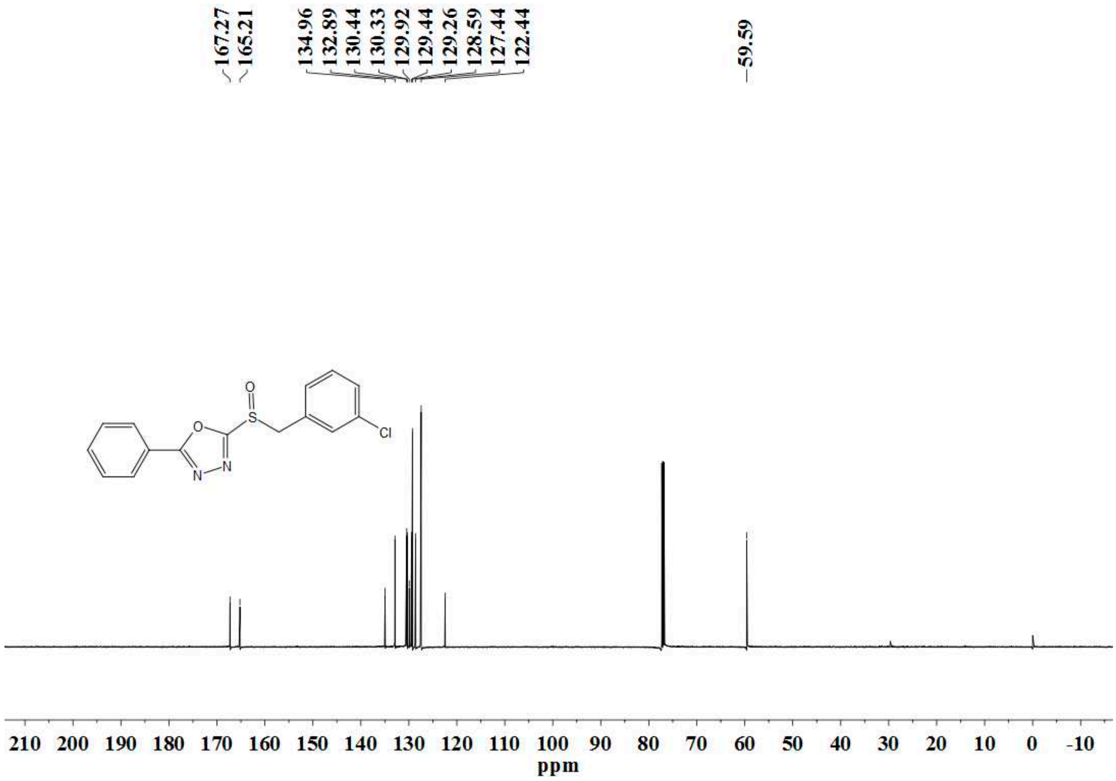

Figure S18-2. ^{13}C NMR spectrum of compound **5c**.

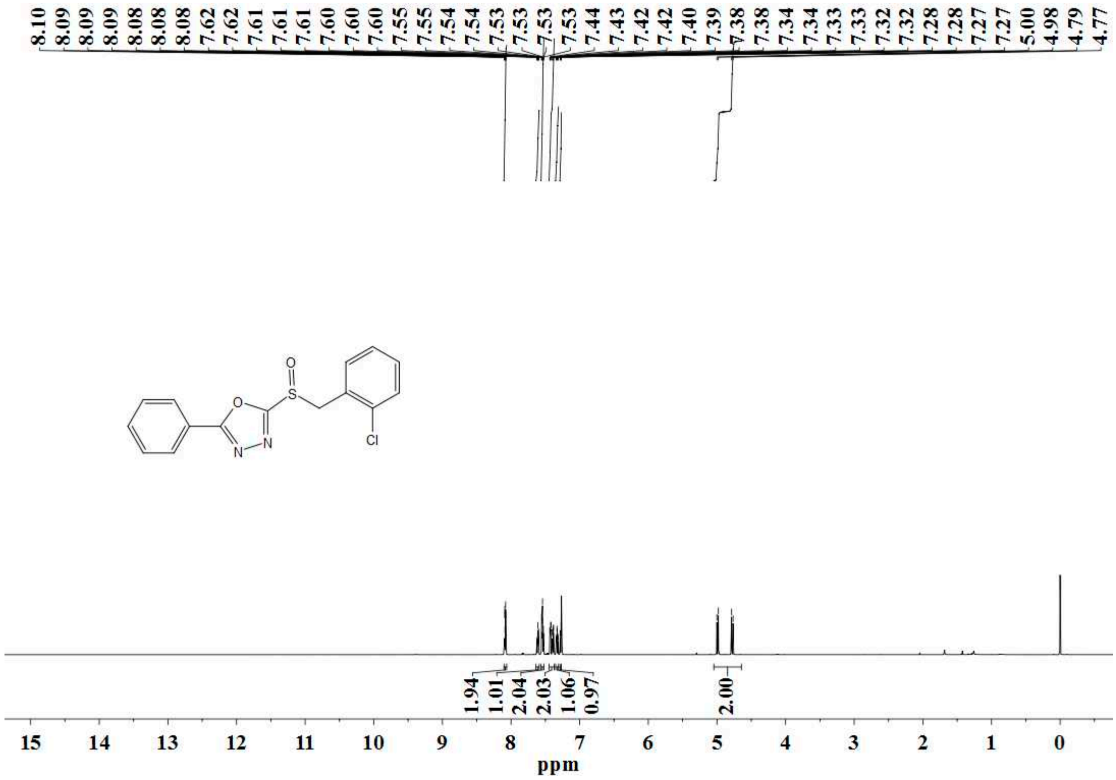

Figure S19-1. ^{1}H NMR spectrum of compound **5d**.

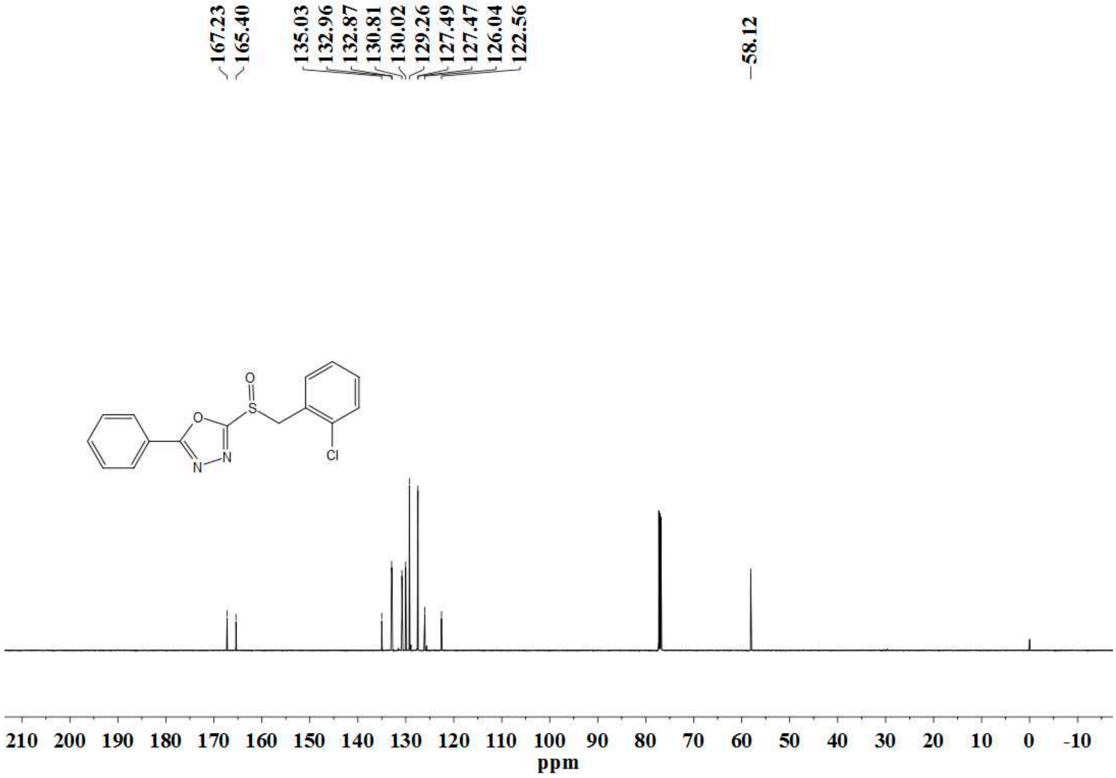

Figure S19-2. ^{13}C NMR spectrum of compound **5d**.

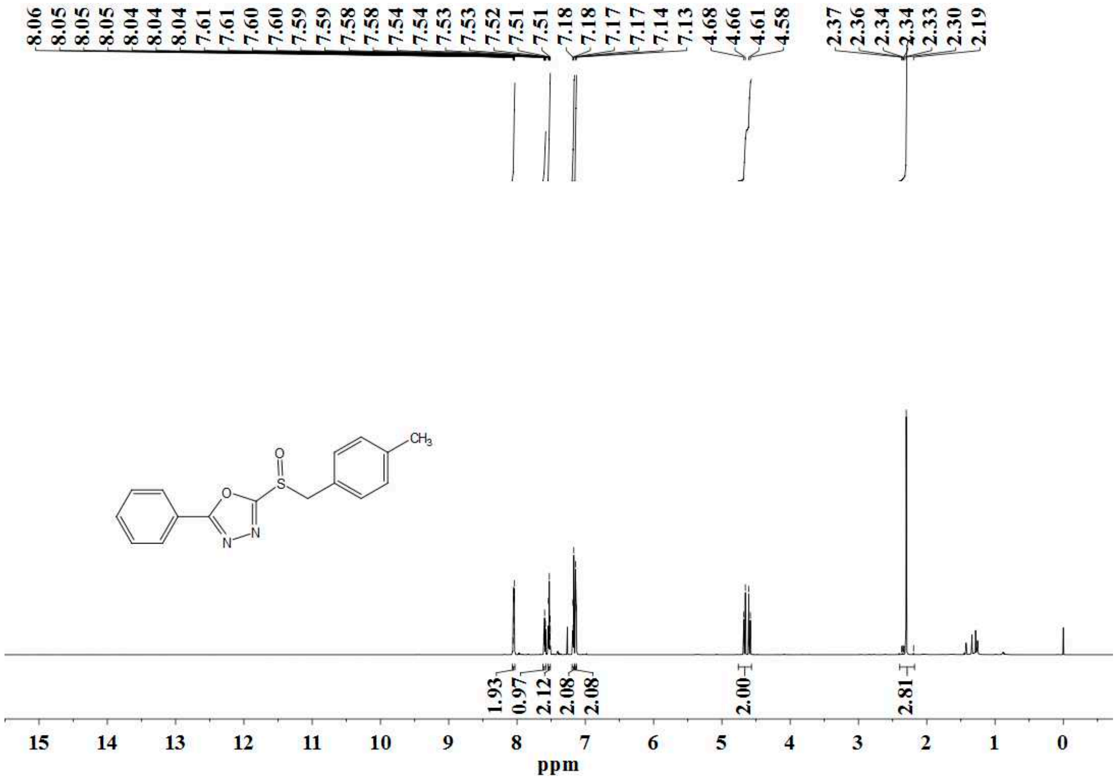

Figure S20-1. ^{1}H NMR spectrum of compound **5e**.

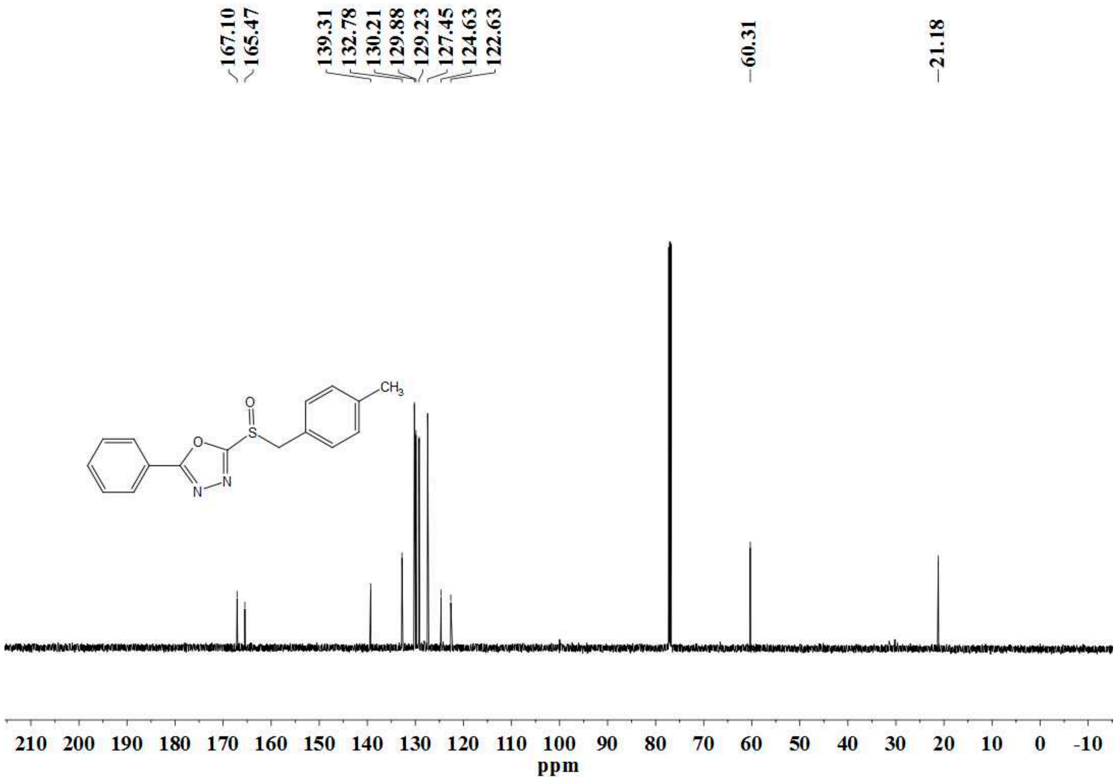

Figure S20-2. ^{13}C NMR spectrum of compound **5e**.

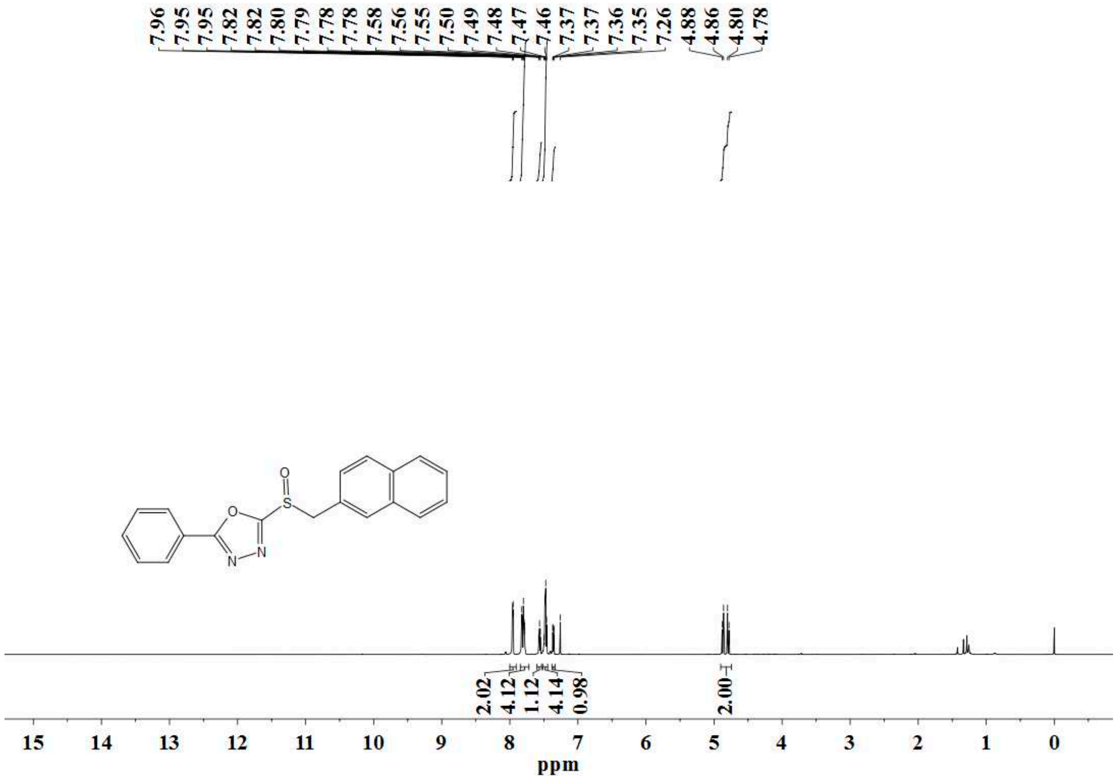

Figure S21-1. ^{1}H NMR spectrum of compound **5f**.

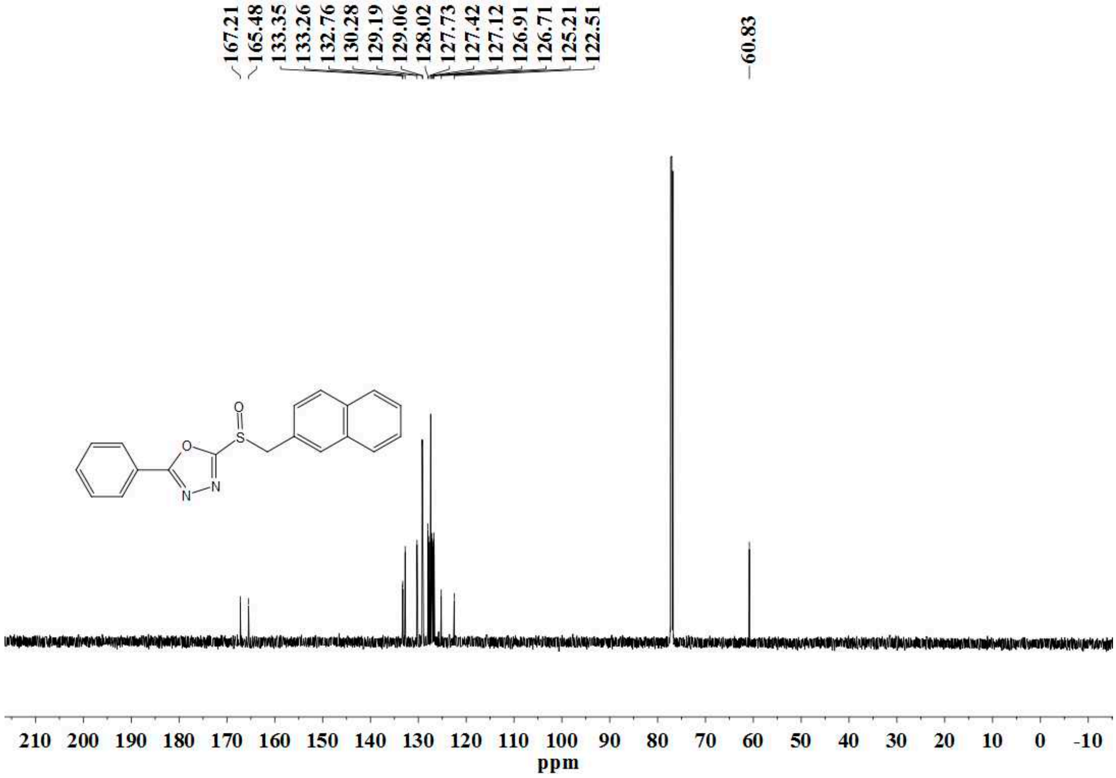

Figure S21-2. ^{13}C NMR spectrum of compound **5f**.

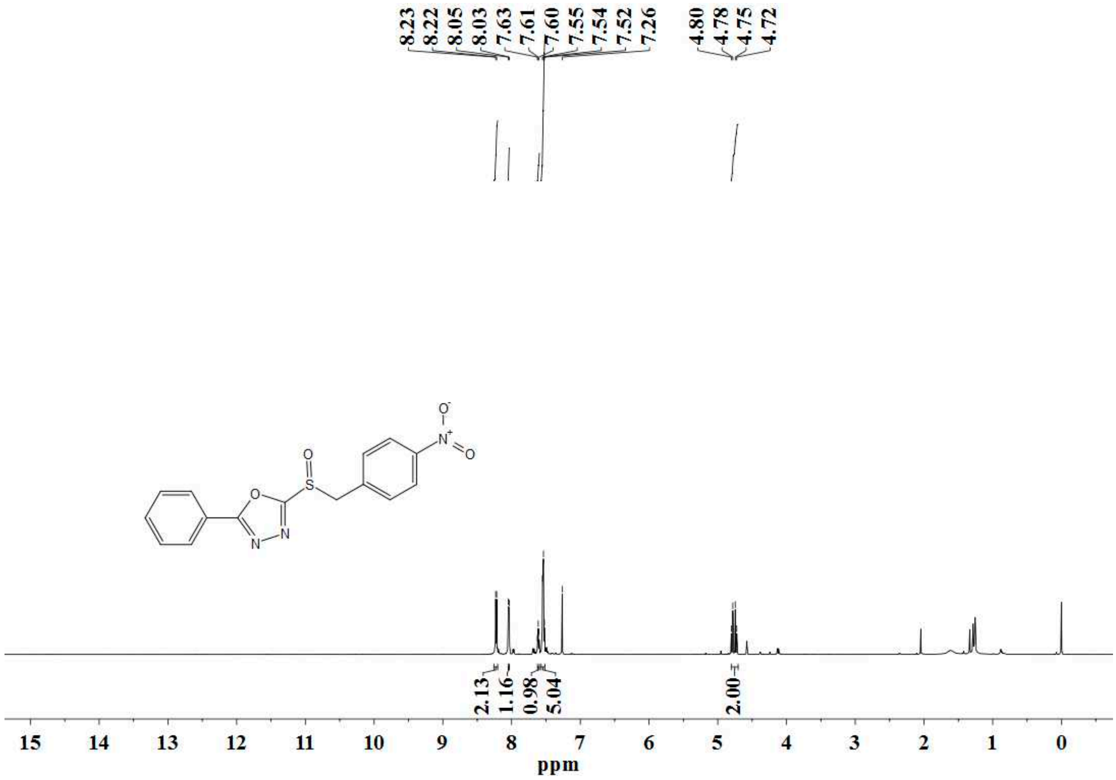

Figure S22-1. ^{1}H NMR spectrum of compound **5g**.

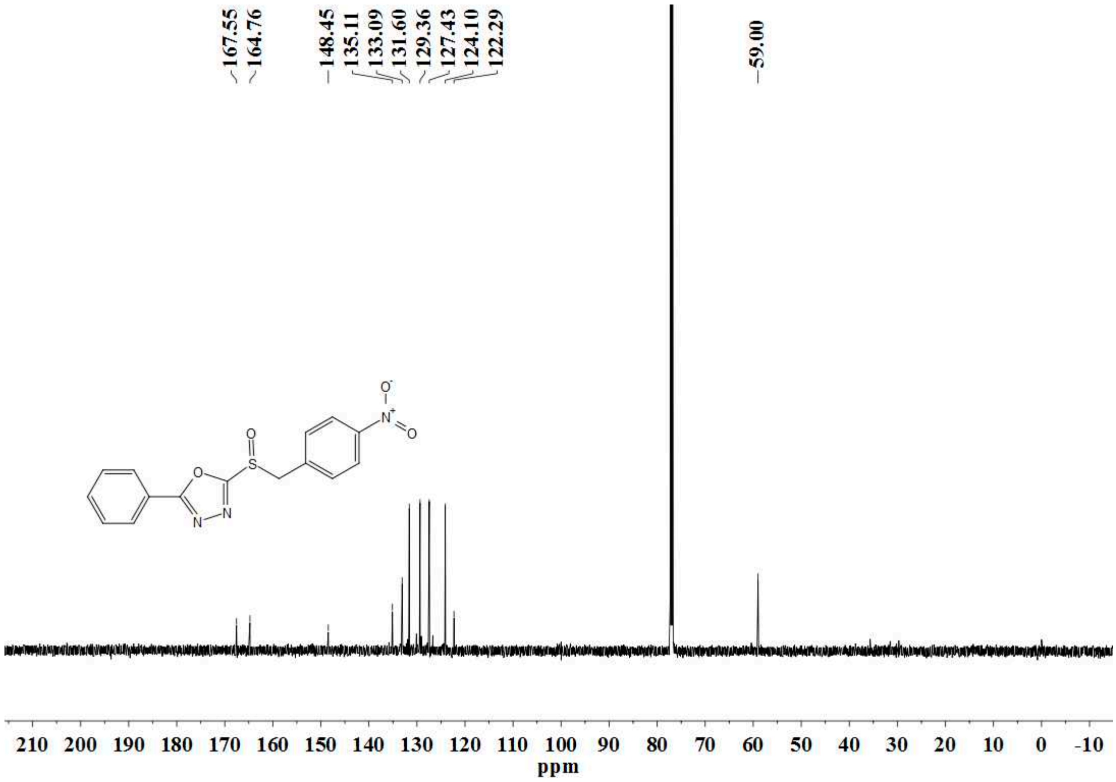

Figure S22-2. ^{13}C NMR spectrum of compound **5g**.

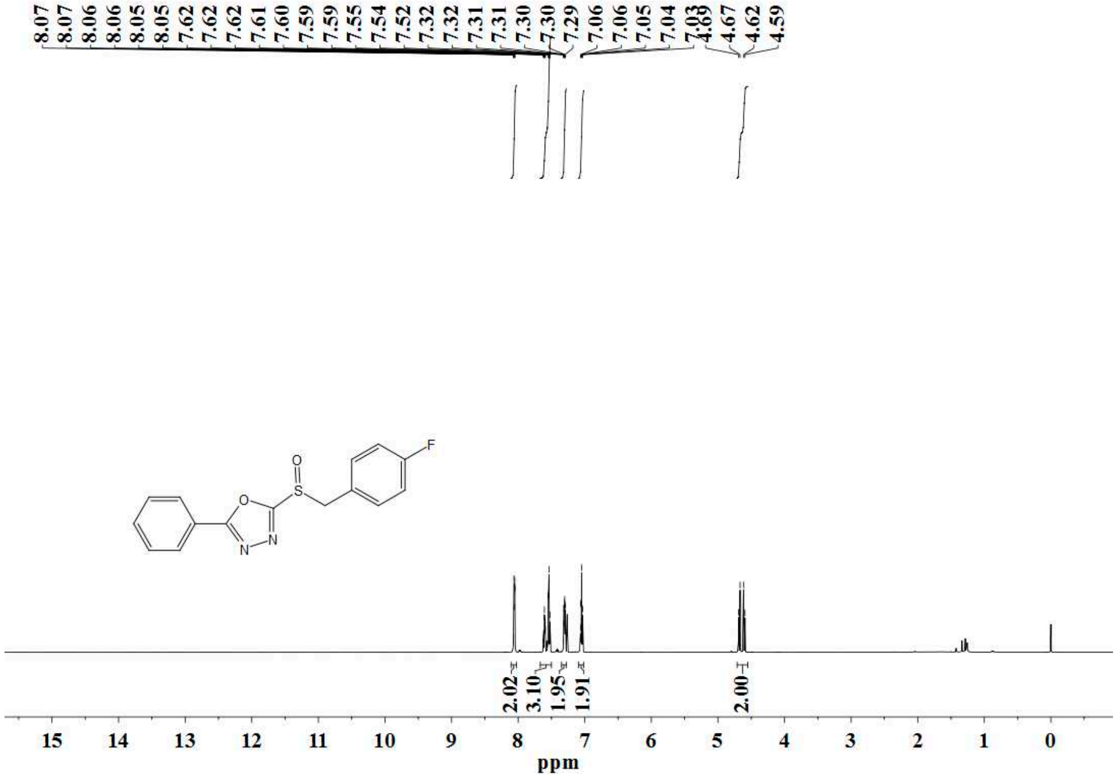

Figure S23-1. ^{1}H NMR spectrum of compound **5h**.

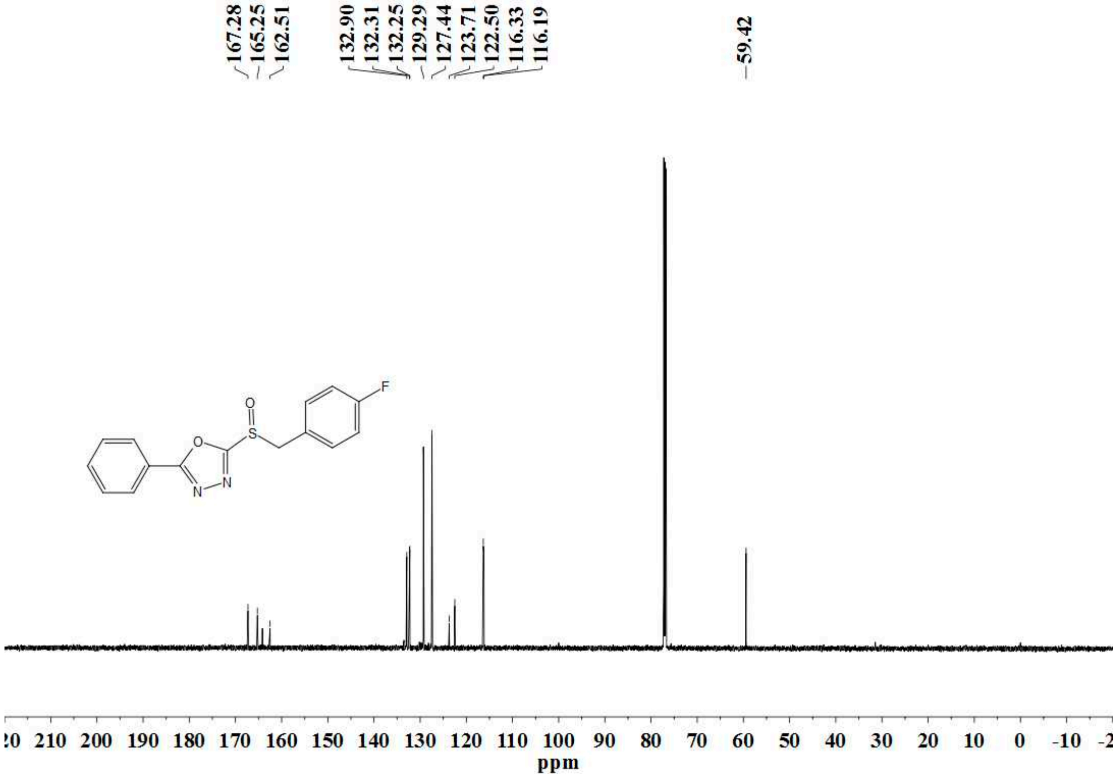

Figure S23-2. ^{13}C NMR spectrum of compound **5h**.

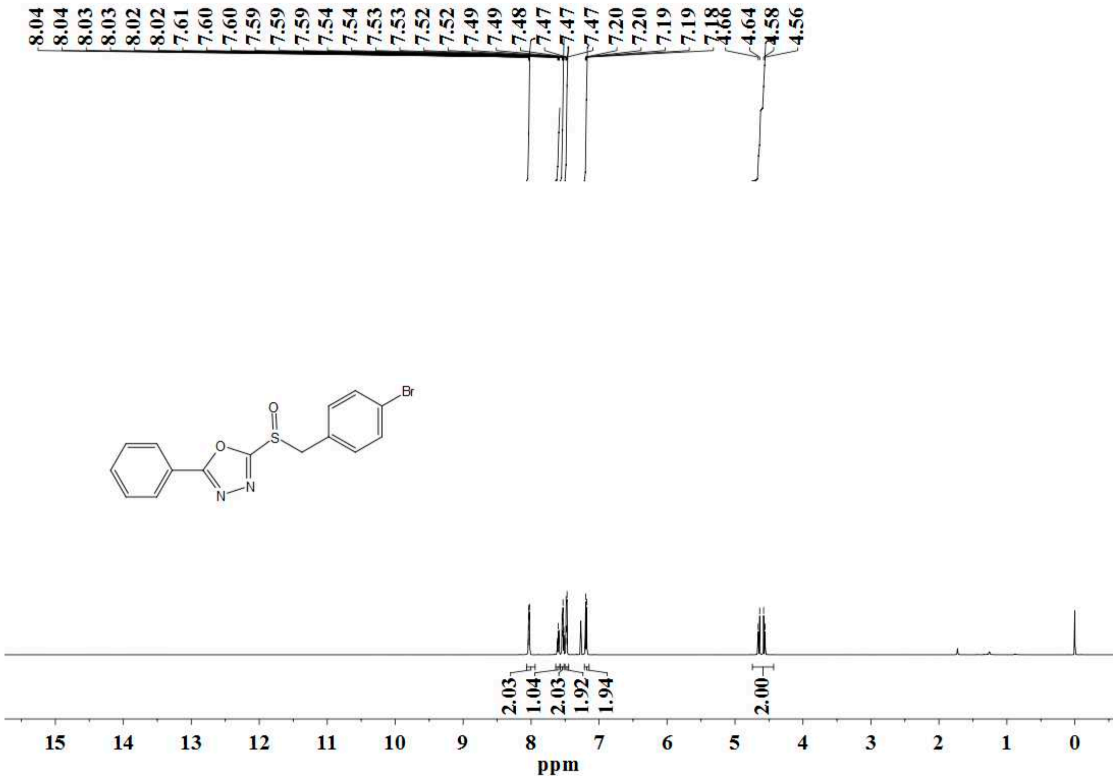

Figure S24-1. ^{1}H NMR spectrum of compound **5i**.

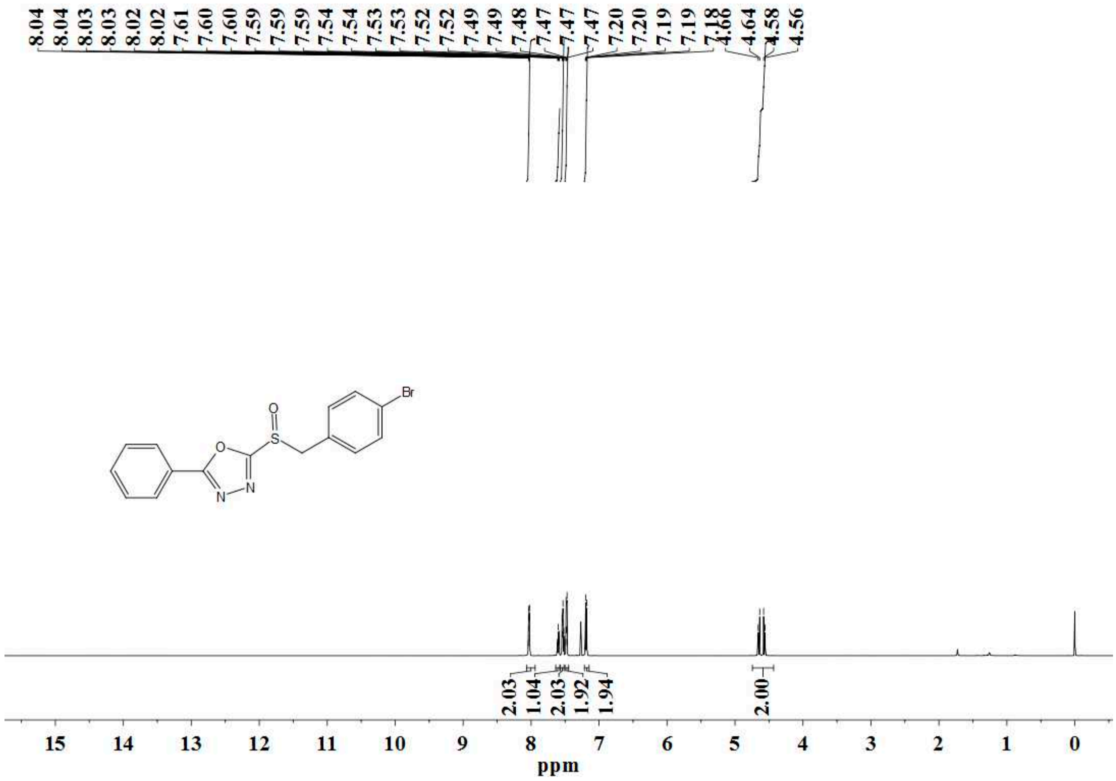
8.04
8.04
8.03
8.03
8.02
8.02
7.61
7.60
7.60
7.59
7.59
7.59
7.54
7.54
7.53
7.53
7.52
7.52
7.49
7.49
7.48
7.47
7.47
7.47
7.20
7.20
7.19
7.19
7.18
4.66
4.64
4.58
4.56
Br
O
S
O
N
N
2.03
1.04
2.03
1.92
1.94
2.00
15 14 13 12 11 10 9 8 7 6 5 4 3 2 1 0
ppm

Figure S24-2. ^{13}C NMR spectrum of compound **5i**.

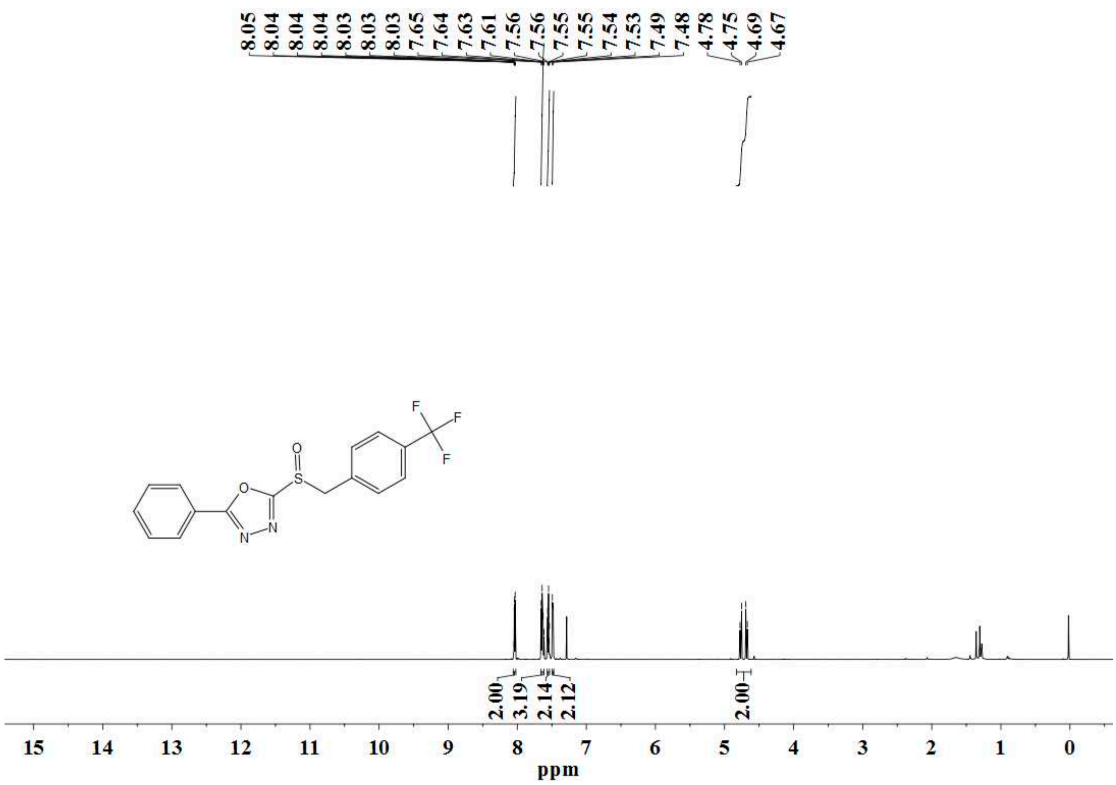

Figure S25-1. ^{1}H NMR spectrum of compound **5j**.

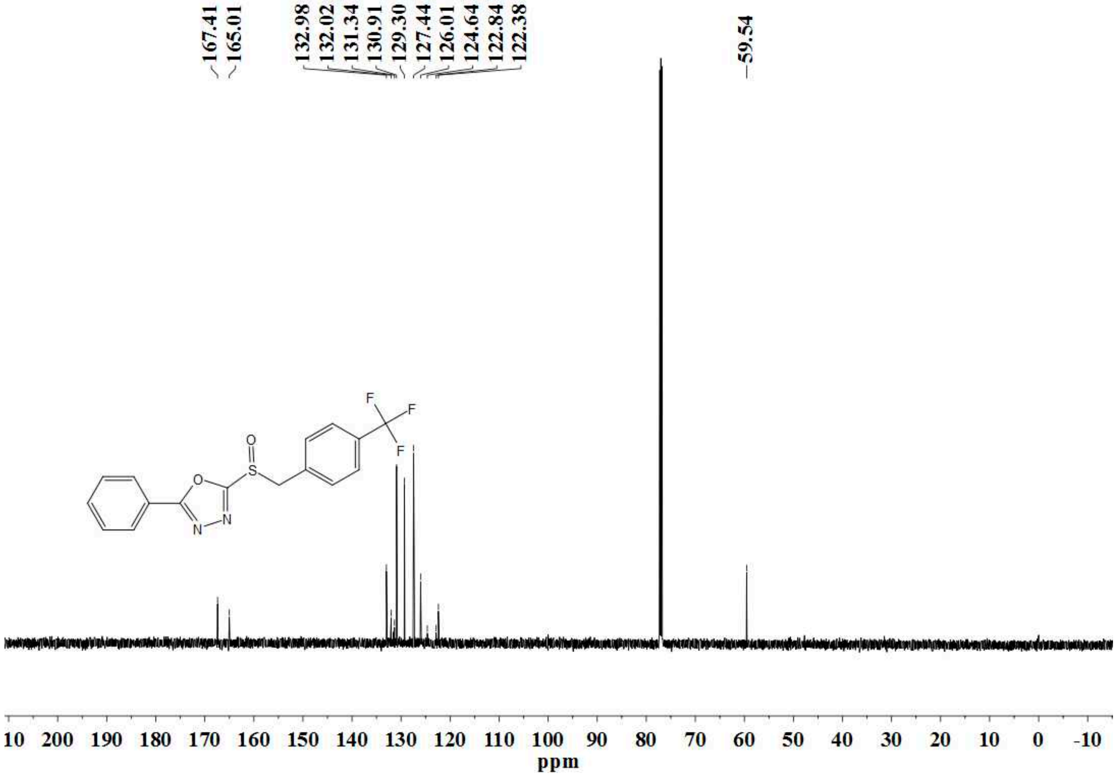

Figure S25-2. ^{13}C NMR spectrum of compound **5j**.

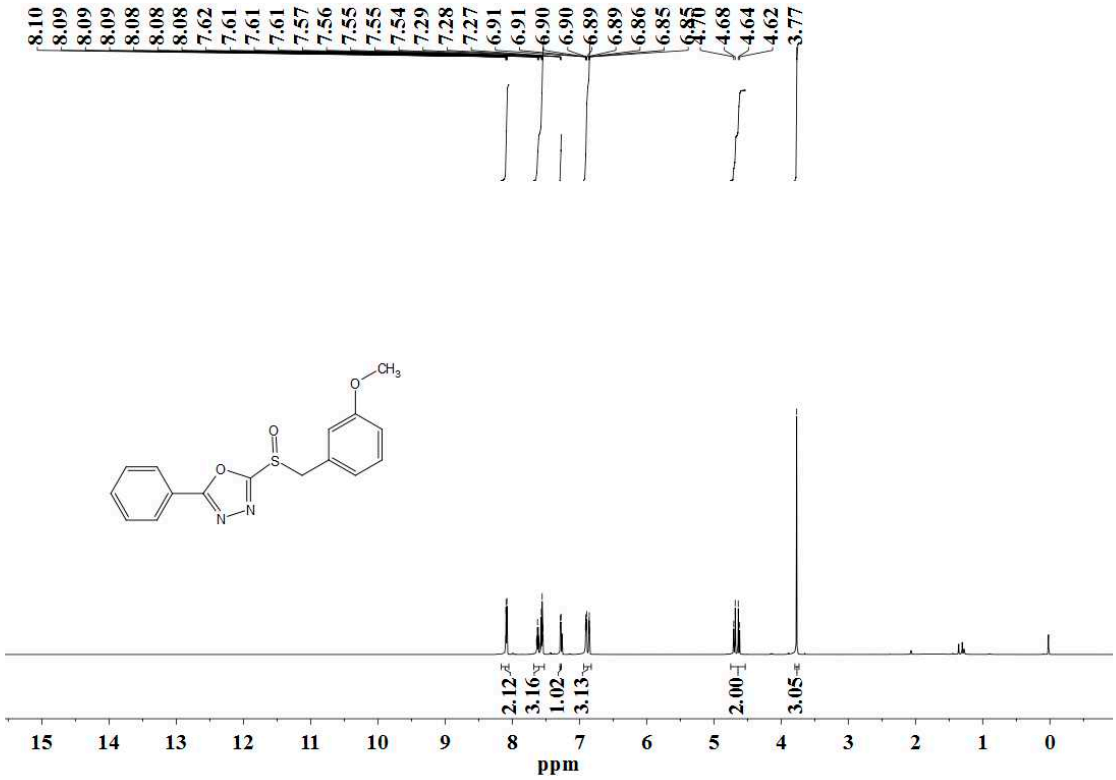

Figure S26-1. ^{1}H NMR spectrum of compound **5k**.

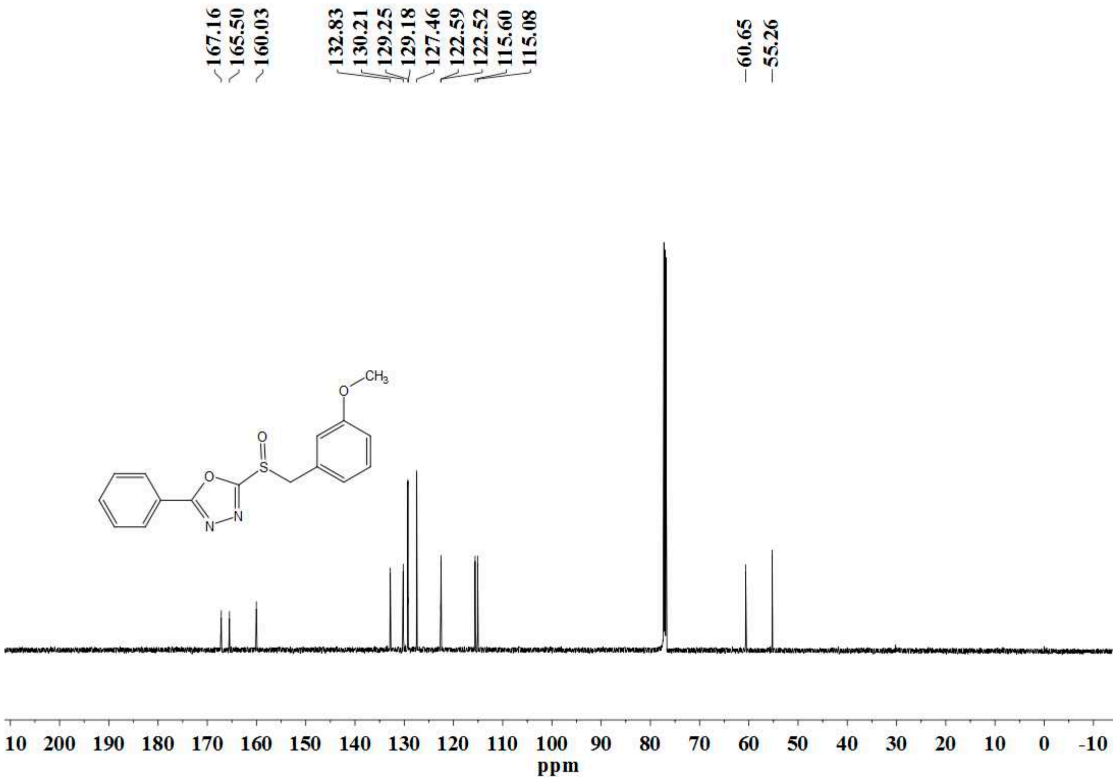

Figure S26-2. ^{13}C NMR spectrum of compound **5k**.

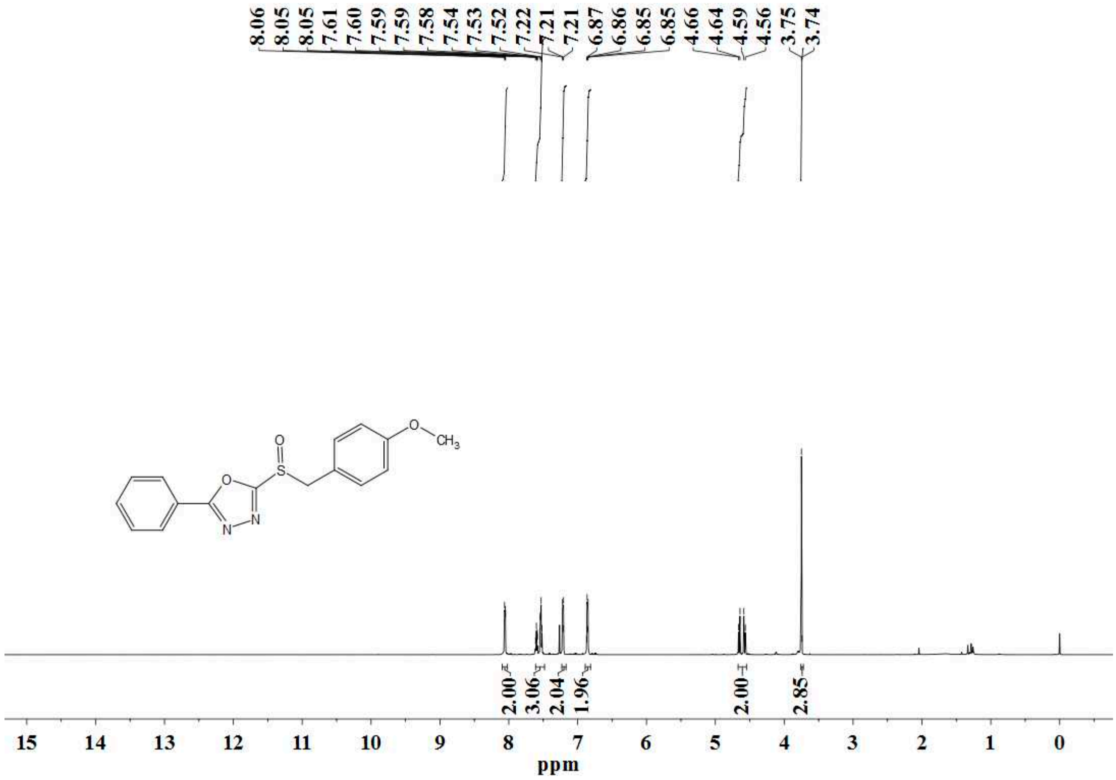

Figure S27-1. ^{1}H NMR spectrum of compound **5l**.

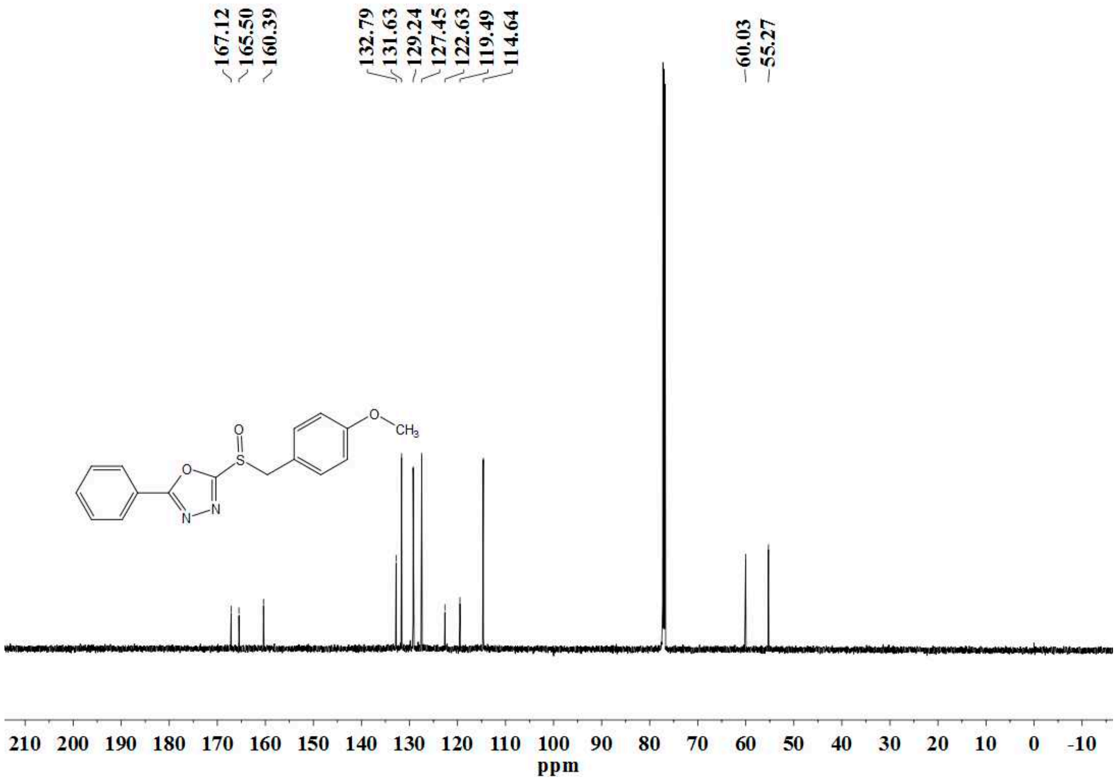

Figure S27-2. ^{13}C NMR spectrum of compound **5l**.

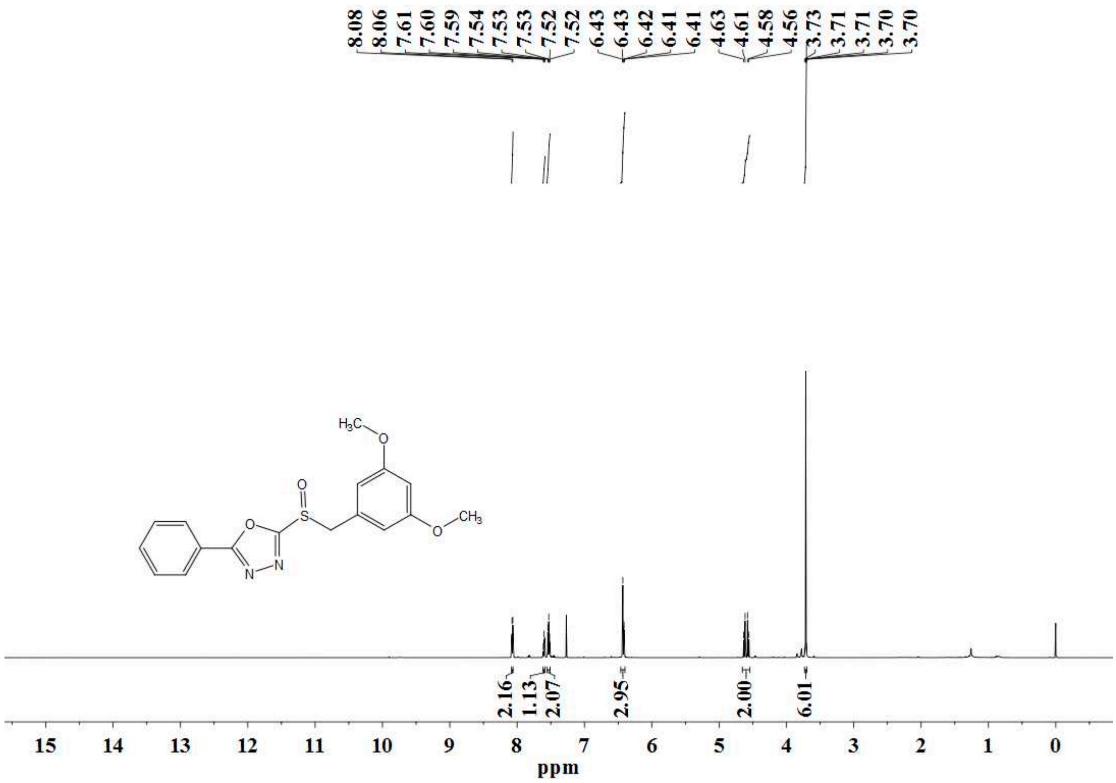

Figure S28-1. ^{1}H NMR spectrum of compound **5m**.

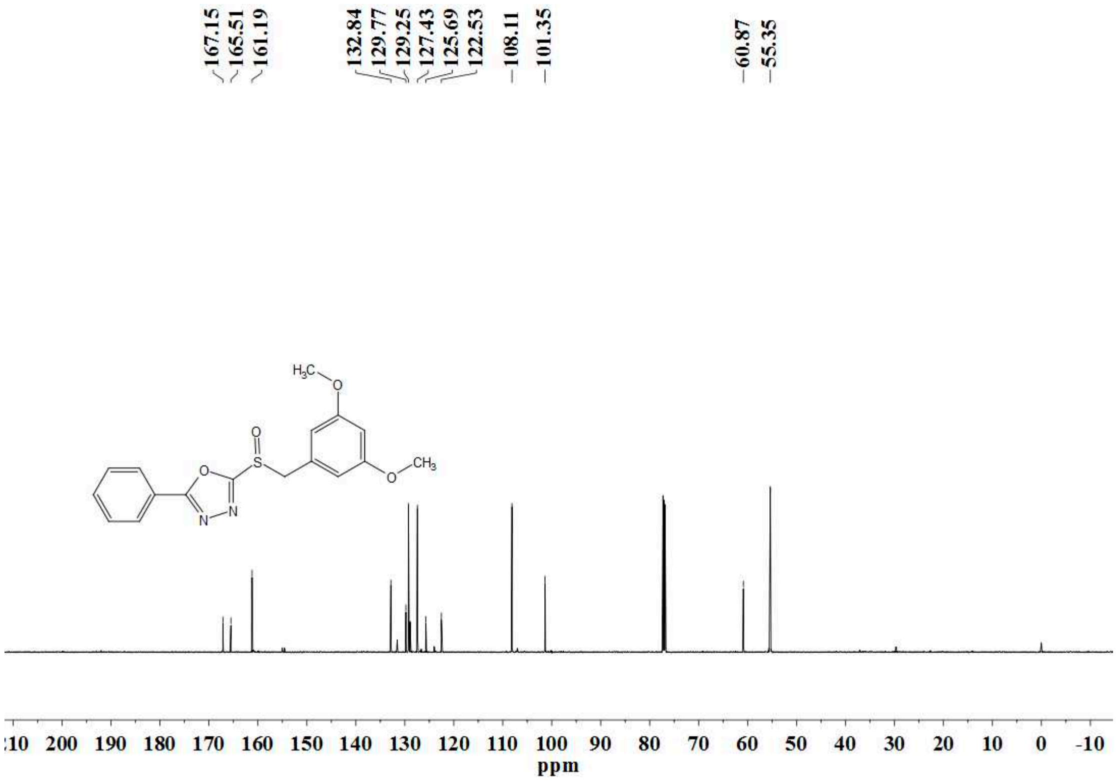

Figure S28-2. ^{13}C NMR spectrum of compound **5m**.

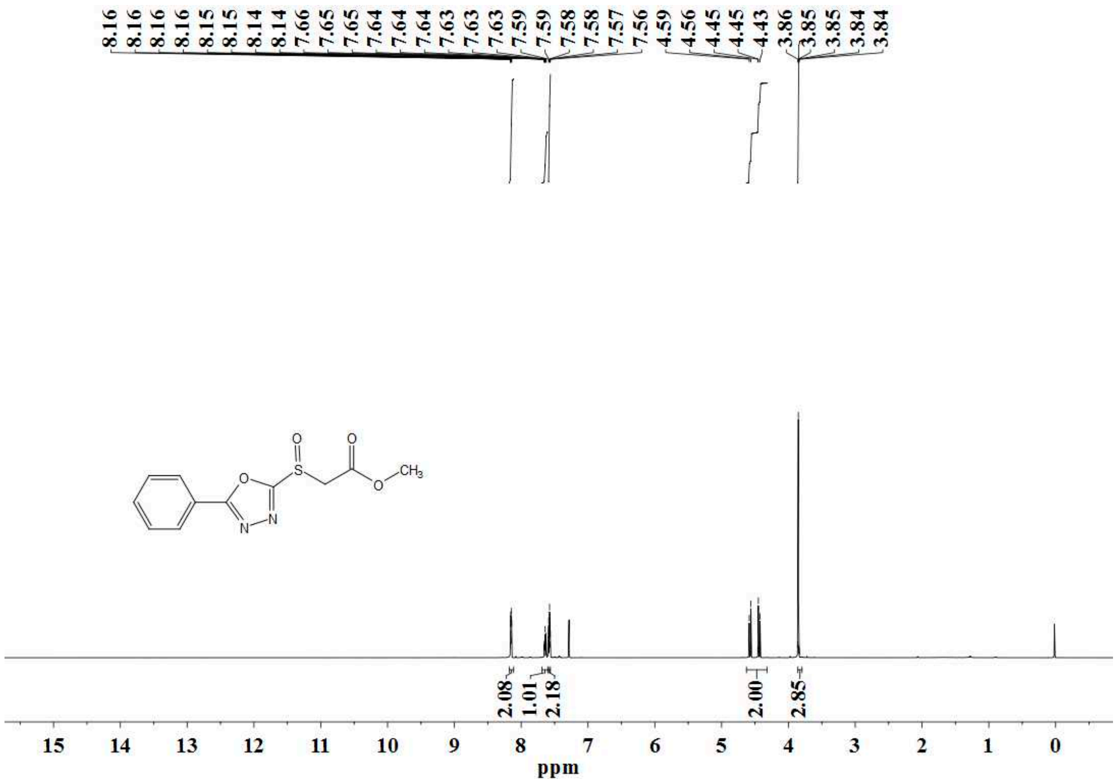

Figure S29-1. ^{1}H NMR spectrum of compound **5n**.

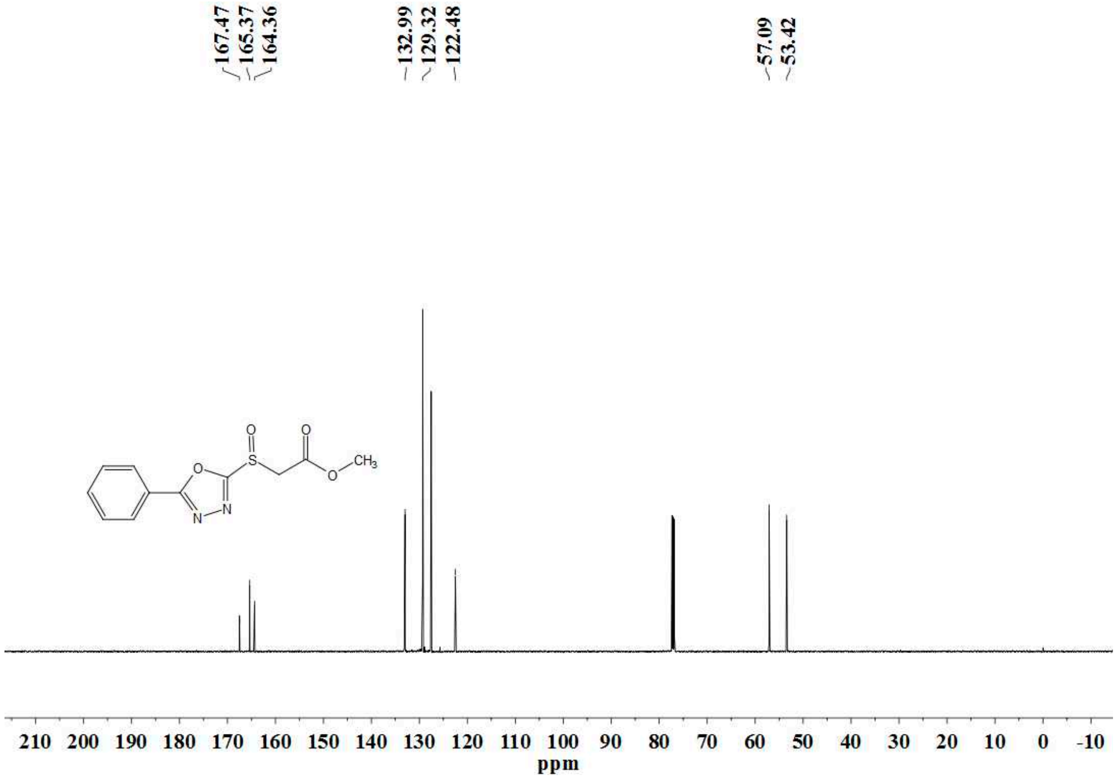

Figure S29-2. ^{13}C NMR spectrum of compound **5n**.

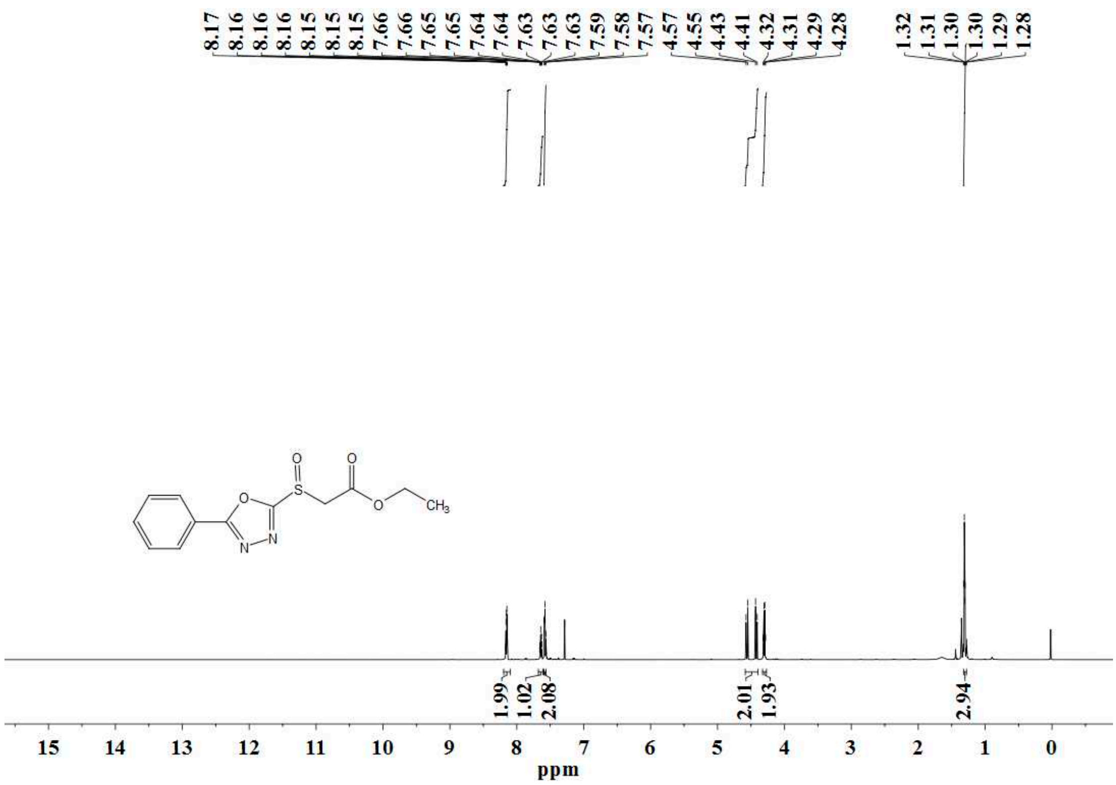

Figure S30-1. ^{1}H NMR spectrum of compound **5o**.

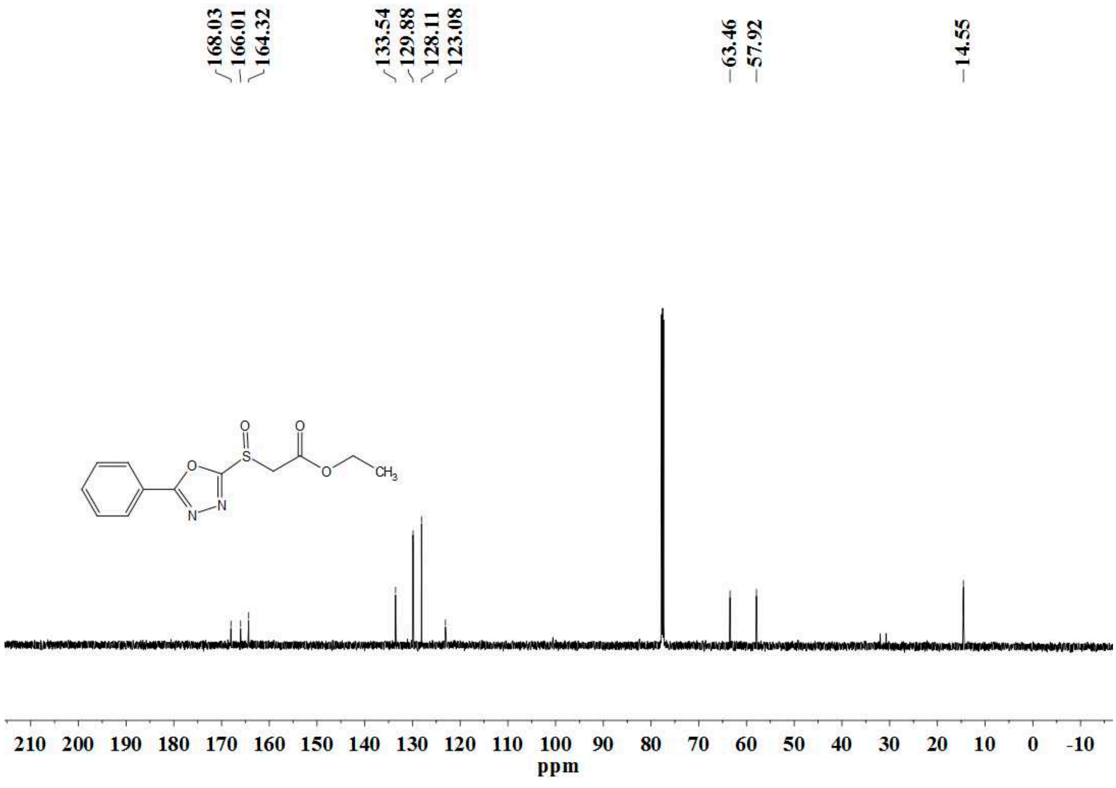

Figure S30-2. ^{13}C NMR spectrum of compound **5o**.

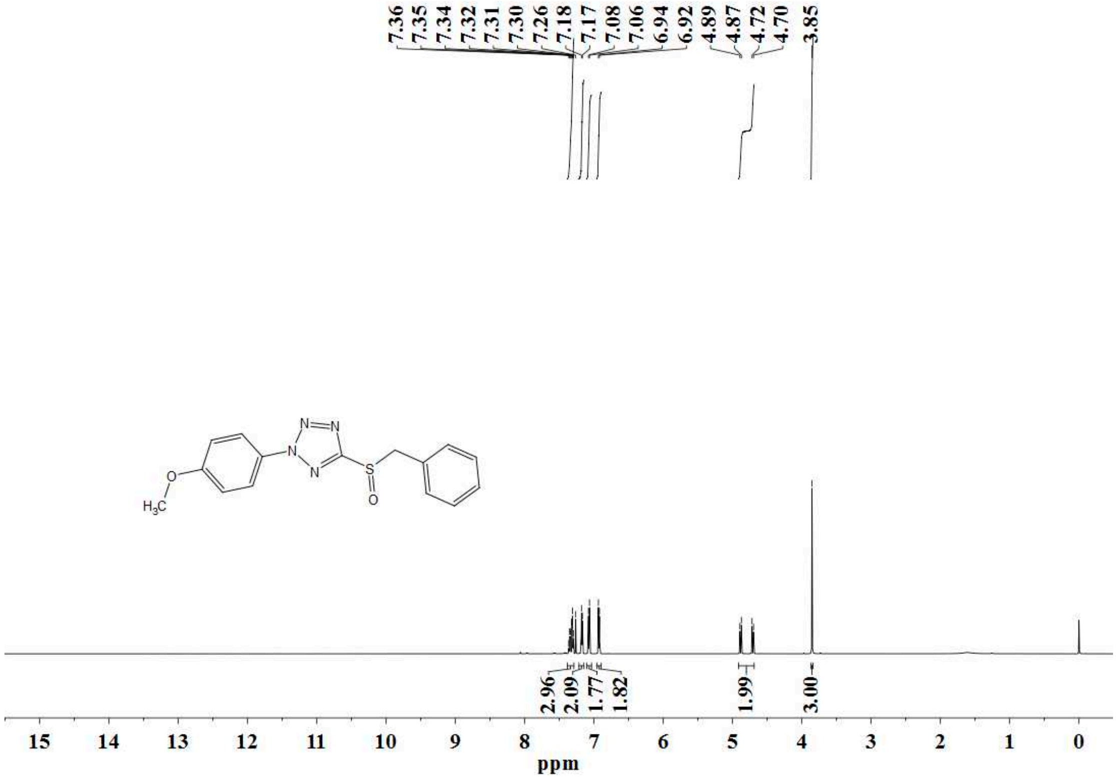

Figure S31-1. ^{1}H NMR spectrum of compound **6a**.

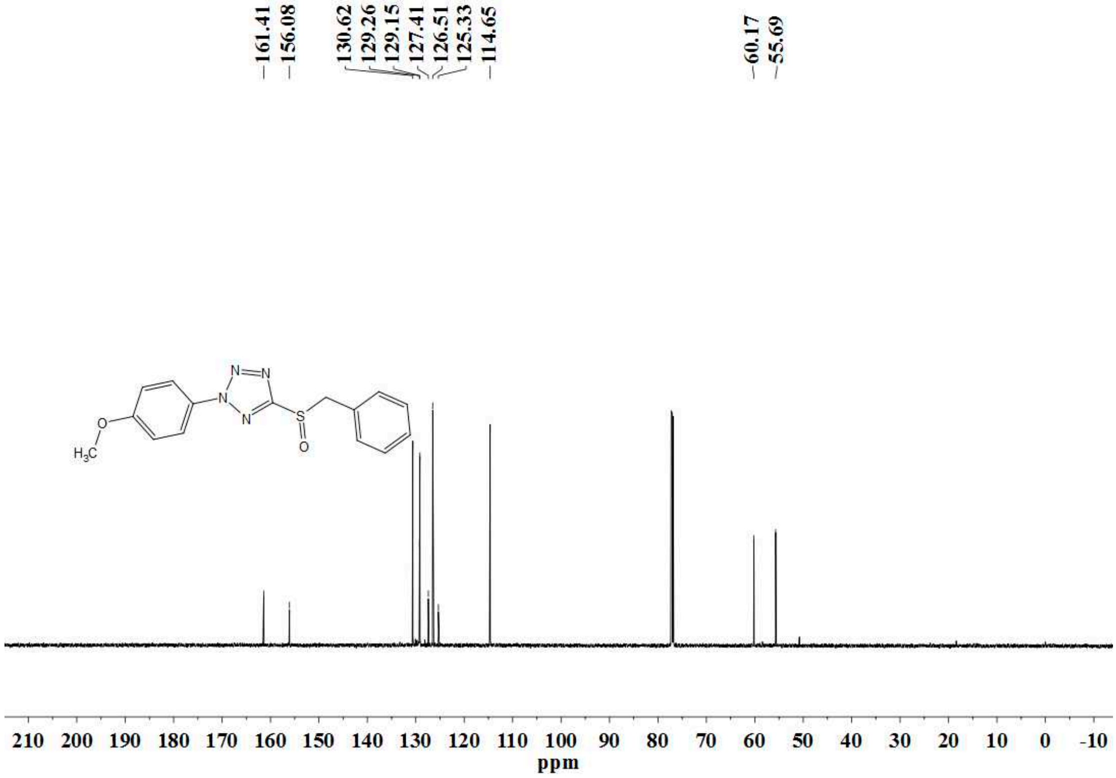

Figure S31-2. ^{13}C NMR spectrum of compound **6a**.

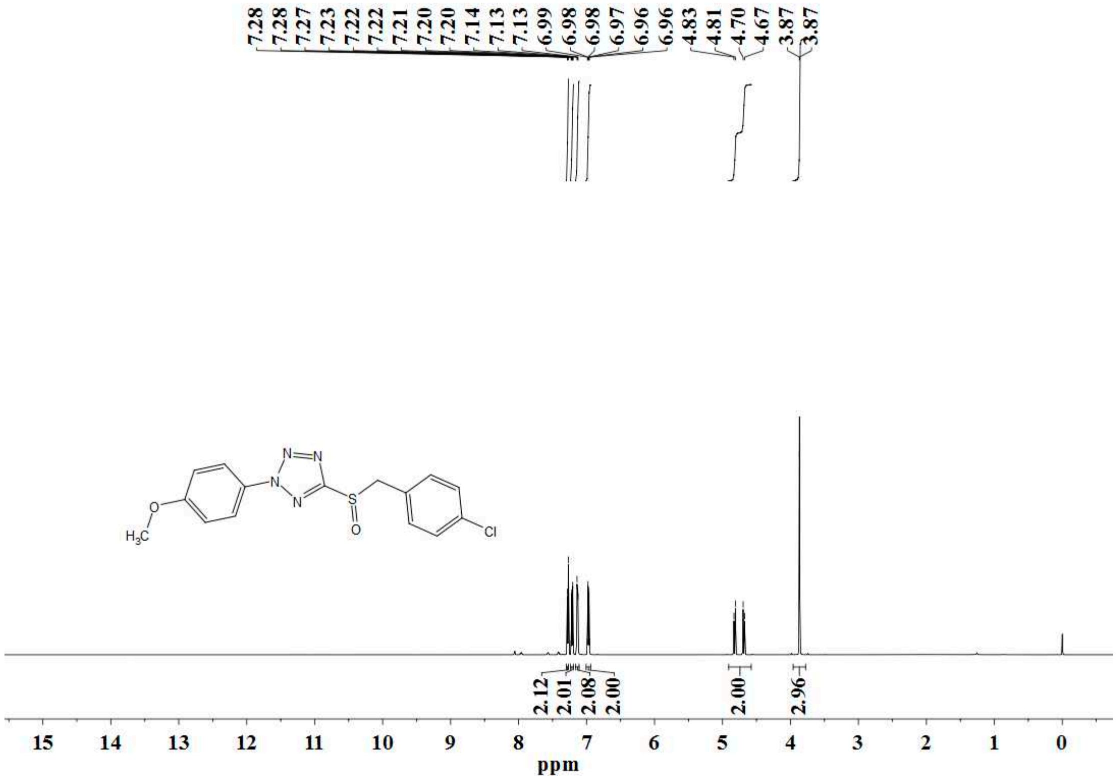

Figure S32-1. ^{1}H NMR spectrum of compound **6b**.

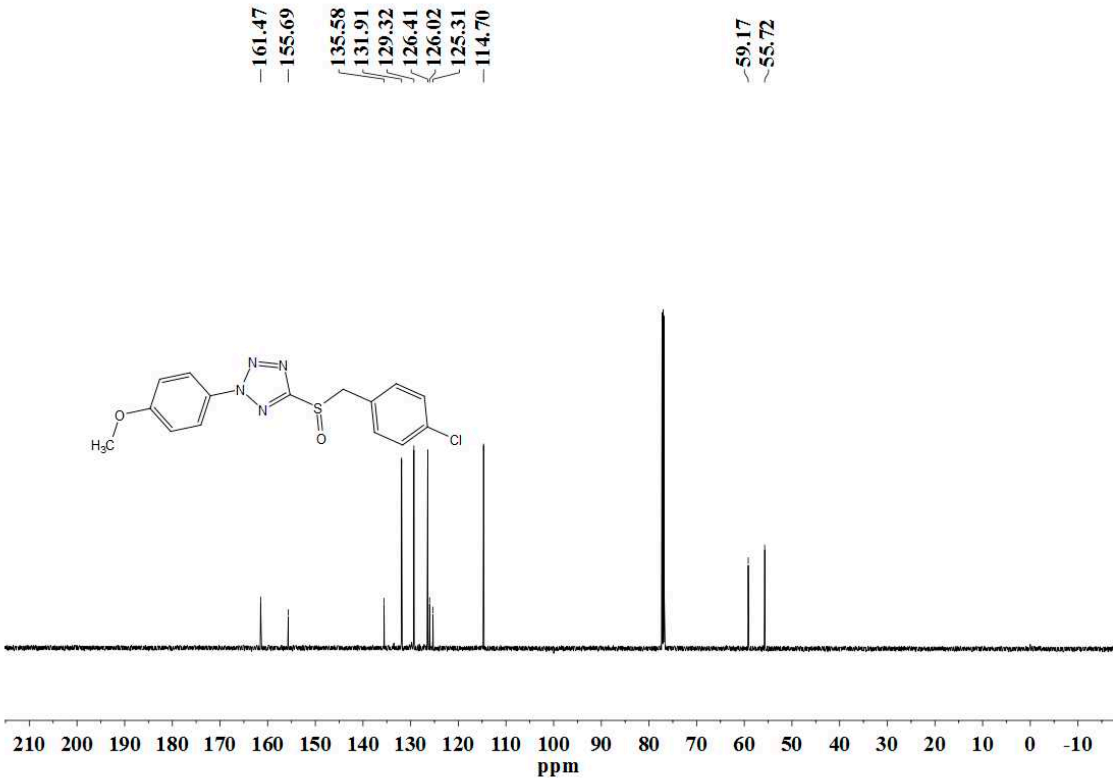

Figure S32-2. ^{13}C NMR spectrum of compound **6b**.

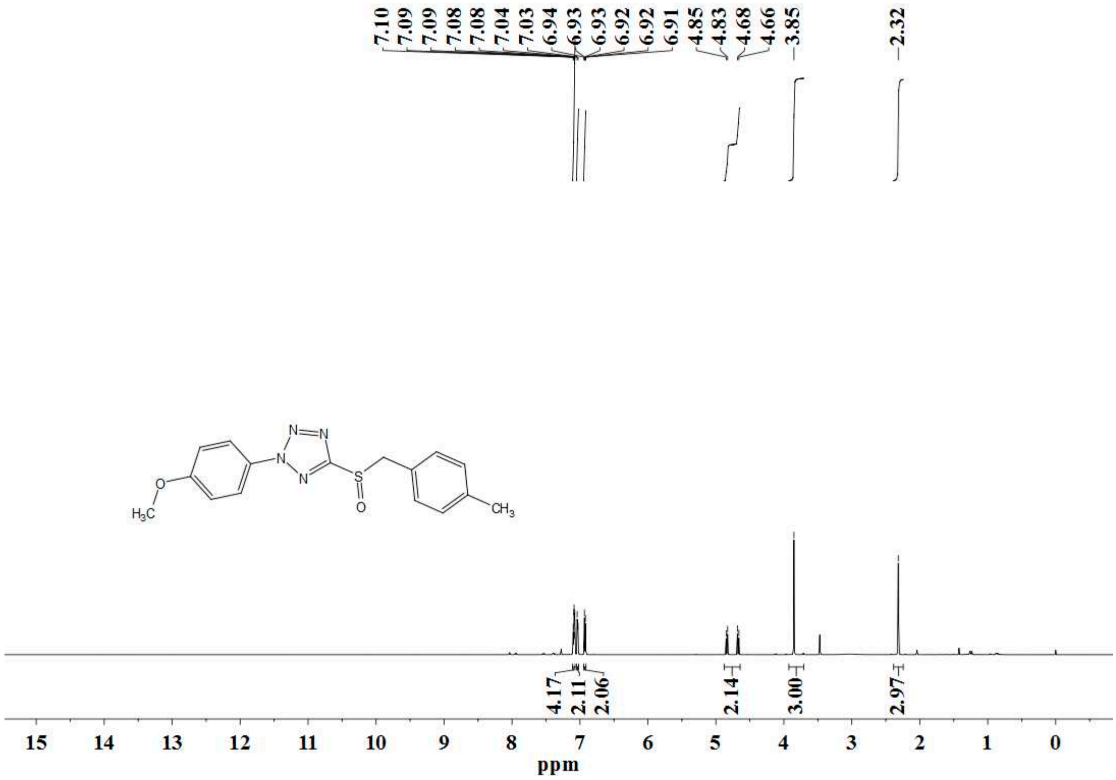

Figure S33-1. ^{1}H NMR spectrum of compound **6e**.

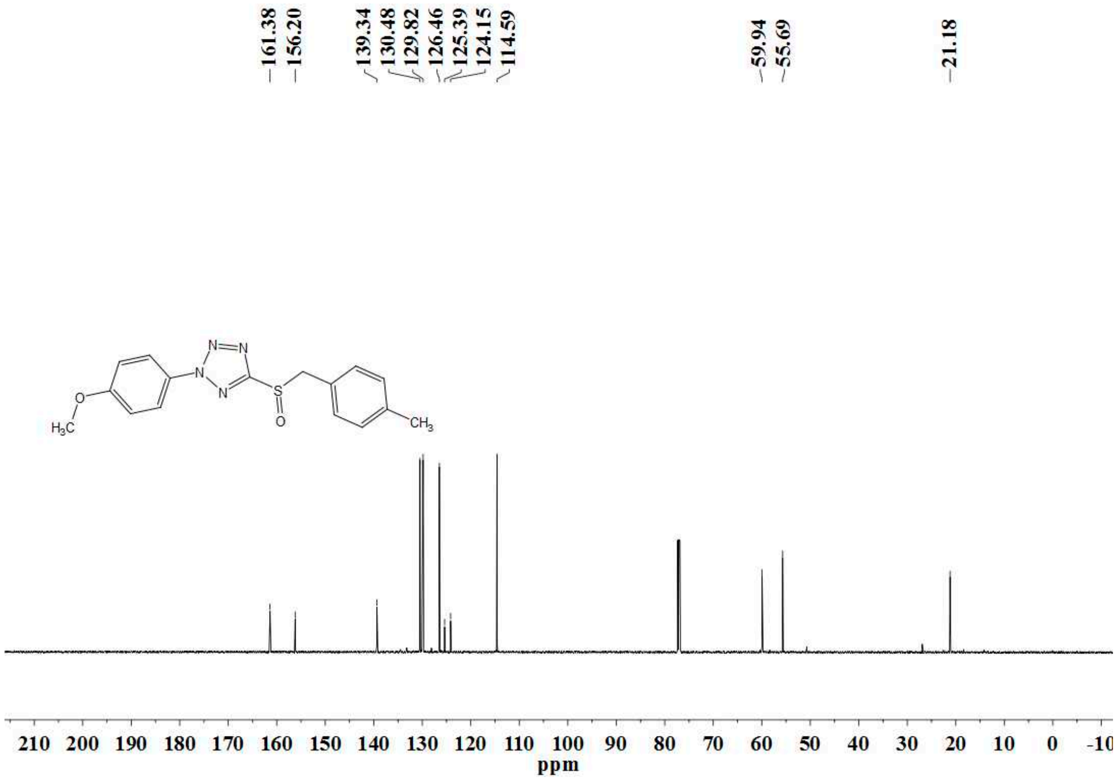

Figure S33-2. ^{13}C NMR spectrum of compound **6e**.

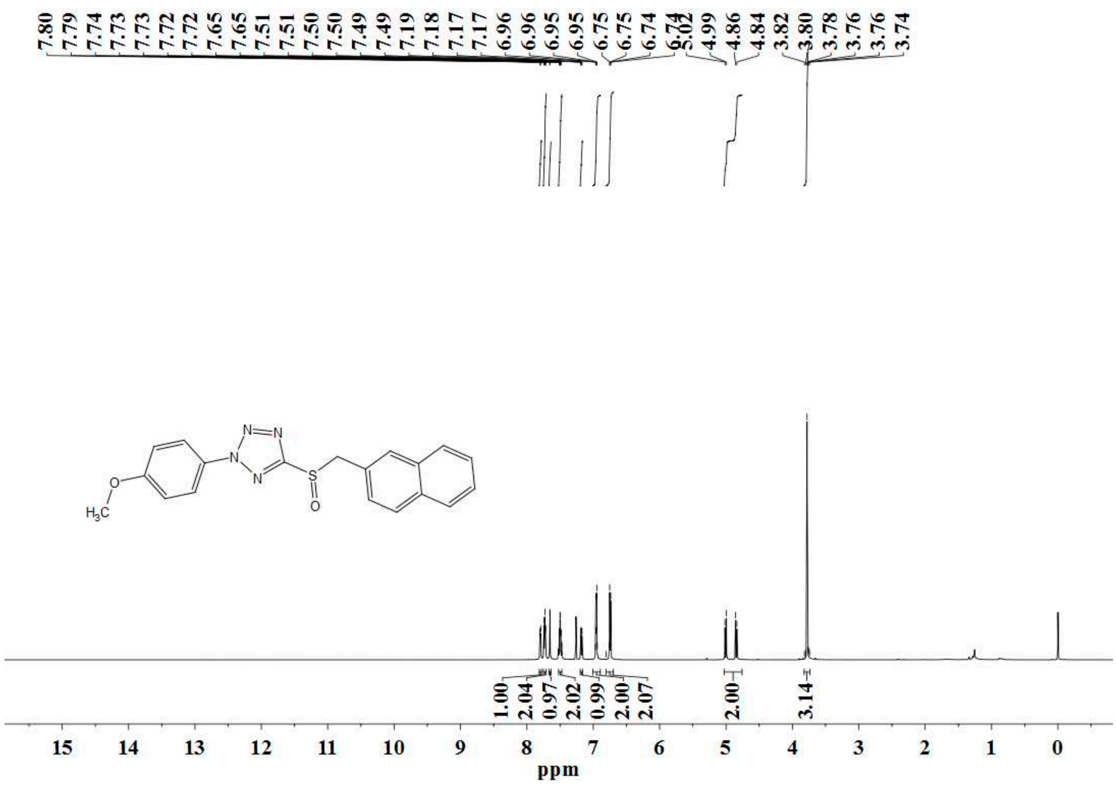

Figure S34-1. ^{1}H NMR spectrum of compound **6f**.

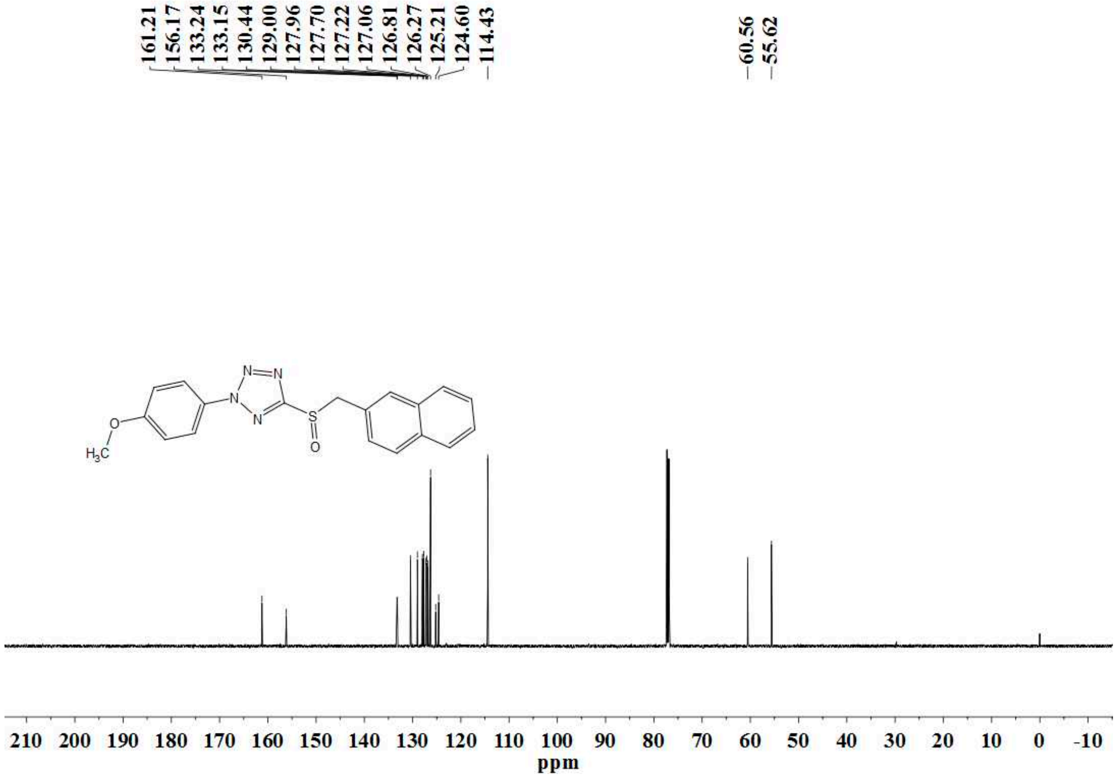

Figure S34-2. ^{13}C NMR spectrum of compound **6f**.

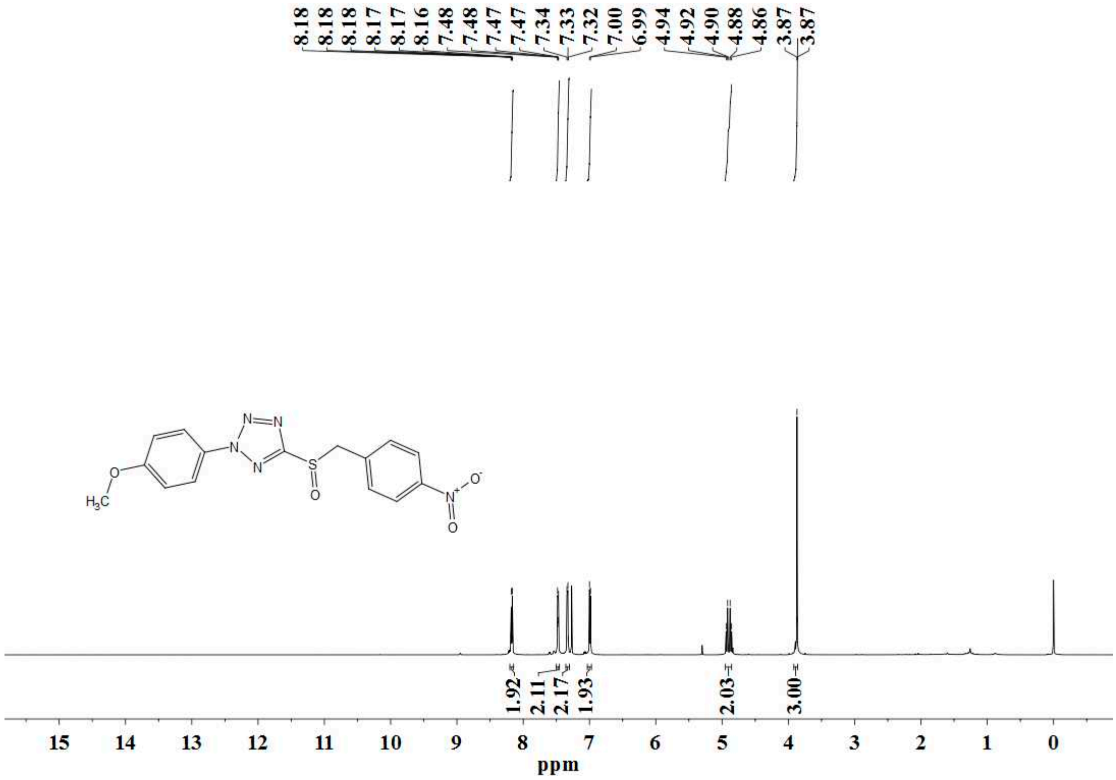

Figure S35-1. ^{1}H NMR spectrum of compound **6g**.

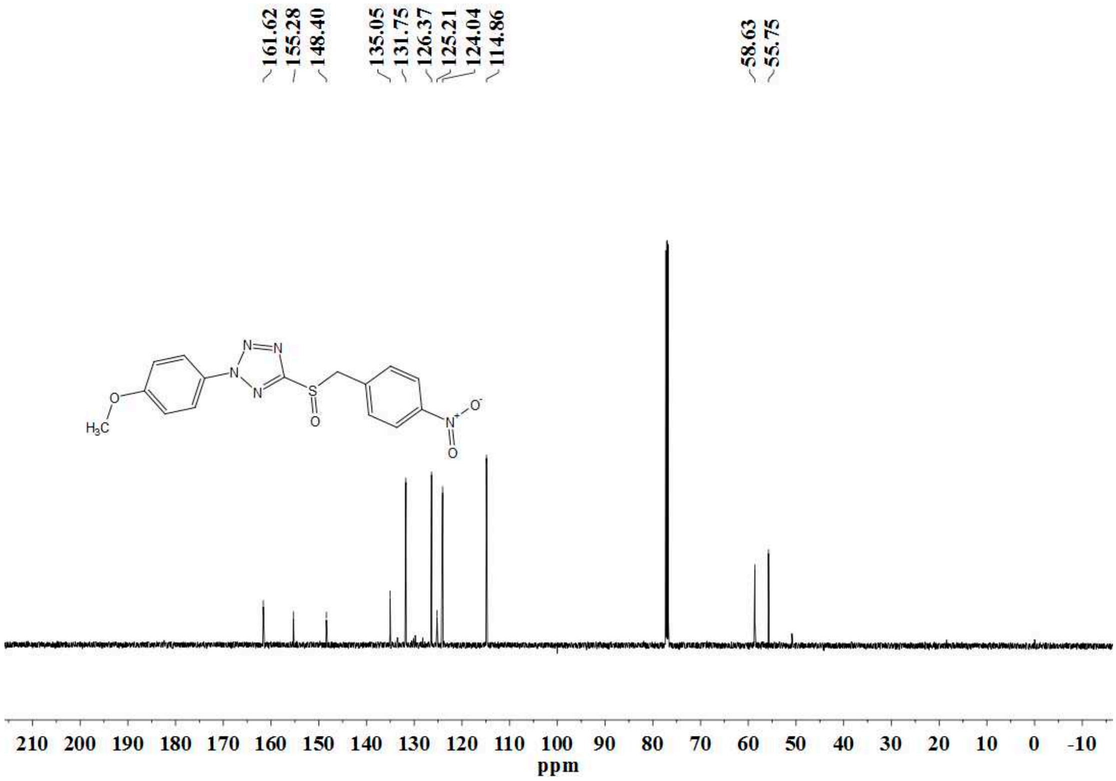

Figure S35-2. ^{13}C NMR spectrum of compound **6g**.

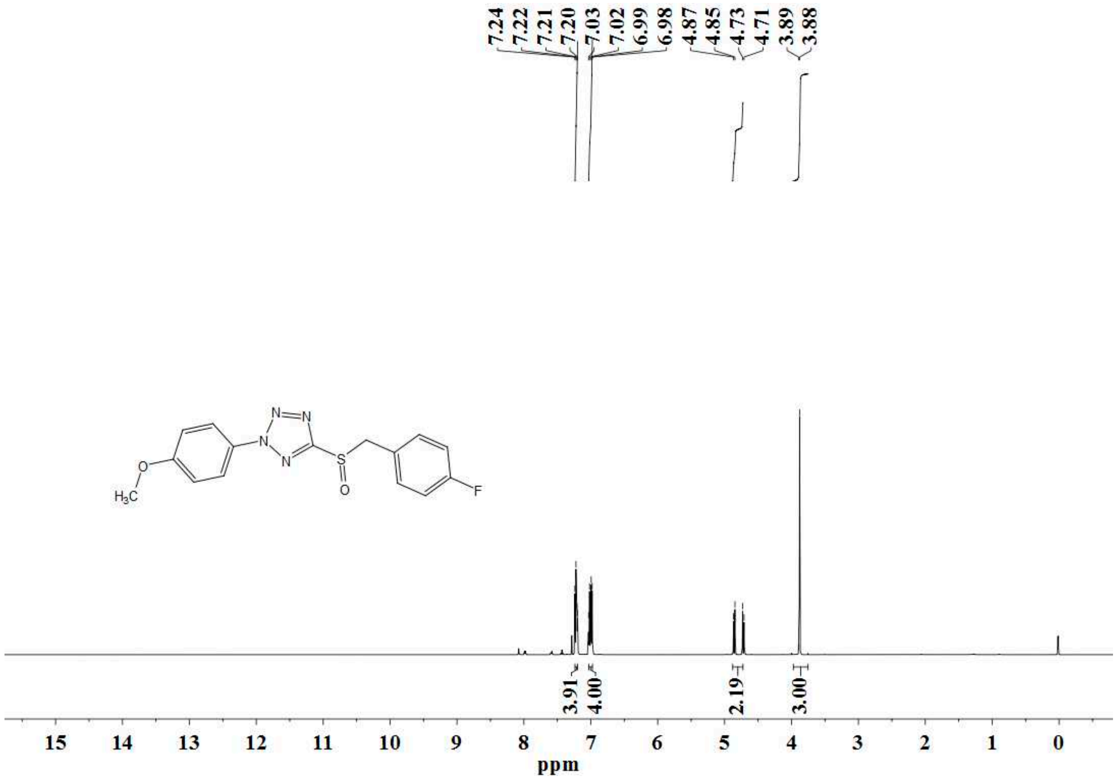

Figure S36-1. ^{1}H NMR spectrum of compound **6h**.

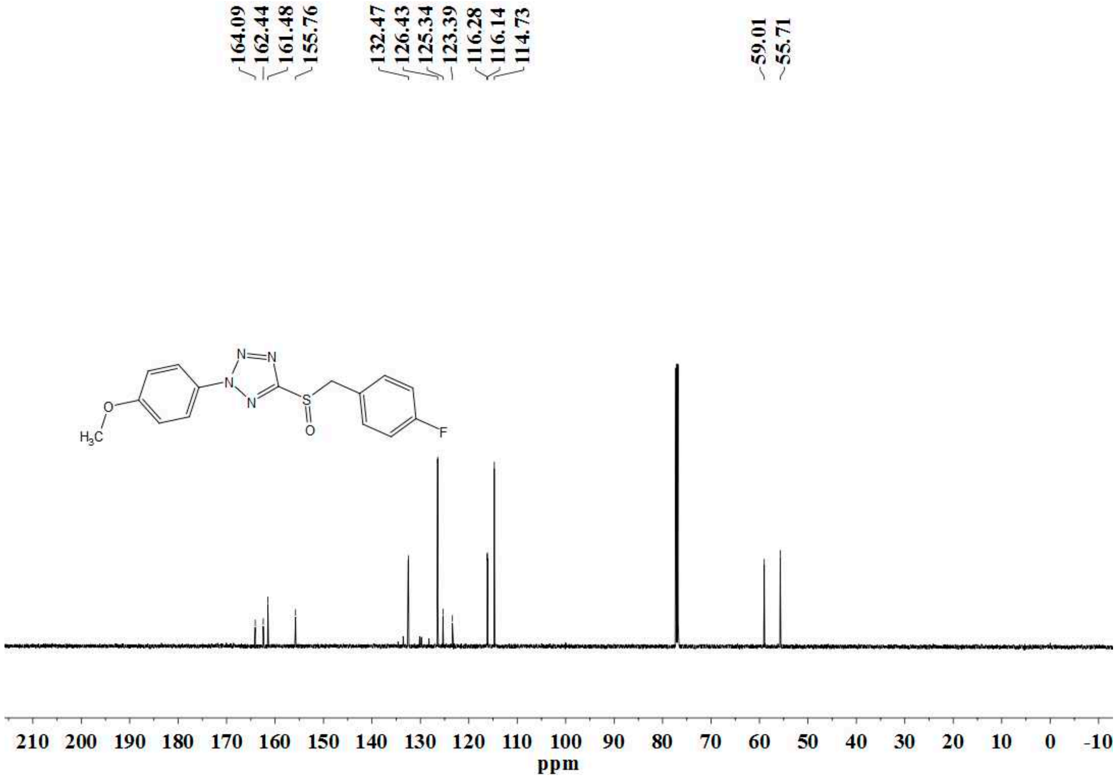

Figure S36-2. ^{13}C NMR spectrum of compound **6h**.

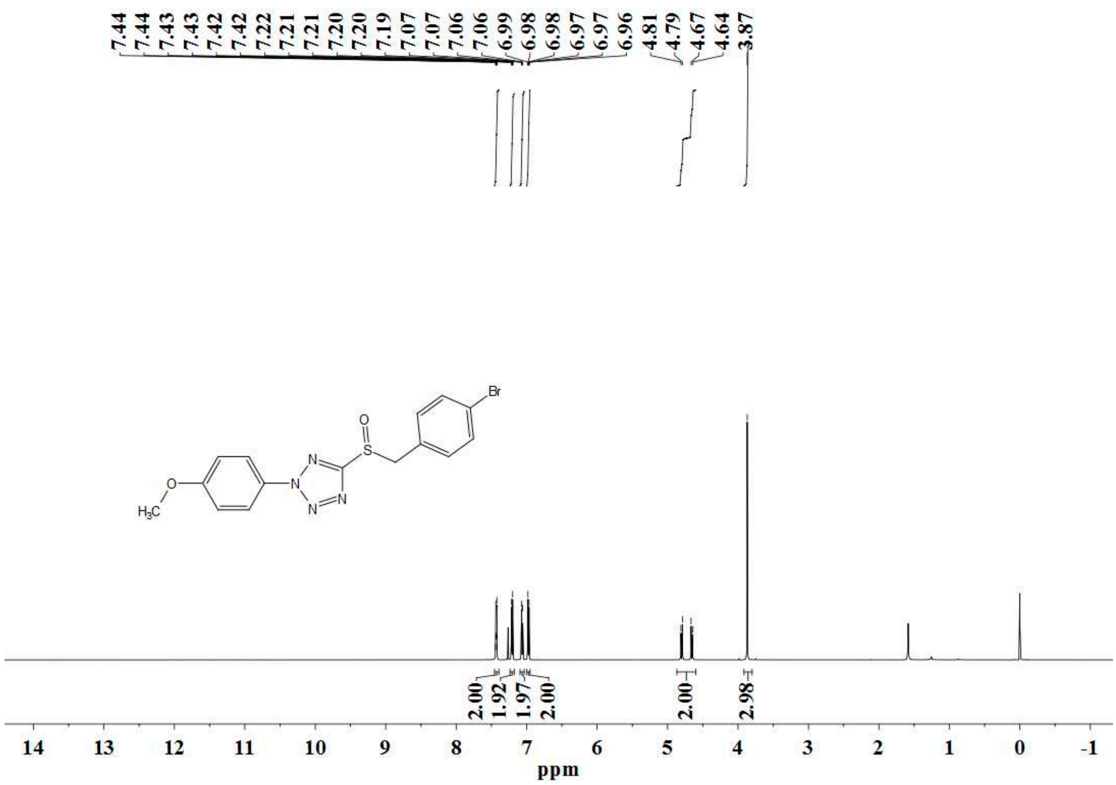

Figure S37-1. ^{1}H NMR spectrum of compound **6i**.

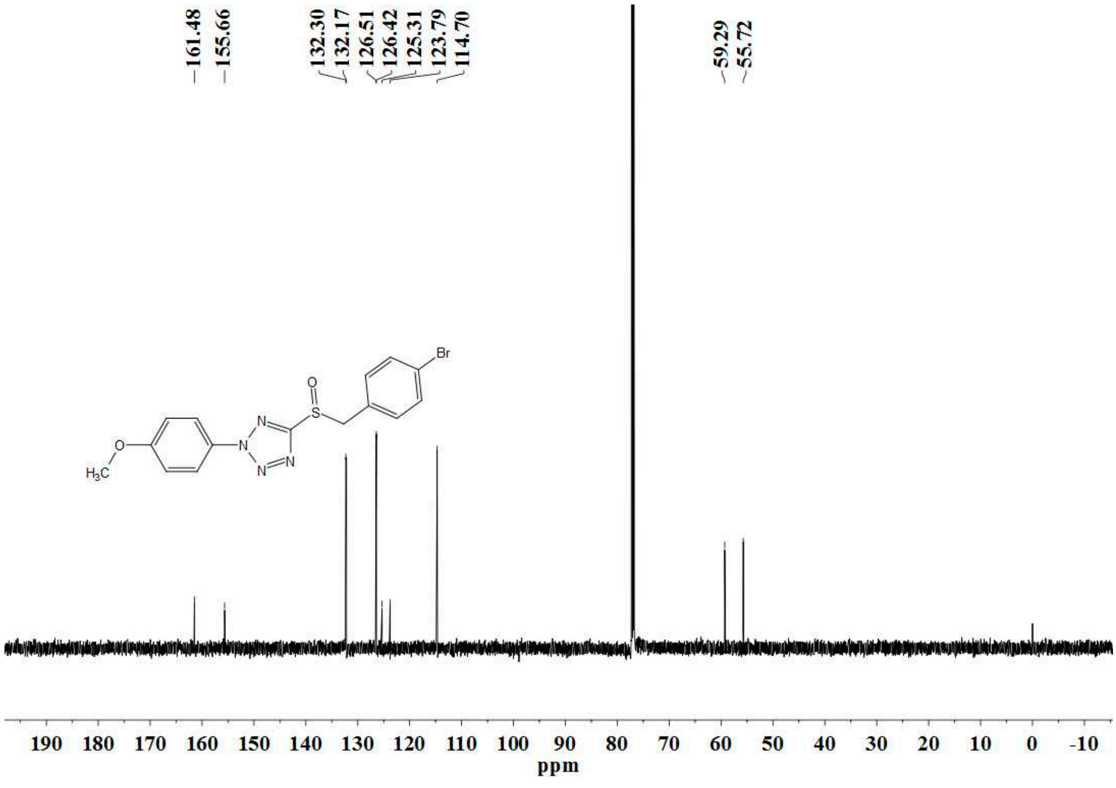

Figure S37-2. ^{13}C NMR spectrum of compound **6i**.

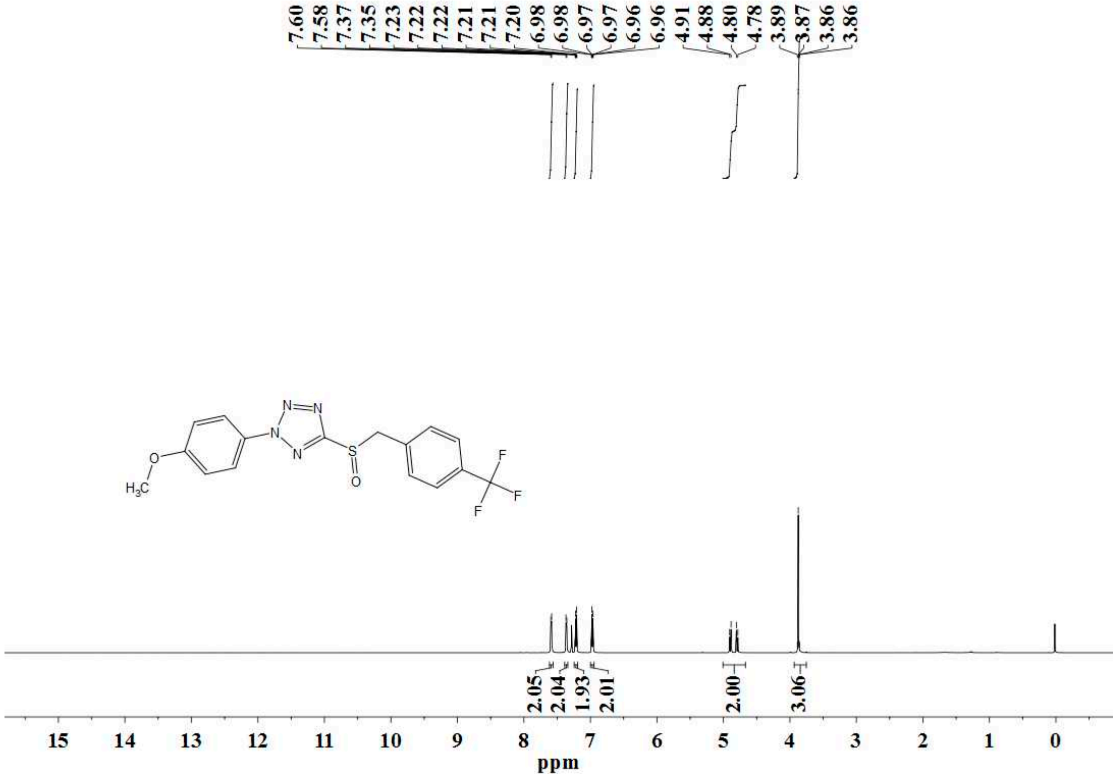

Figure S38-1. ^{1}H NMR spectrum of compound **6j**.

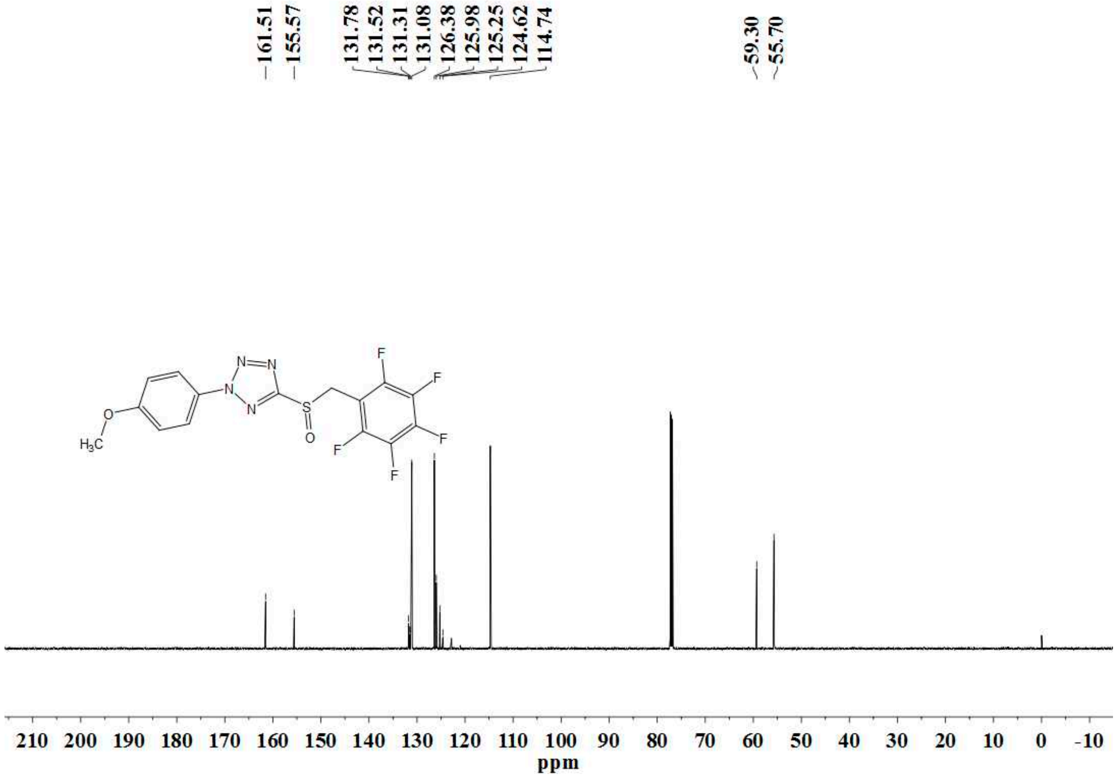

Figure S38-2. ^{13}C NMR spectrum of compound **6j**.

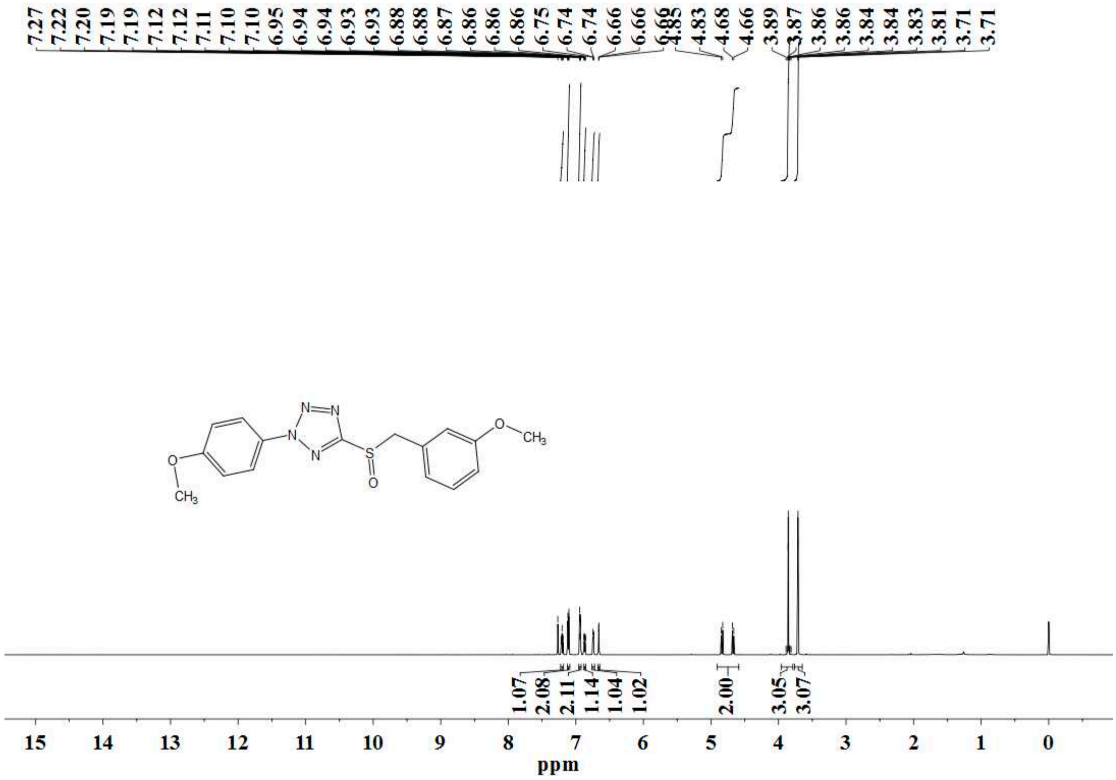

Figure S39-1. ^{1}H NMR spectrum of compound **6k**.

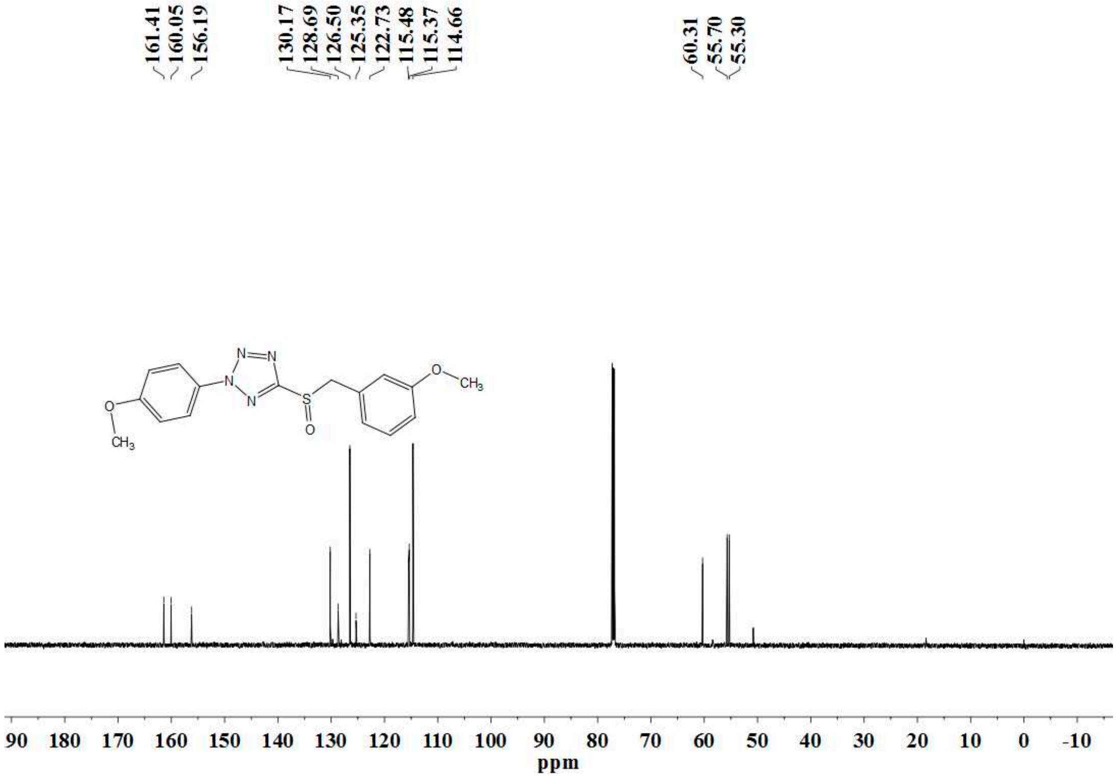

Figure S39-2. ^{13}C NMR spectrum of compound **6k**.

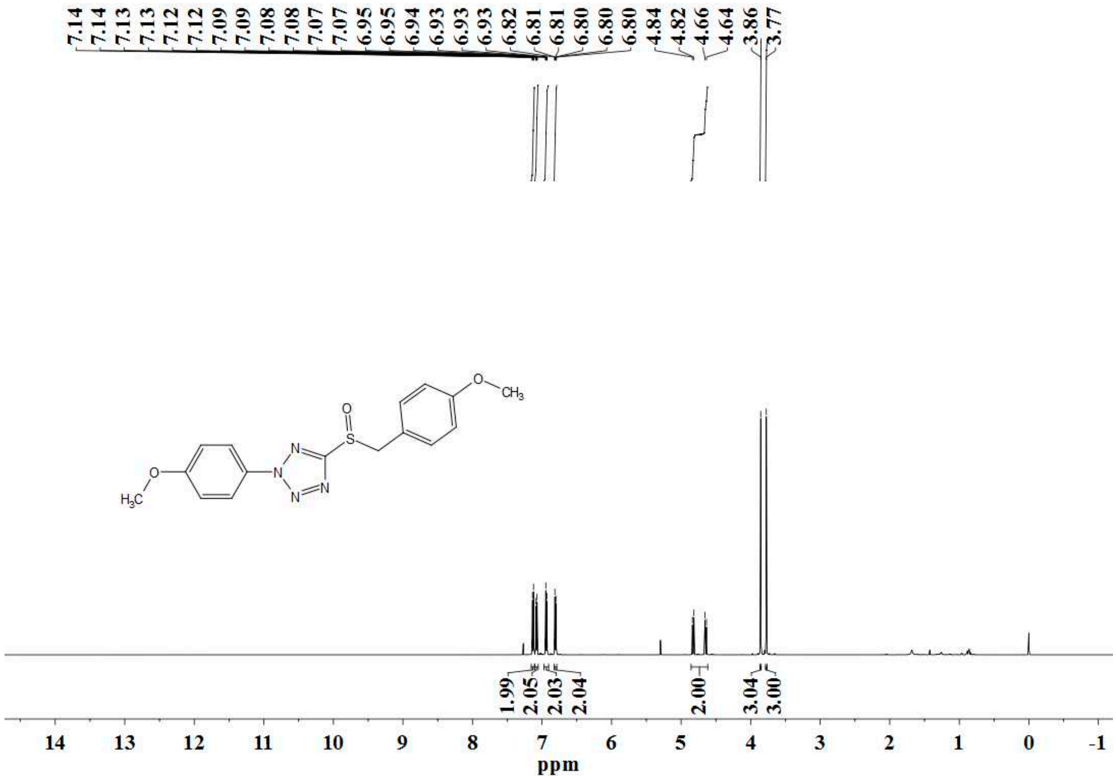

Figure S40-1. ^{1}H NMR spectrum of compound **6l**.

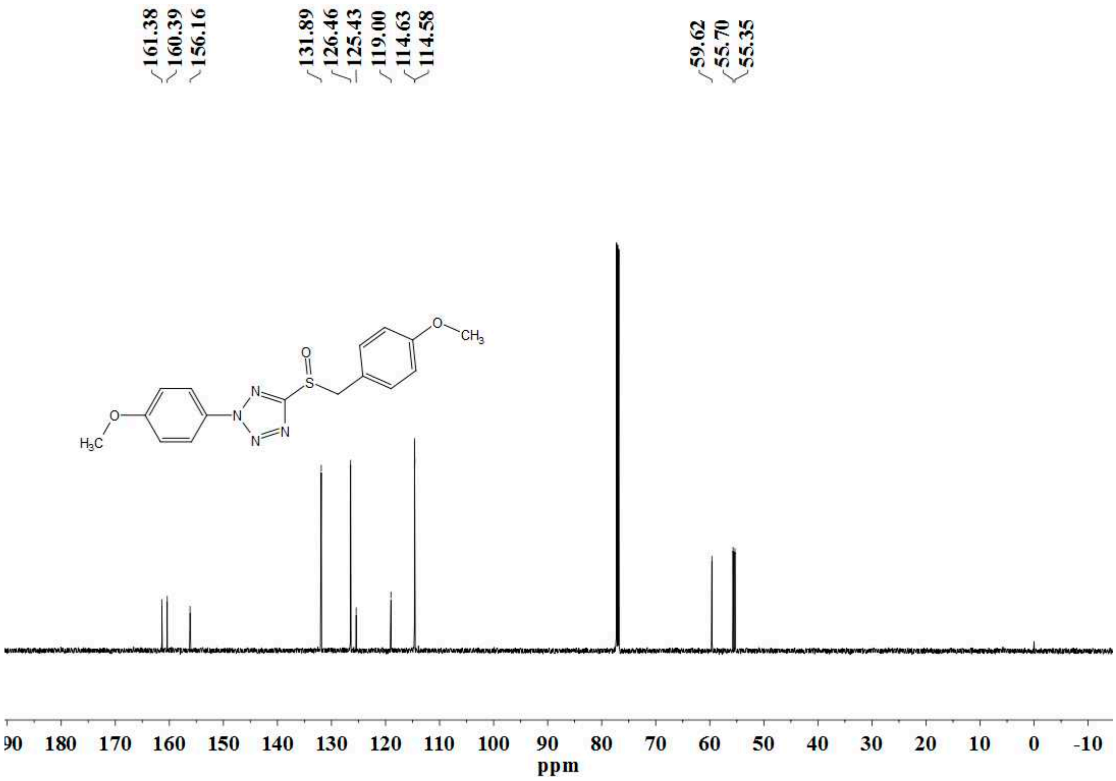

Figure S40-2. ^{13}C NMR spectrum of compound **6l**.

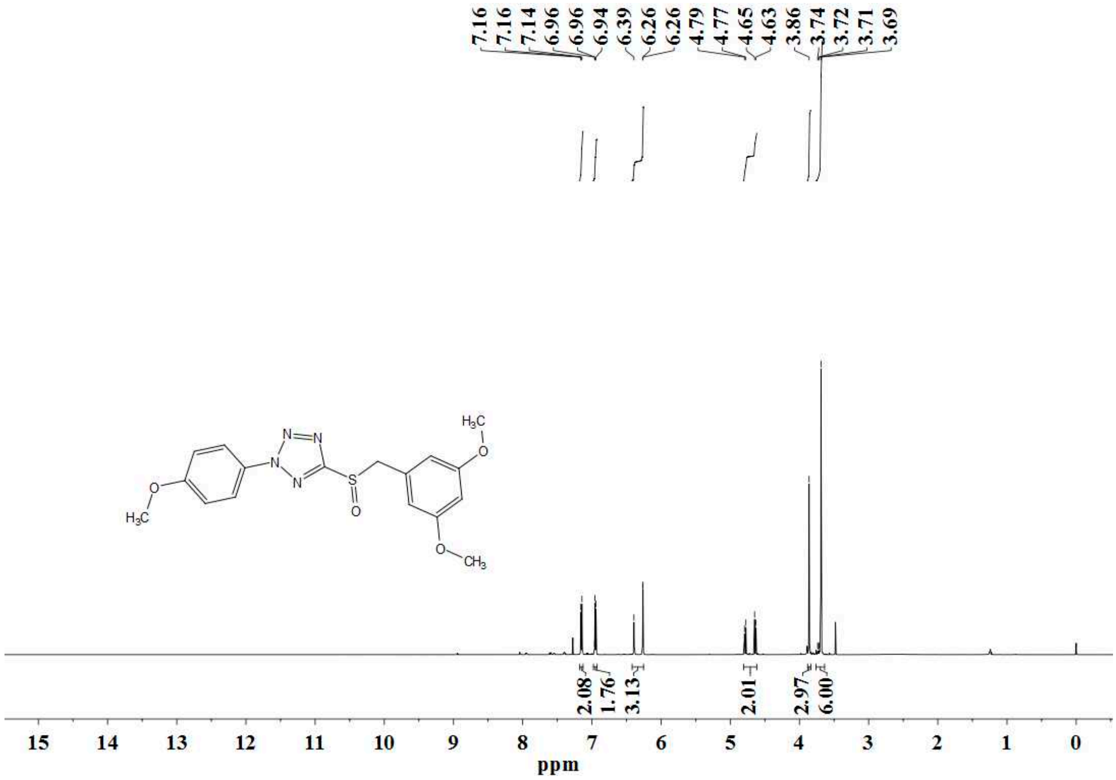

Figure S41-1. ^{1}H NMR spectrum of compound **6m**.

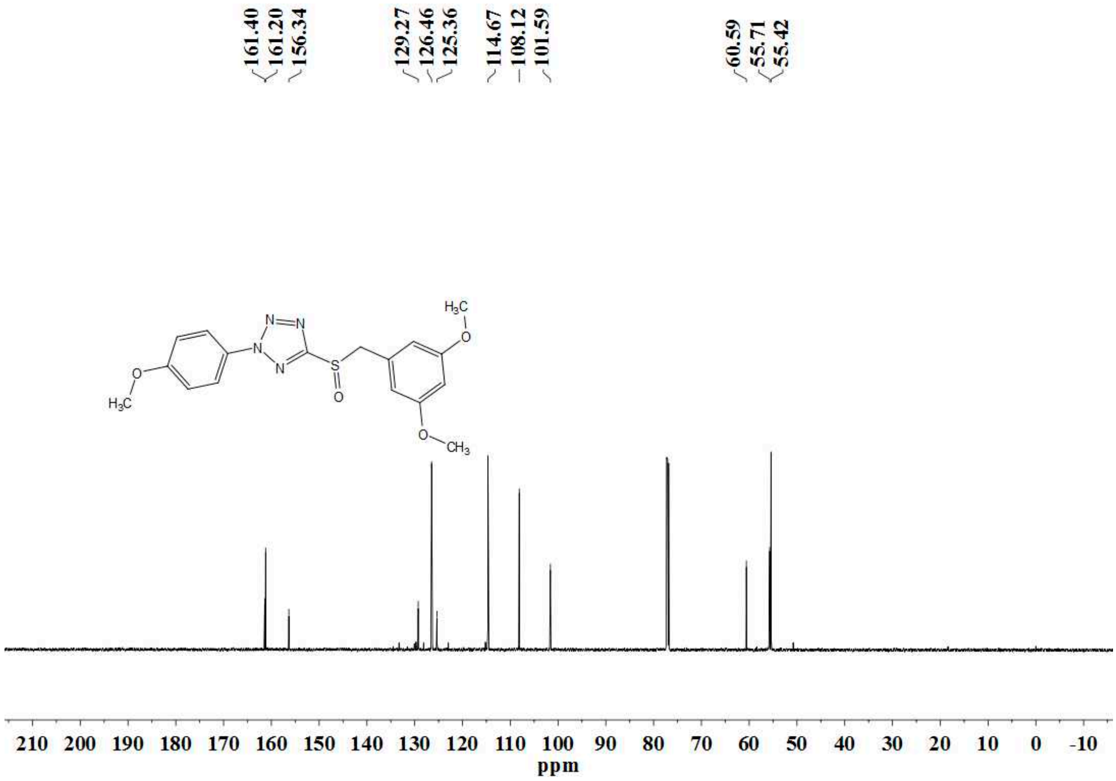

Figure S41-2. ^{13}C NMR spectrum of compound **6m**.

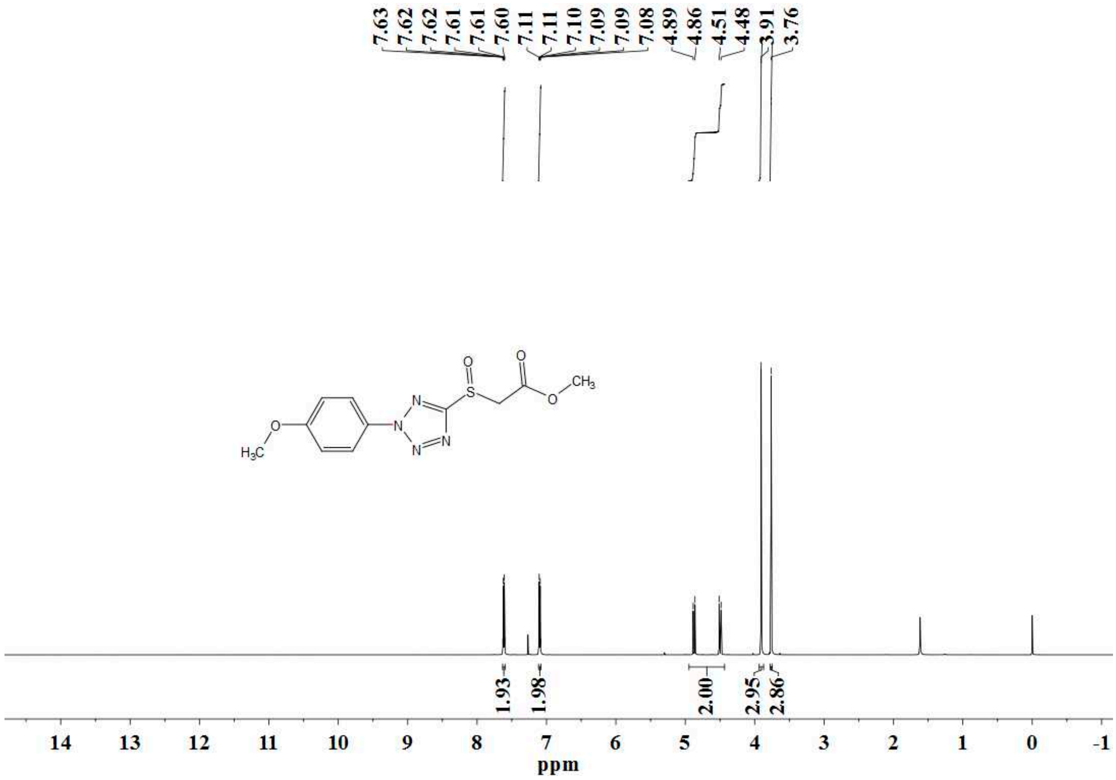

Figure S42-1. ^{1}H NMR spectrum of compound **6n**.

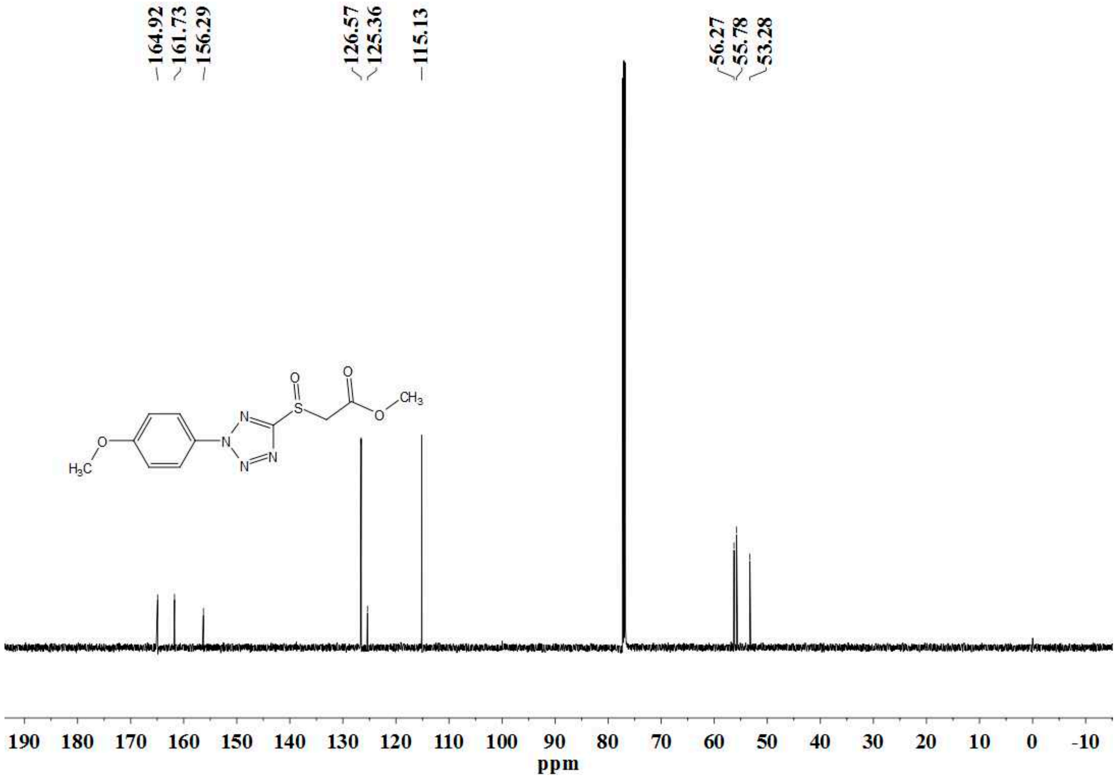

Figure S42-2. ^{13}C NMR spectrum of compound **6n**.

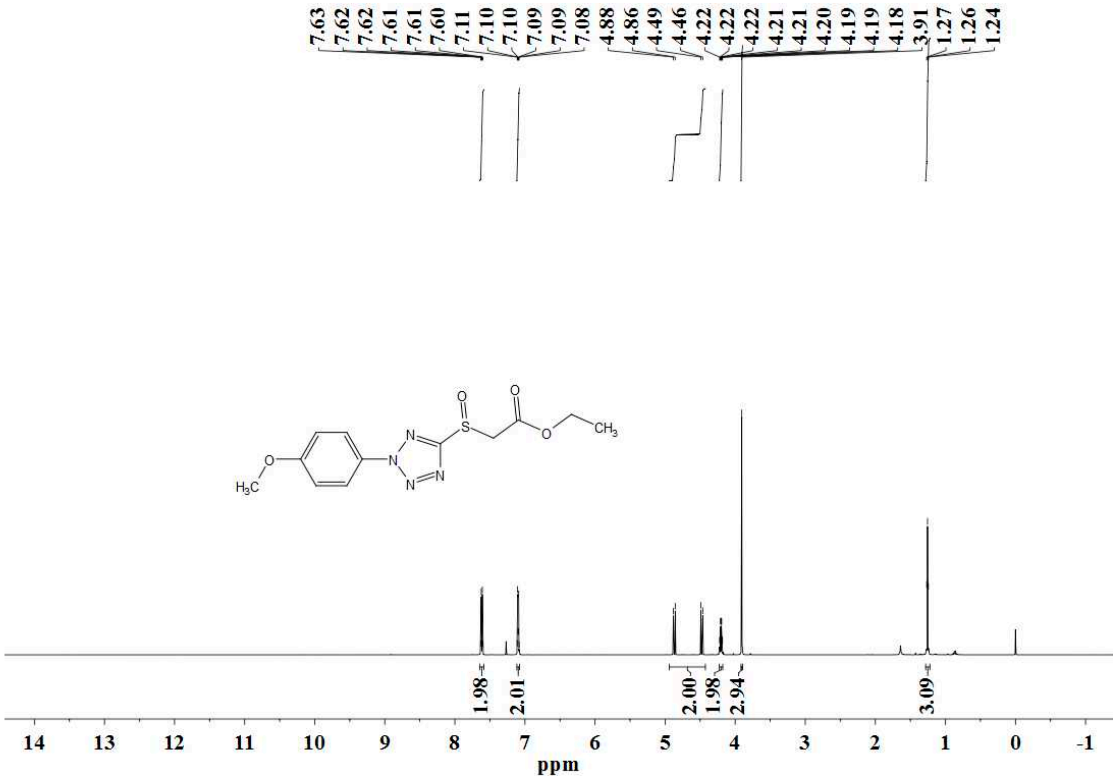

Figure S43-1. ^{1}H NMR spectrum of compound **6o**.

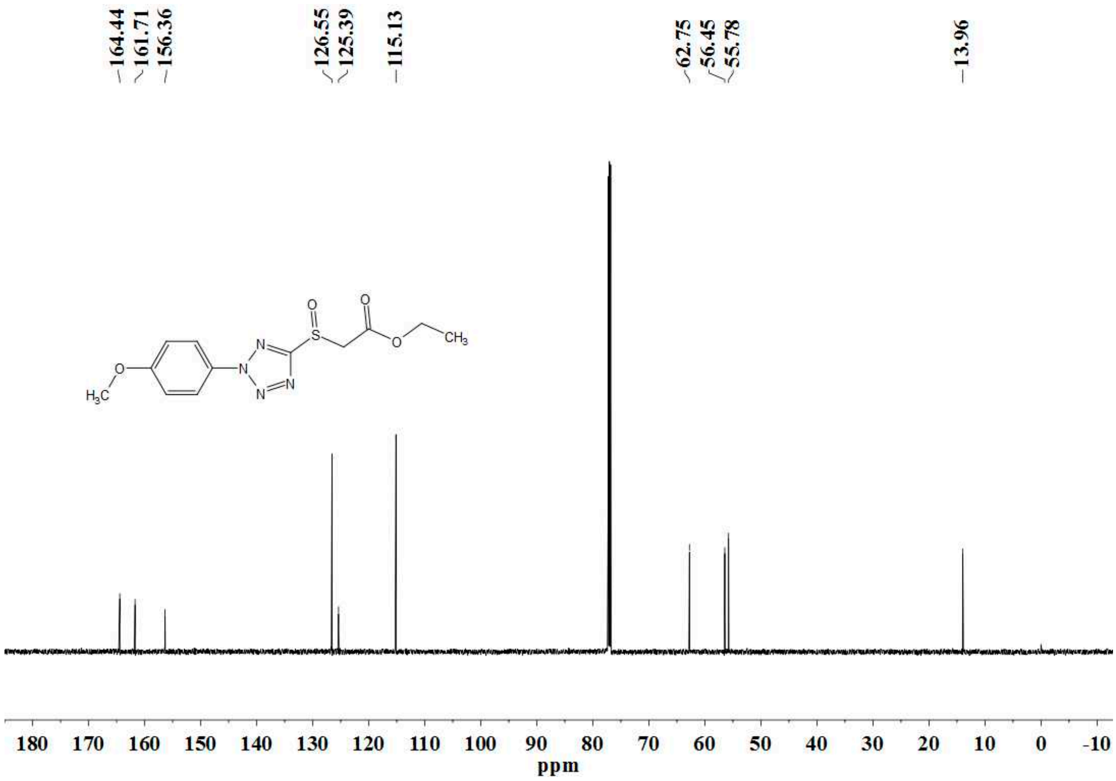

Figure S43-2. ^{13}C NMR spectrum of compound **6o**.

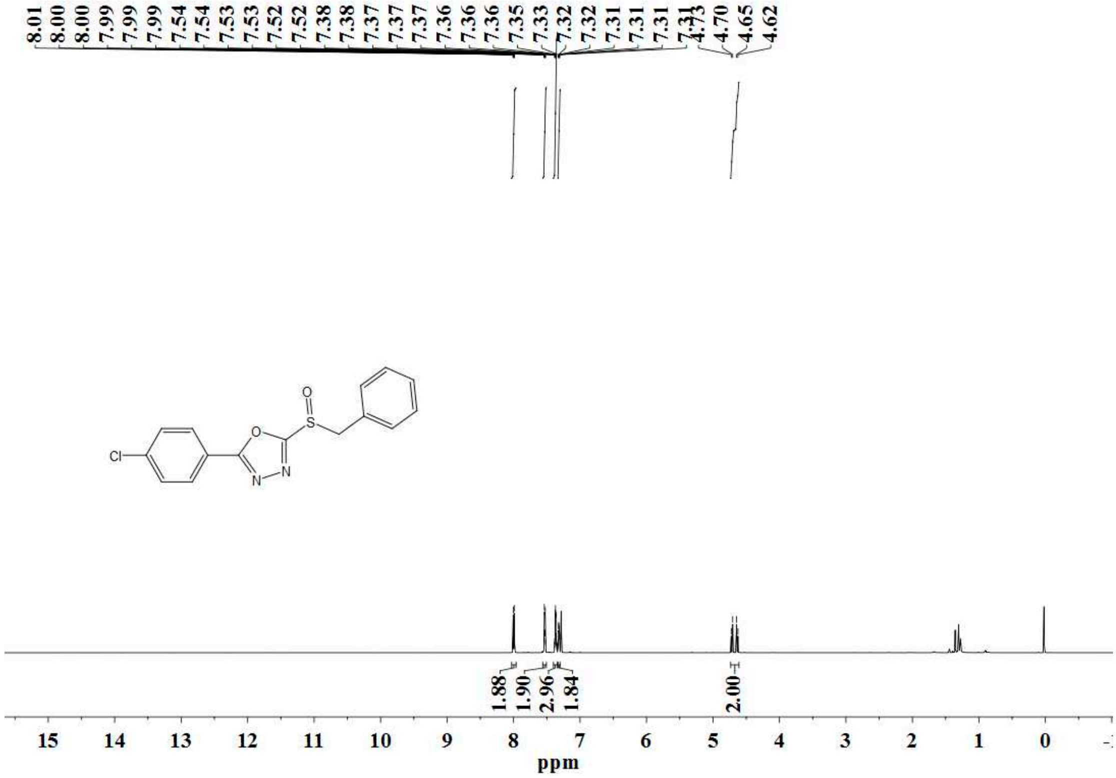

Figure S44-1. ^{1}H NMR spectrum of compound **7a**.

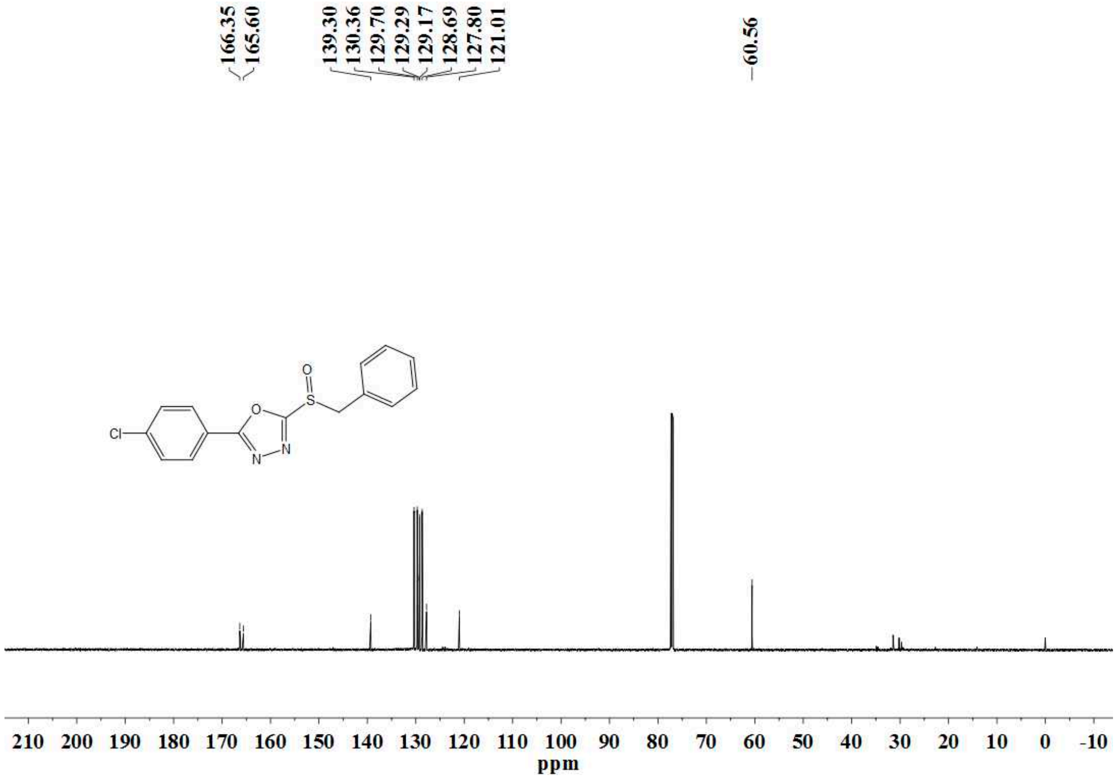

Figure S44-2. ^{13}C NMR spectrum of compound **7a**.

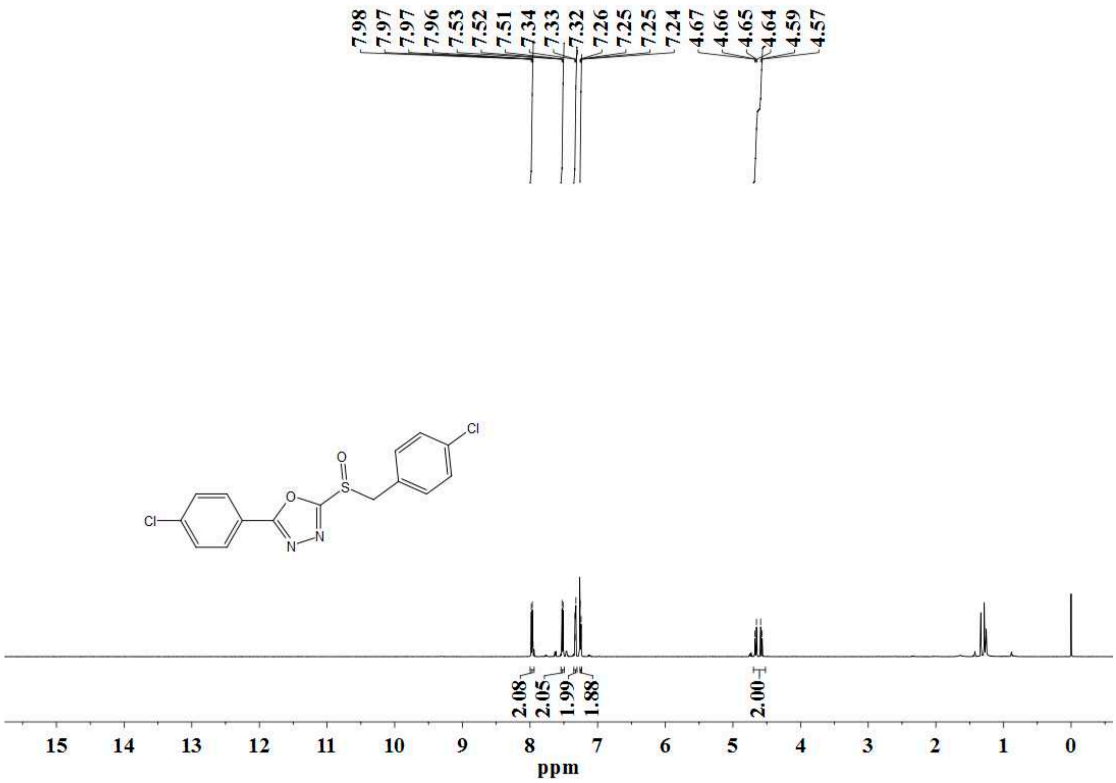

Figure S45-1. ^{1}H NMR spectrum of compound **7b**.

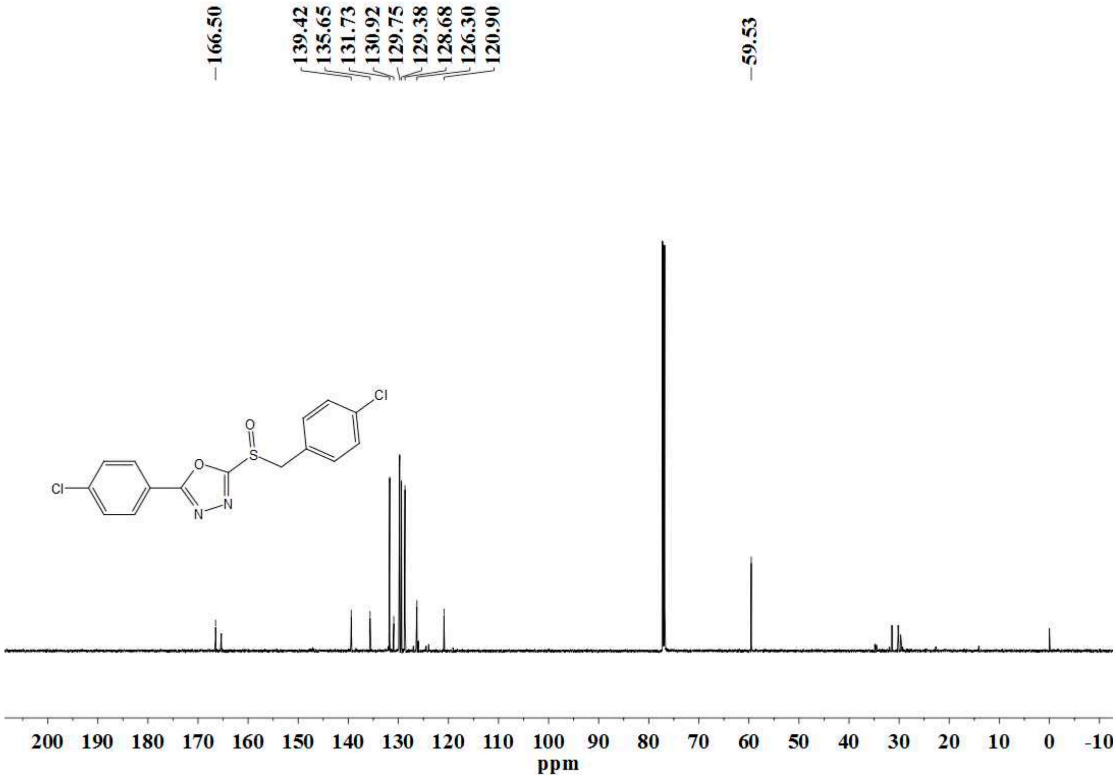

Figure S45-2. ^{13}C NMR spectrum of compound **7b**.

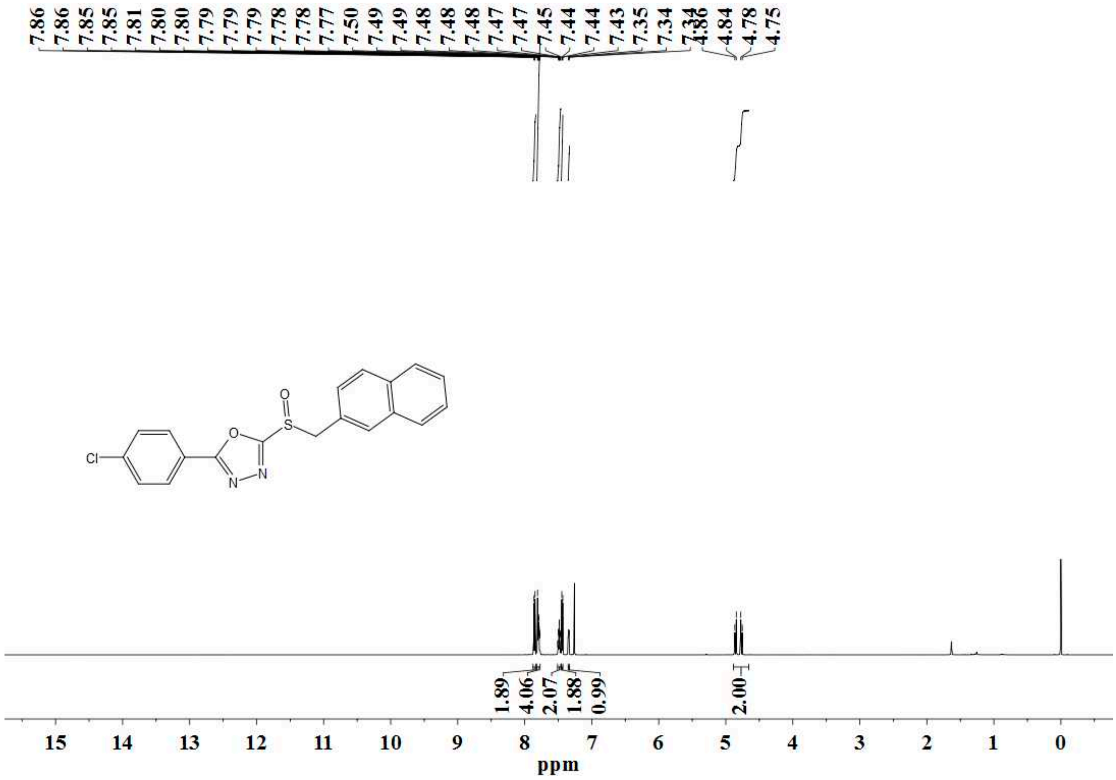

Figure S46-1. ^{1}H NMR spectrum of compound **7f**.

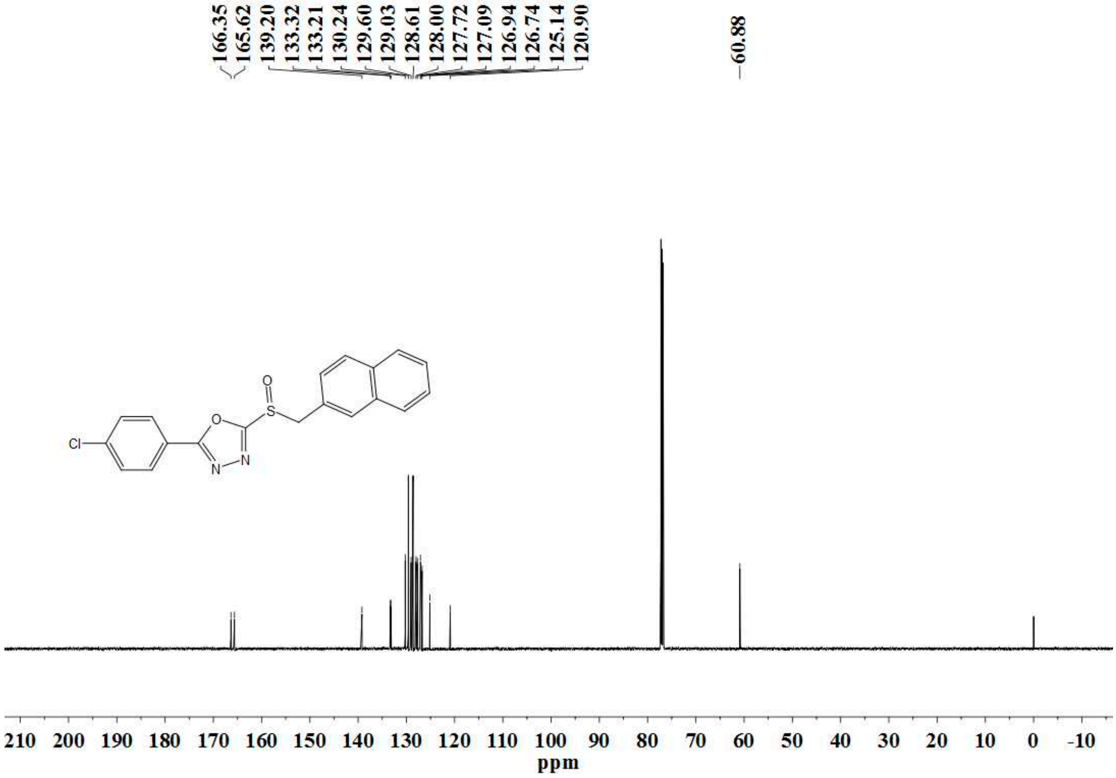

Figure S46-2. ^{13}C NMR spectrum of compound **7f**.

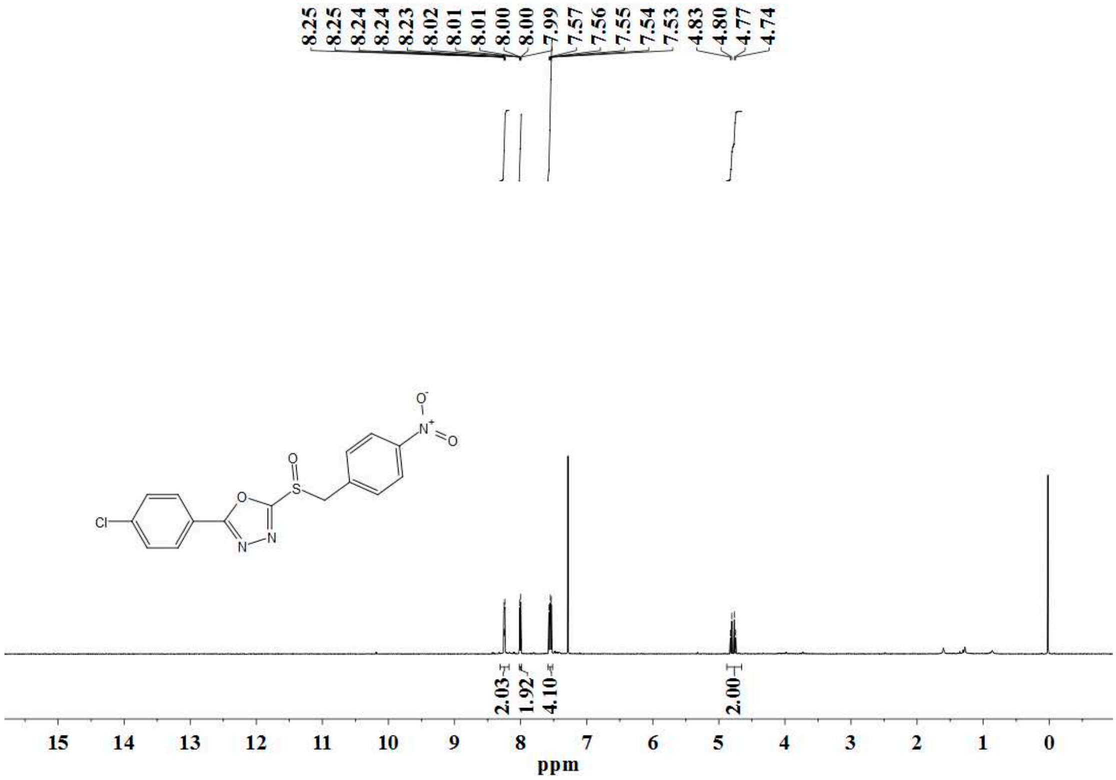

Figure S47-1. ^{1}H NMR spectrum of compound **7g**.

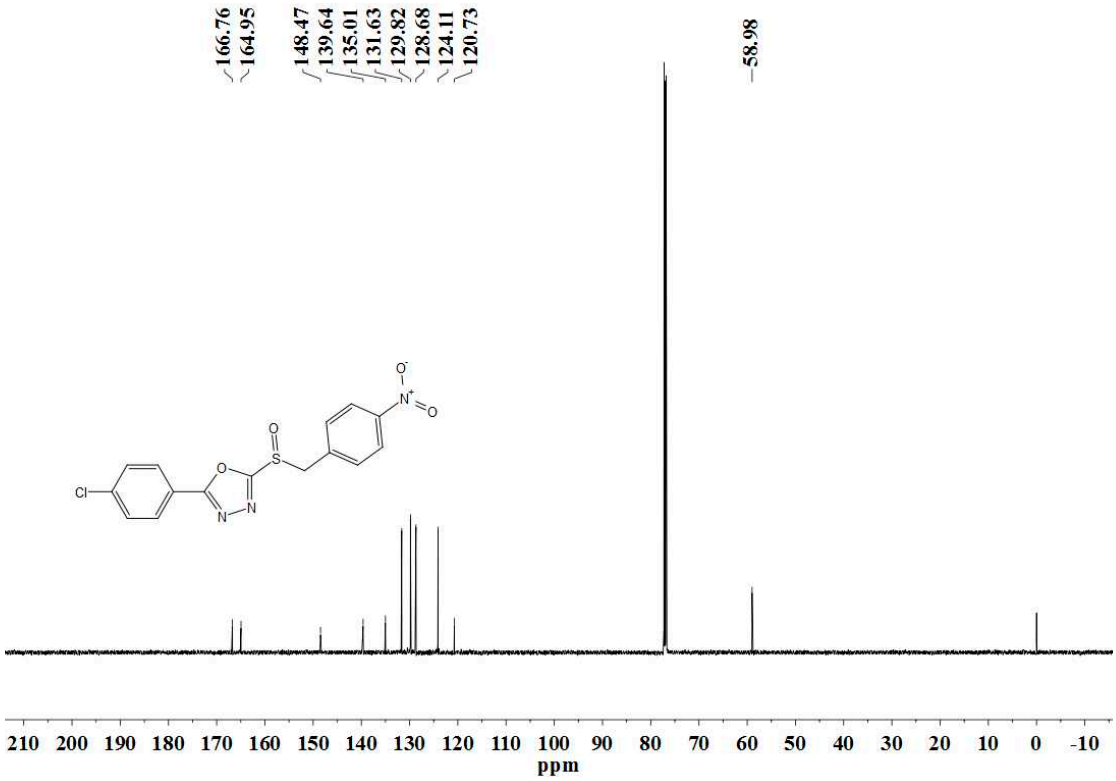

Figure S47-2. ^{13}C NMR spectrum of compound **7g**.

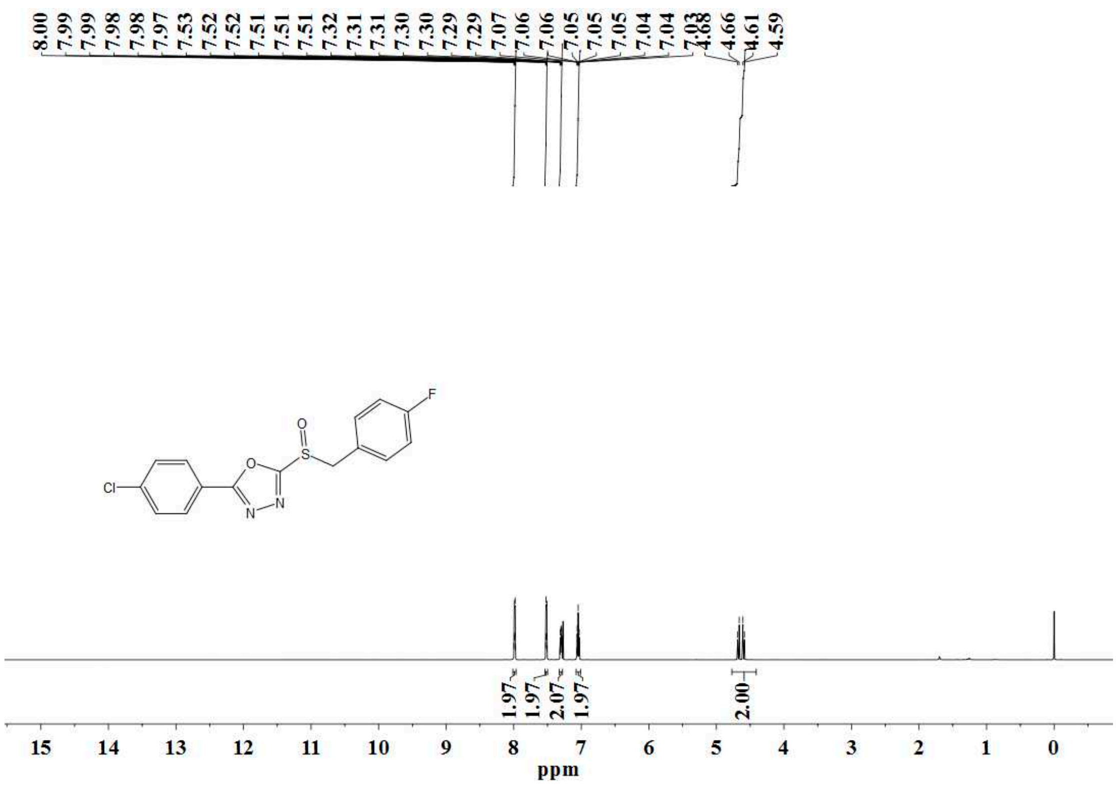

Figure S48-1. ^{1}H NMR spectrum of compound **7h**.

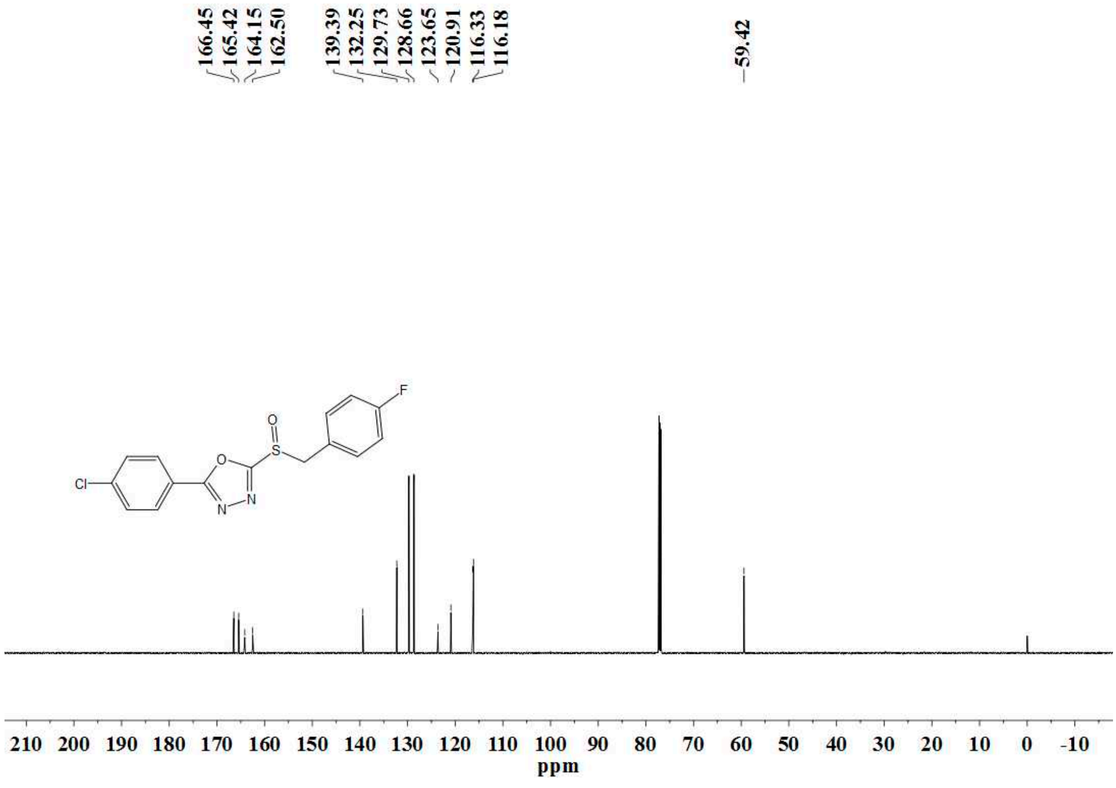

Figure S48-2. ^{13}C NMR spectrum of compound **7h**.

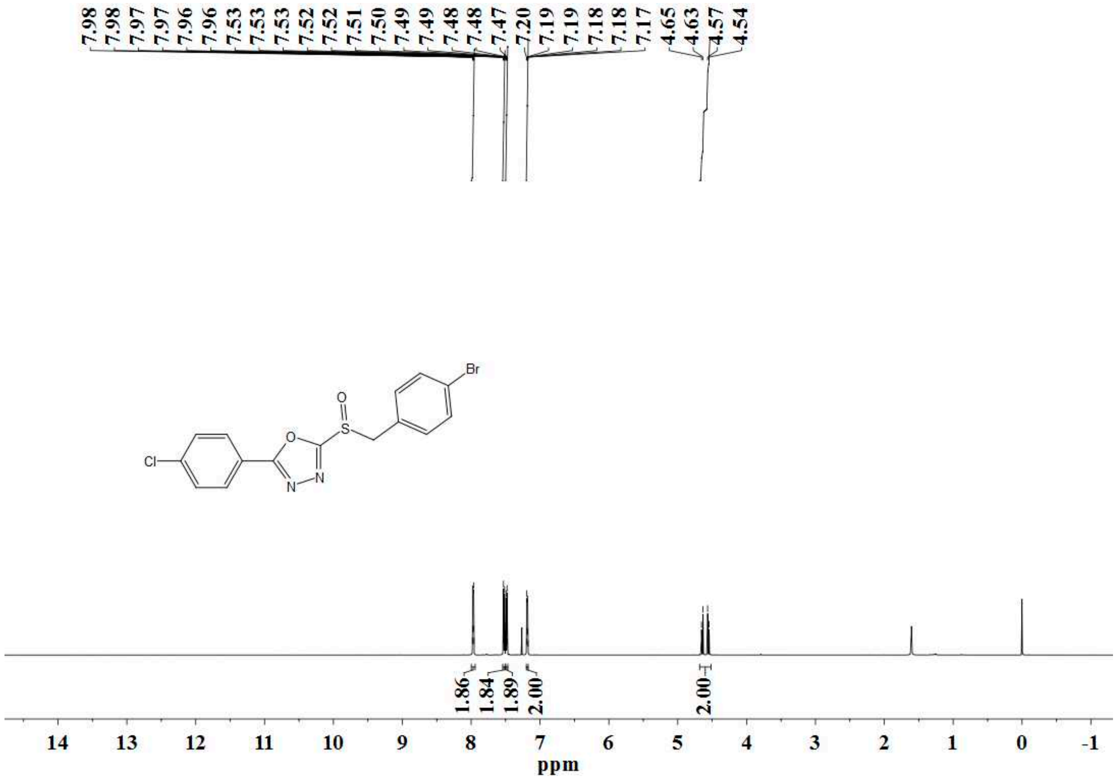

Figure S49-1. ^{1}H NMR spectrum of compound **7i**.

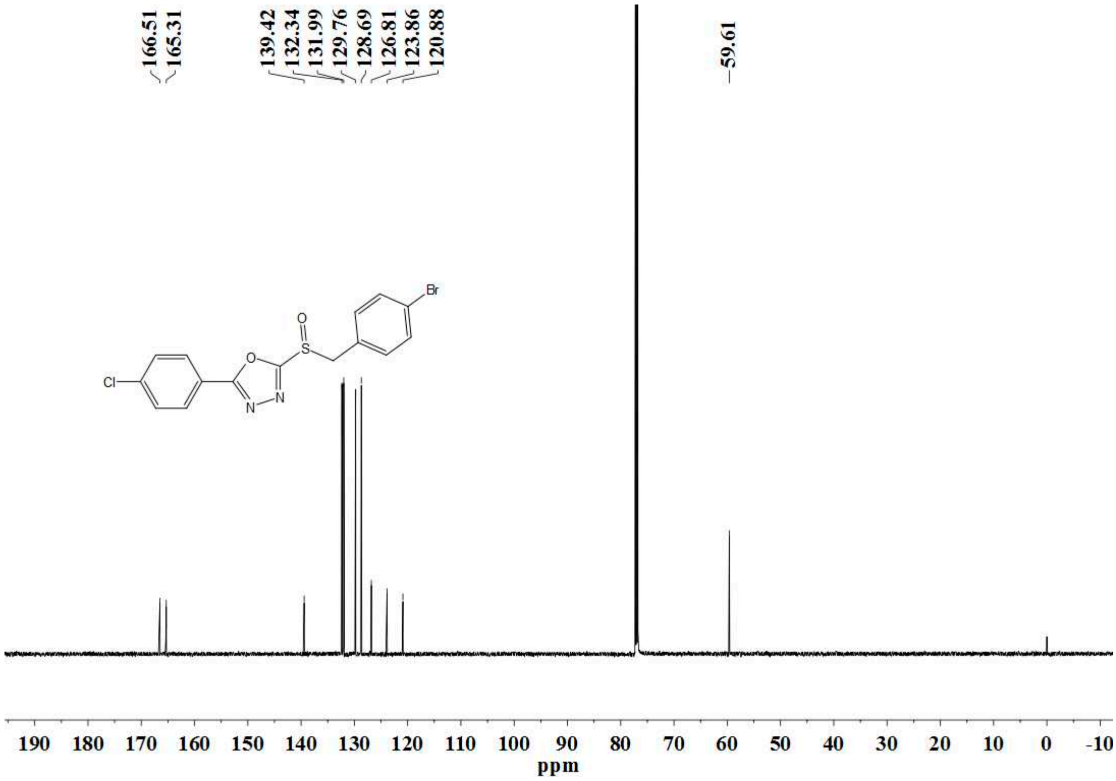

Figure S49-2. ^{13}C NMR spectrum of compound **7i**.

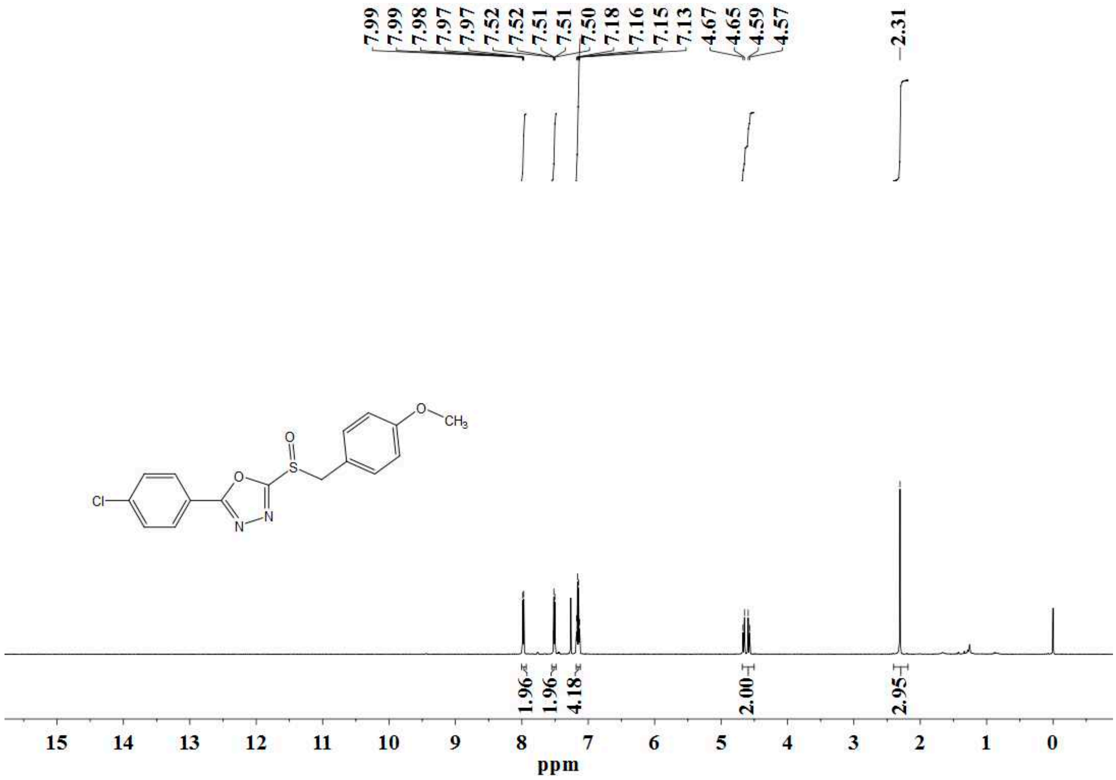

Figure S50-1. ^{1}H NMR spectrum of compound **7l**.

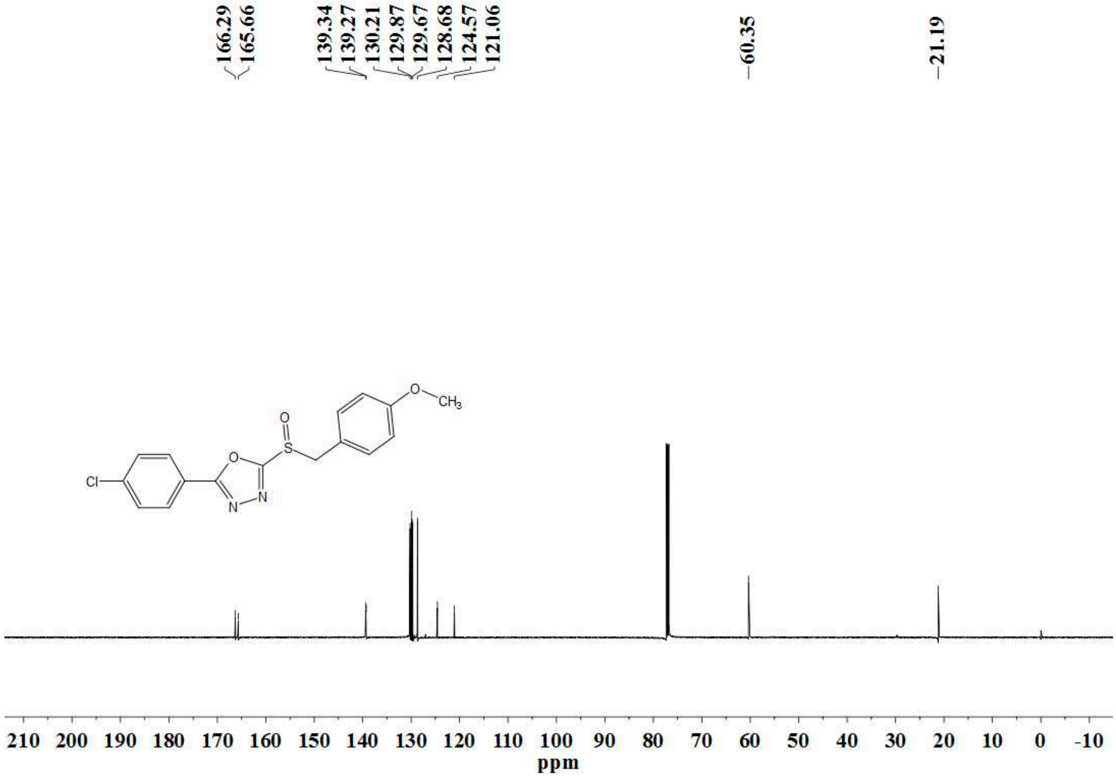

Figure S50-2. ^{13}C NMR spectrum of compound **7l**.

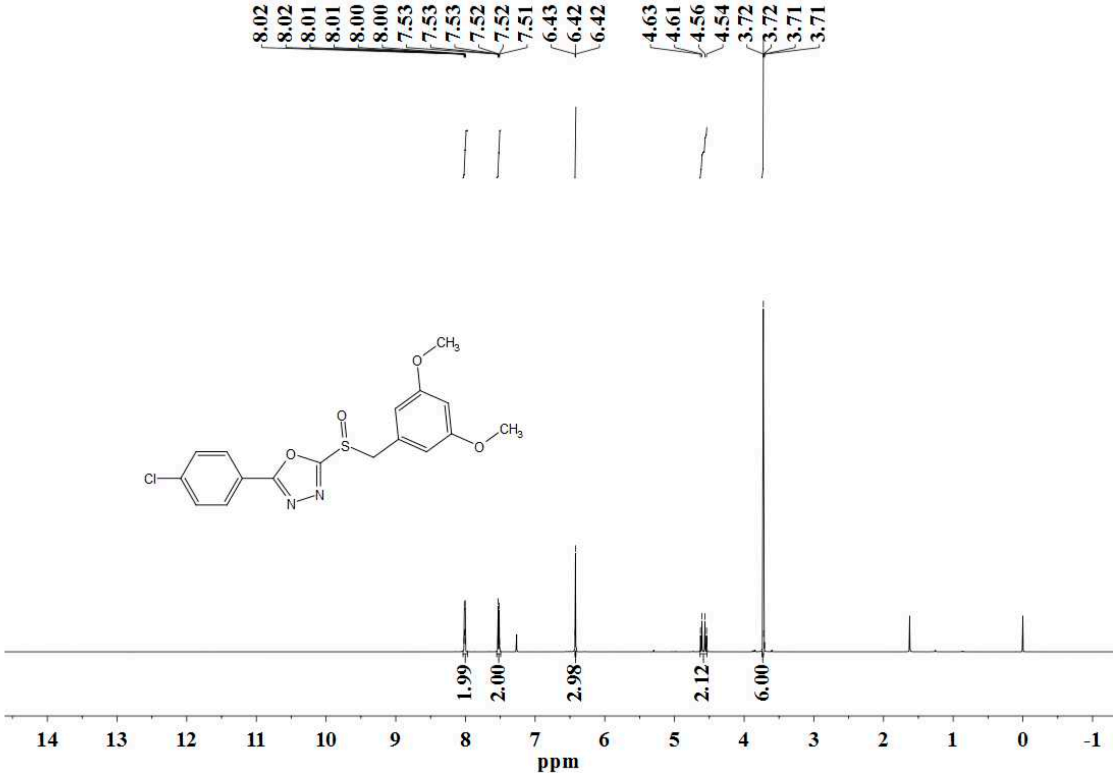

Figure S51-1. ^{1}H NMR spectrum of compound **7m**.

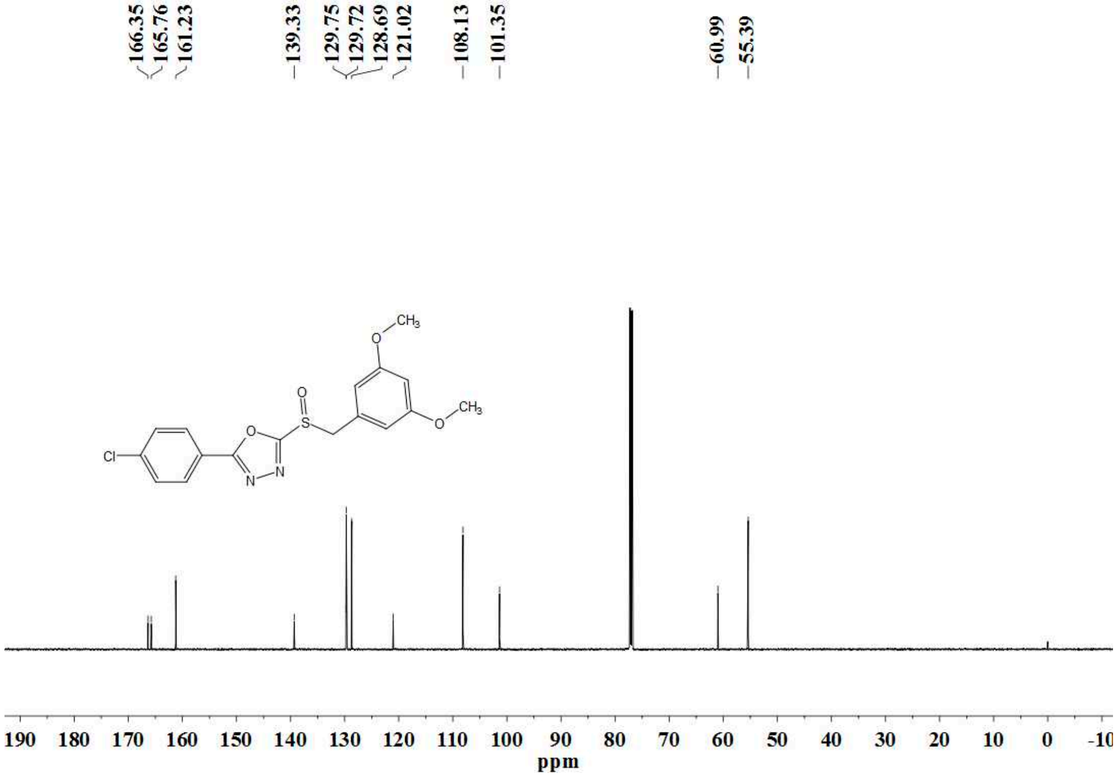

Figure S51-2. ^{13}C NMR spectrum of compound **7m**.

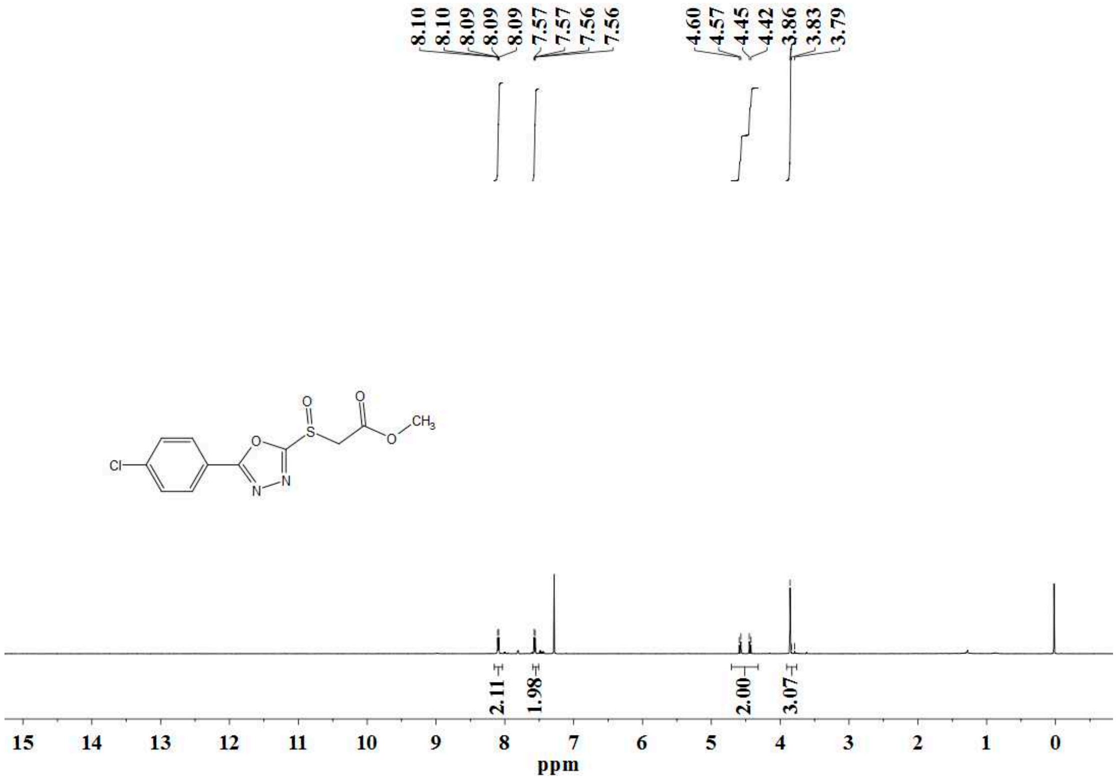

Figure S52-1. ^{1}H NMR spectrum of compound **7n**.

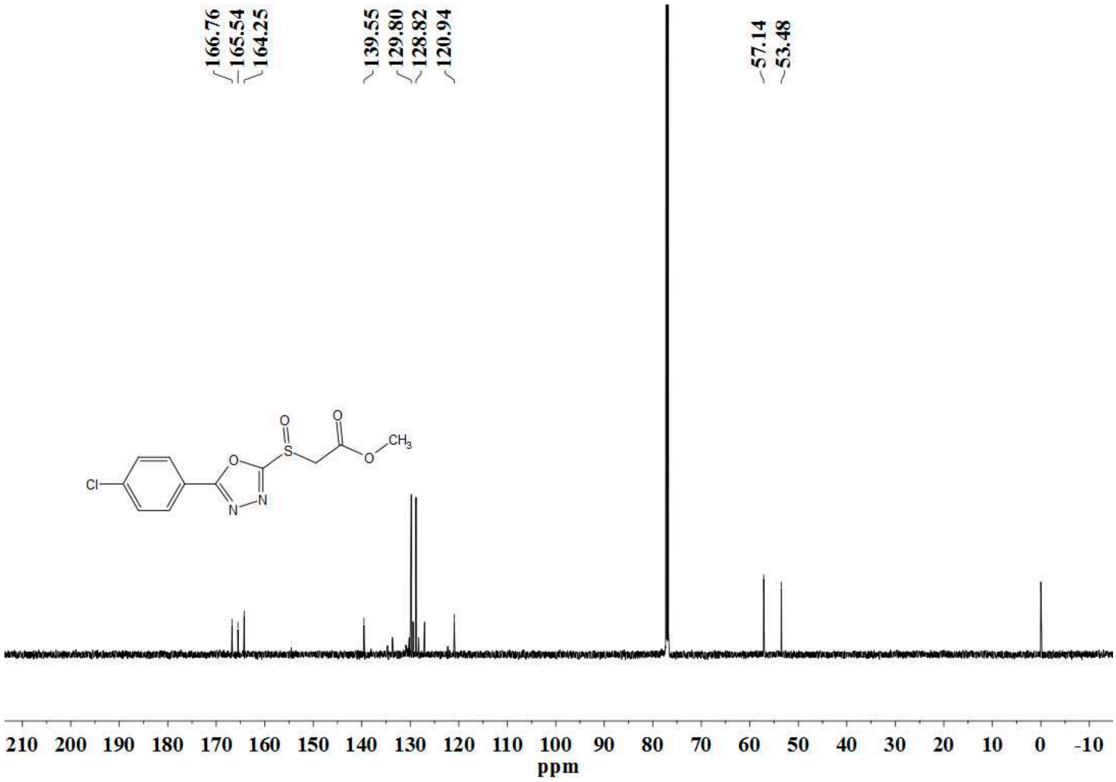

Figure S52-2. ^{13}C NMR spectrum of compound **7n**.

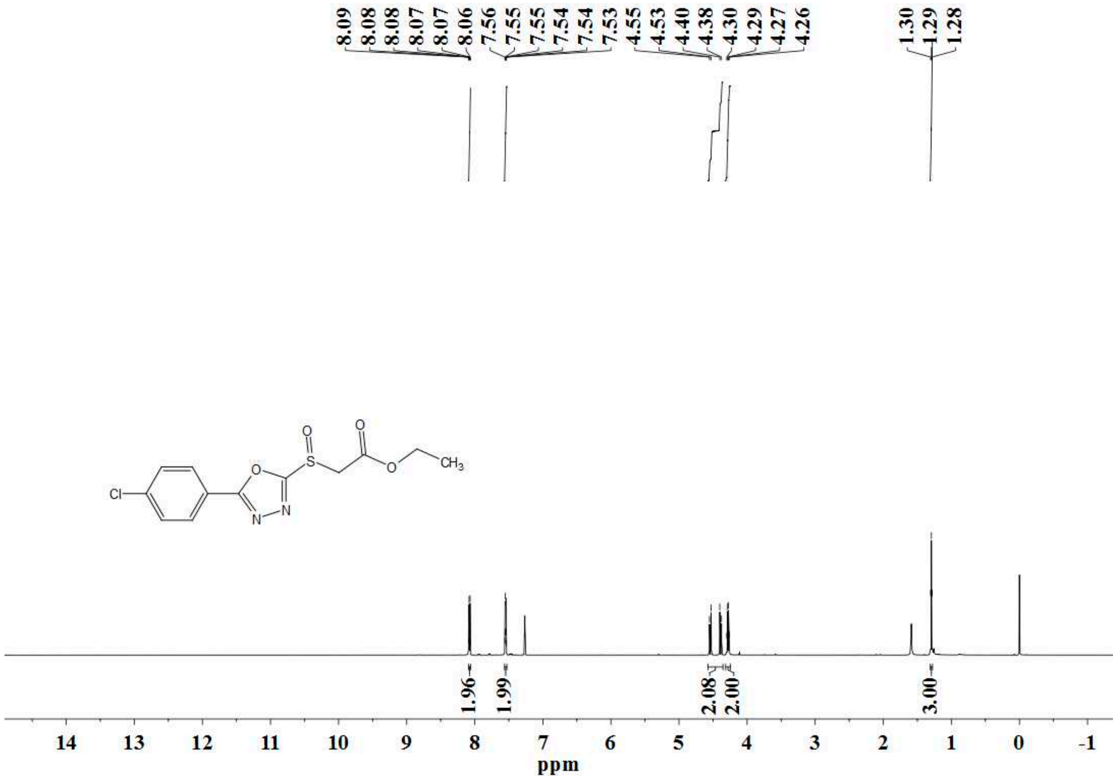

Figure S53-1. ^{1}H NMR spectrum of compound **7o**.

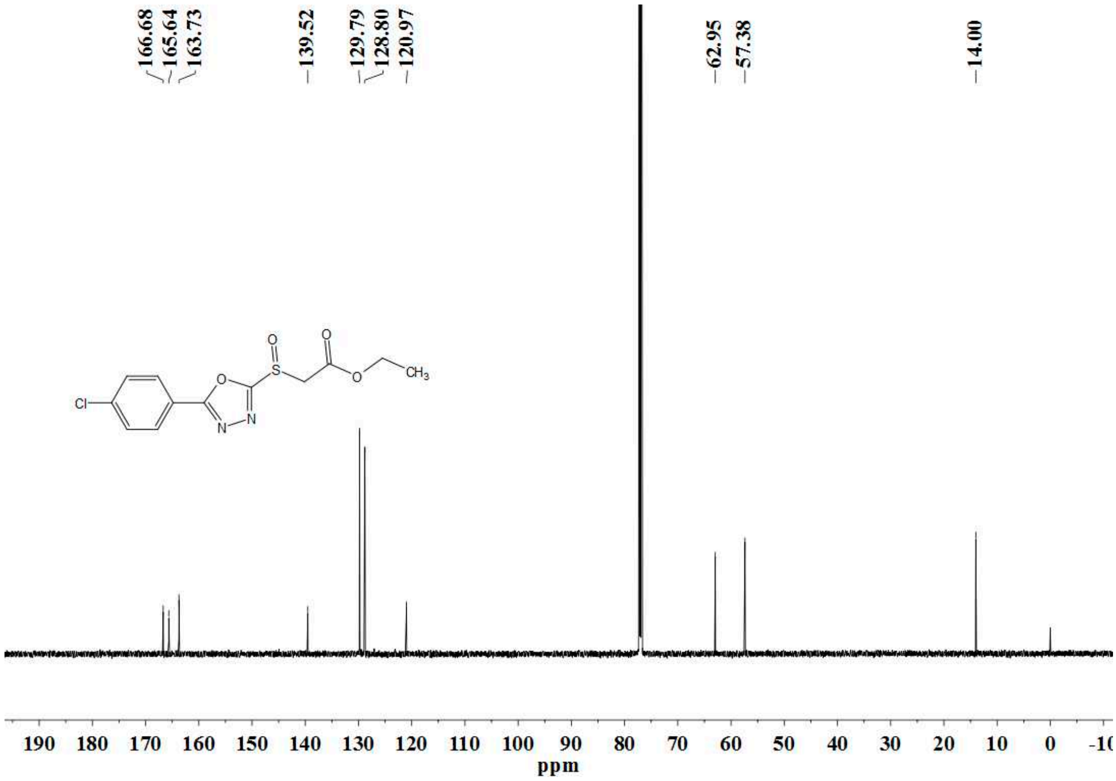

Figure S53-2. ^{13}C NMR spectrum of compound **7o**.

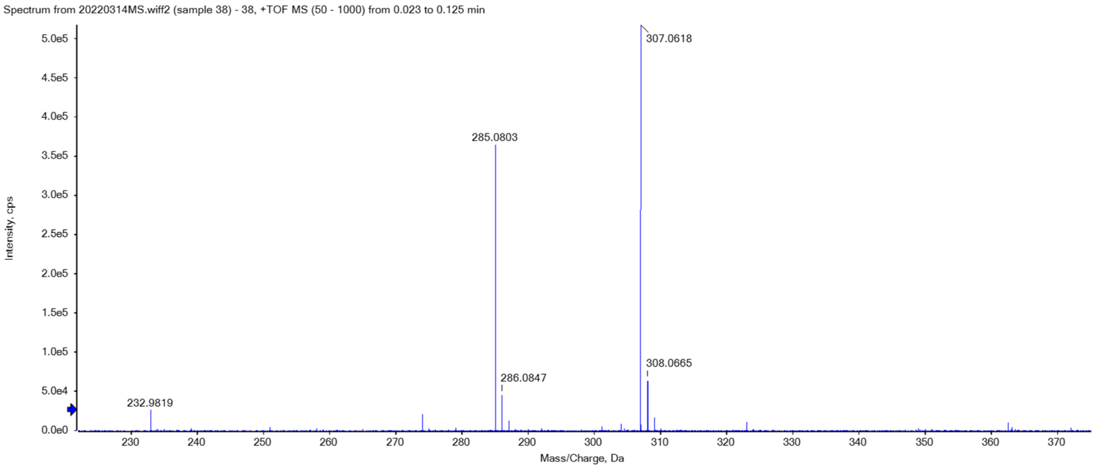

Figure S54. HRMS spectrum of compound **4a**.

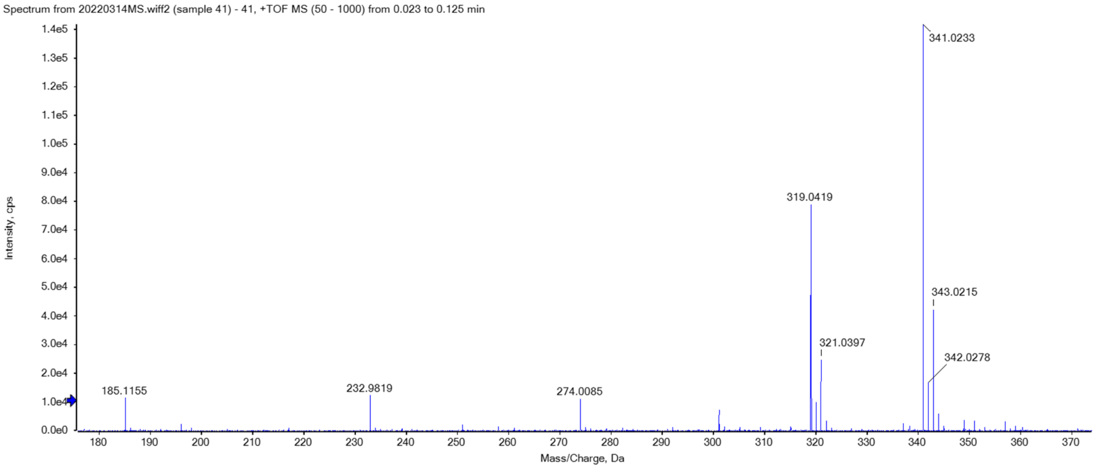

Figure S55. HRMS spectrum of compound **4b**.

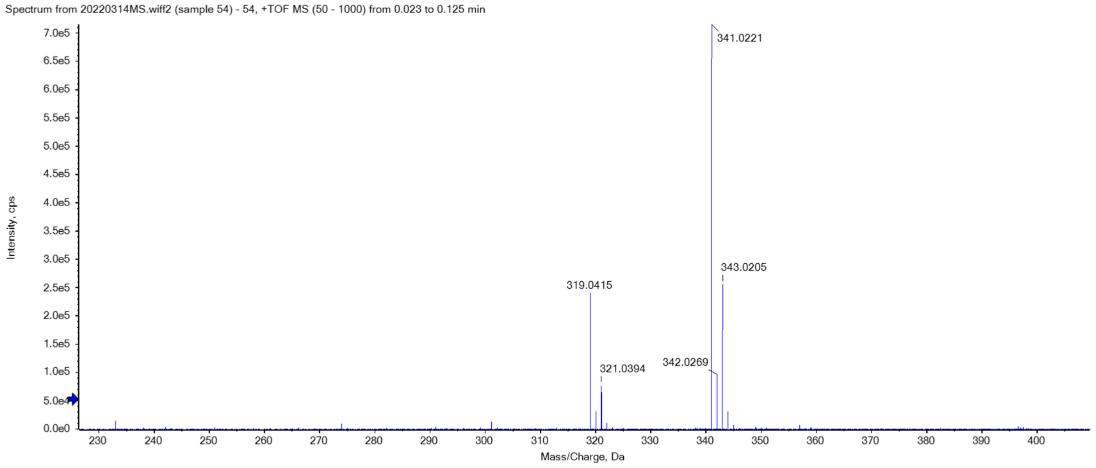

Figure S56. HRMS spectrum of compound **4c**.

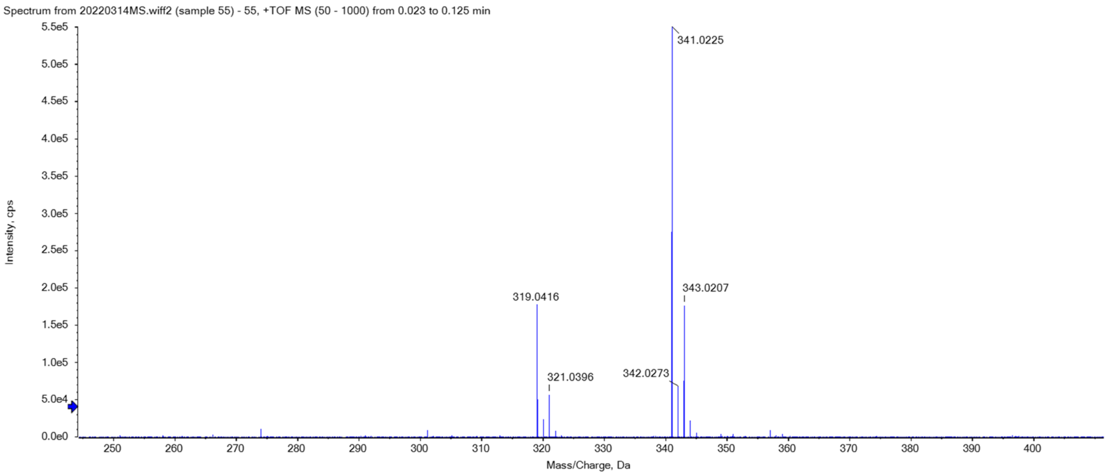

Figure S57. HRMS spectrum of compound **4d**.

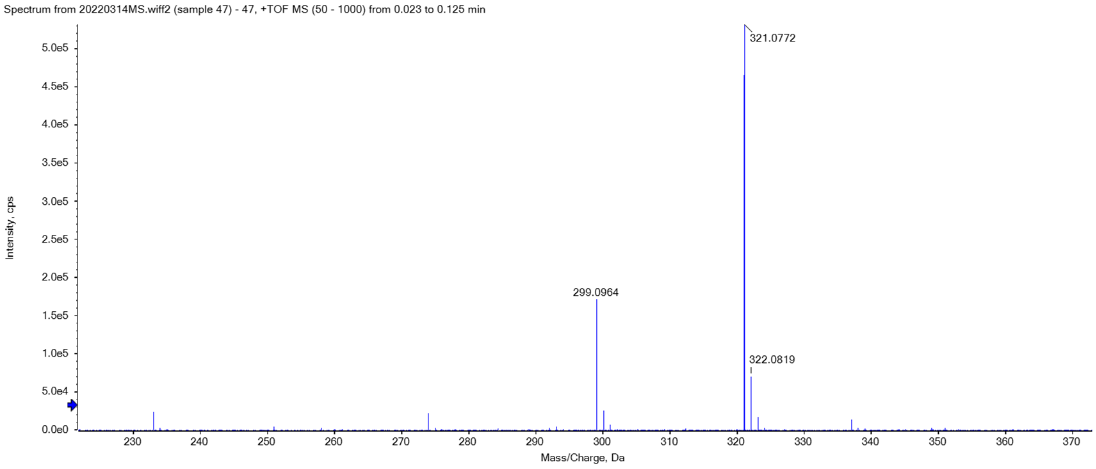

Figure S58. HRMS spectrum of compound **4e**.

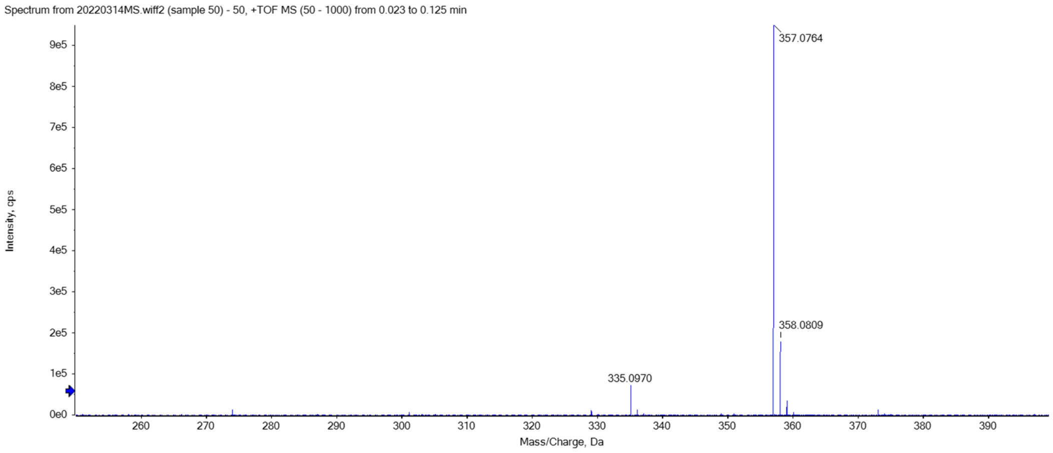

Figure S59. HRMS spectrum of compound **4f**.

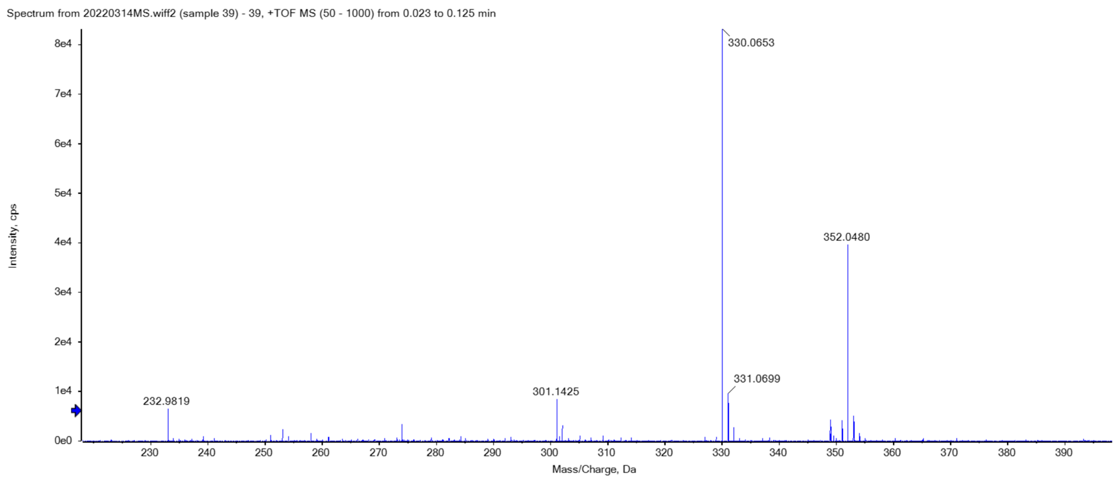

Figure S60. HRMS spectrum of compound **4g**.

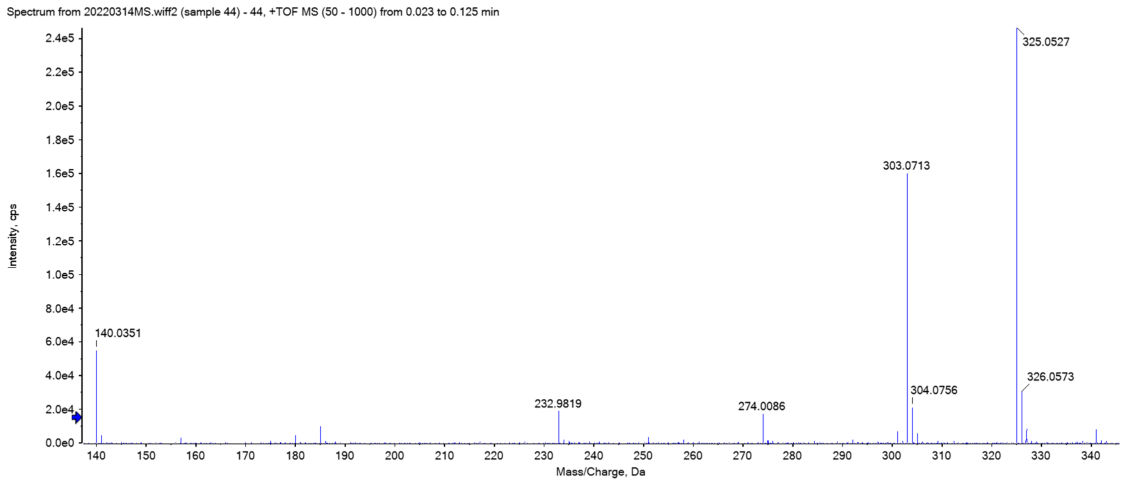

Figure S61. HRMS spectrum of compound **4h**.

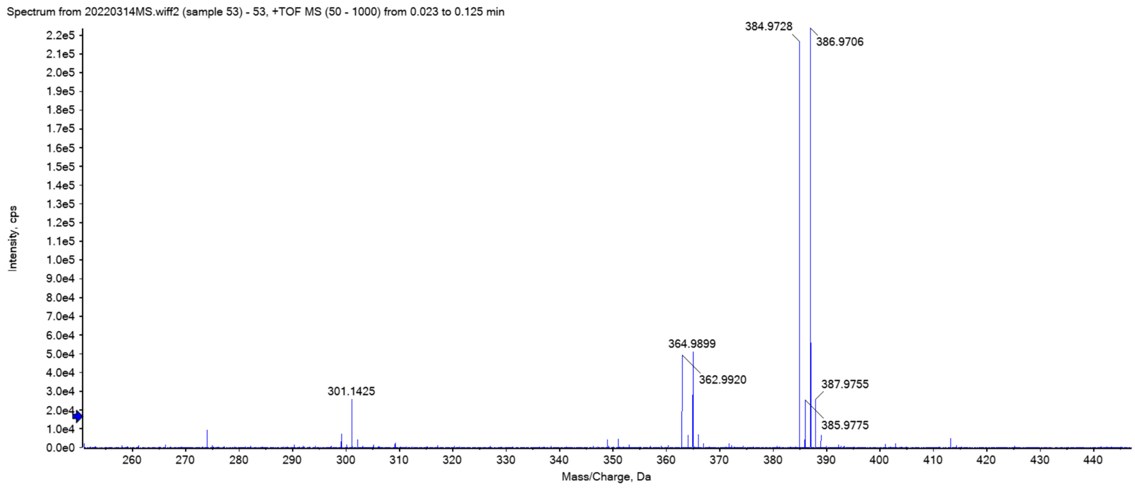

Figure S62. HRMS spectrum of compound **4i**.

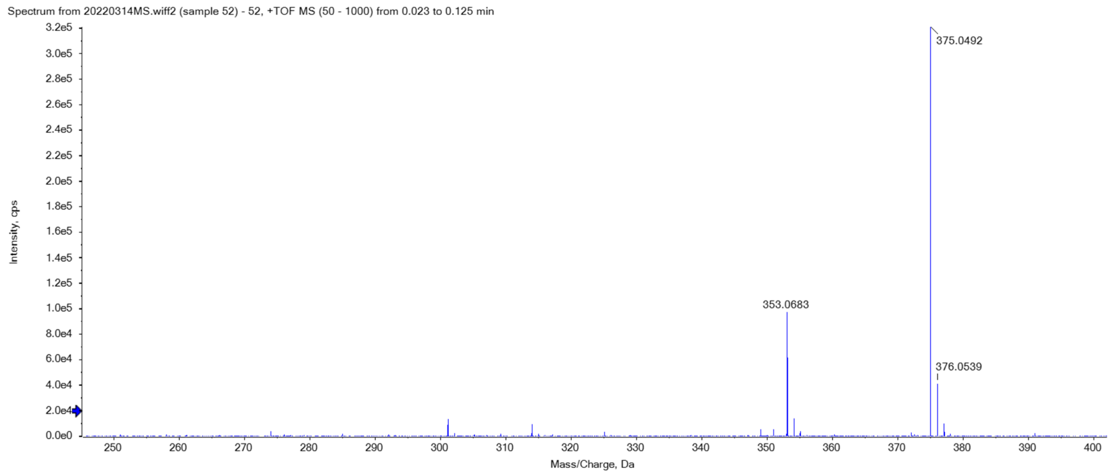

Figure S63. HRMS spectrum of compound **4j**.

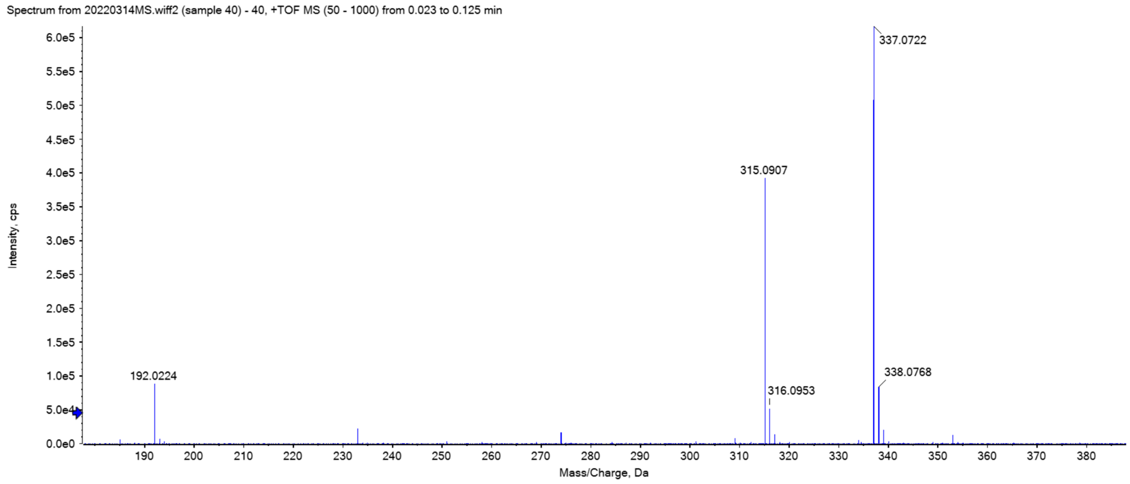

Figure S64. HRMS spectrum of compound **4k**.

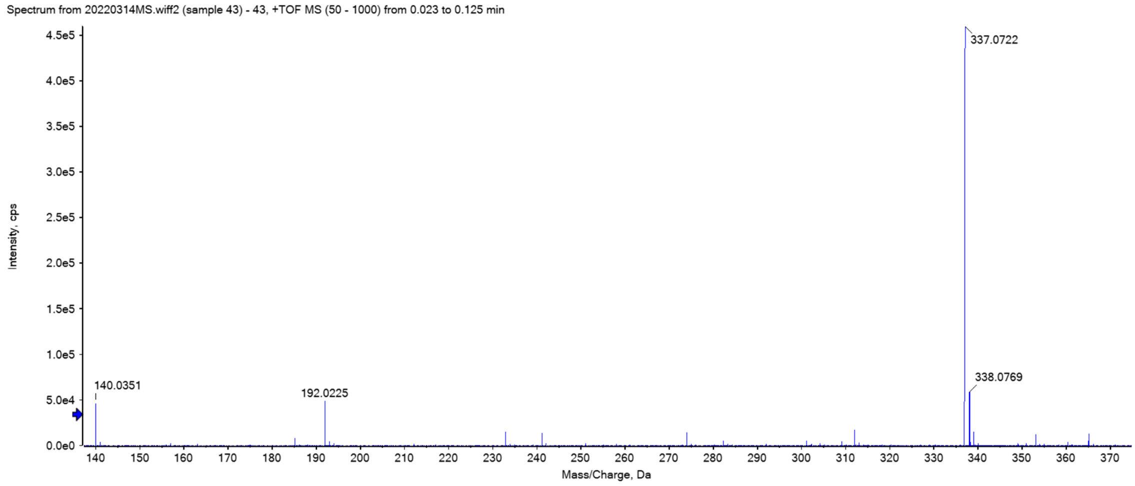

Figure S65. HRMS spectrum of compound **4l**.

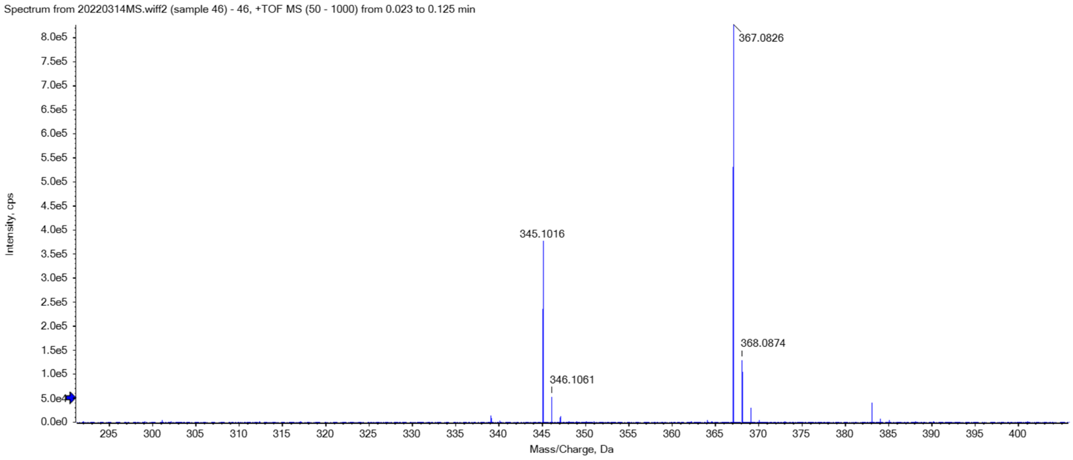

Figure S66. HRMS spectrum of compound **4m**.

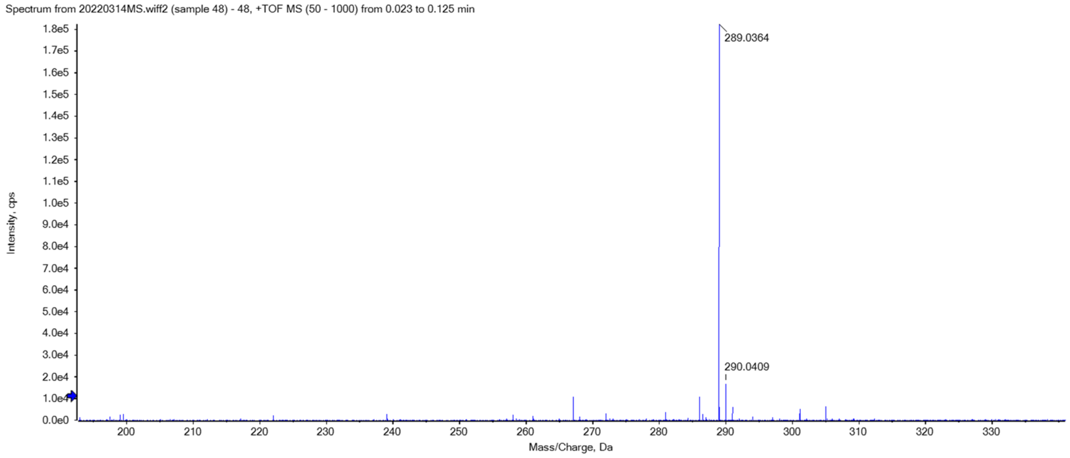

Figure S67. HRMS spectrum of compound **4n**.

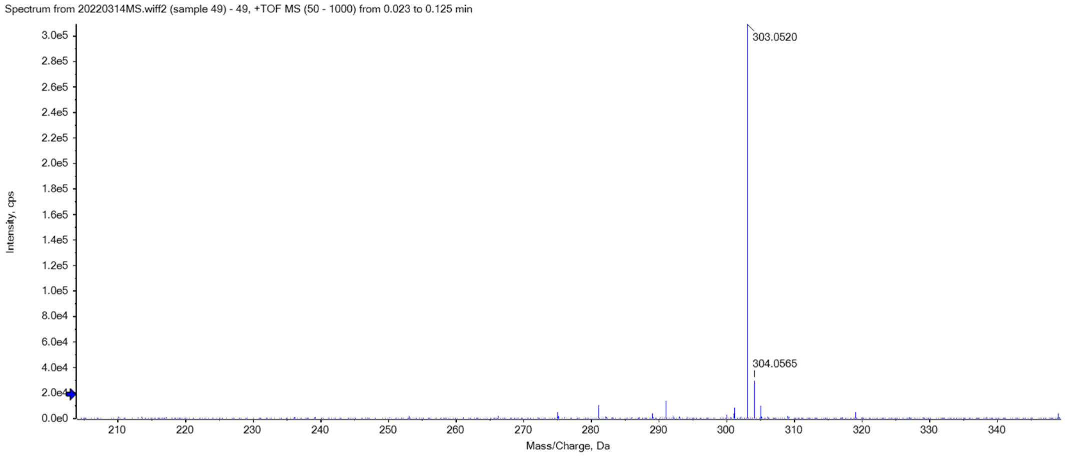

Figure S68. HRMS spectrum of compound **4o**.

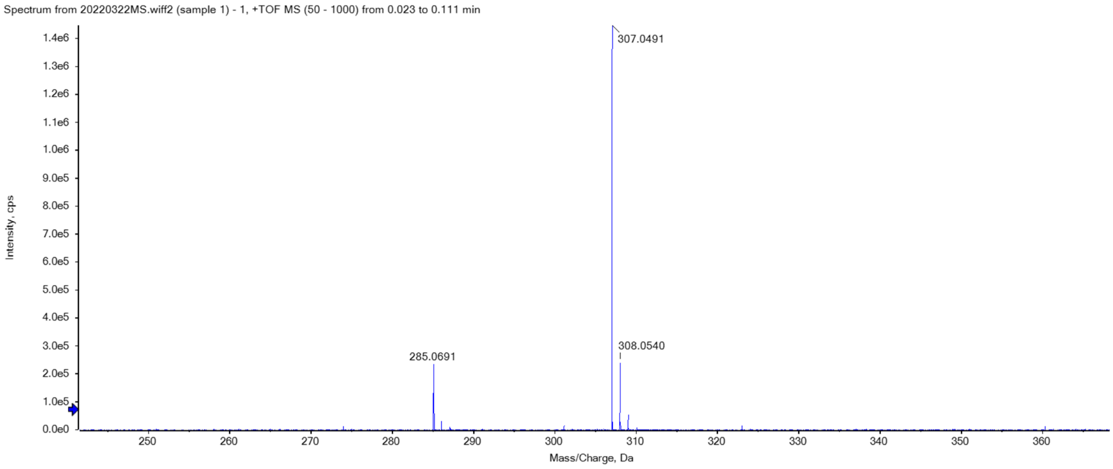

Figure S69. HRMS spectrum of compound **5a**.

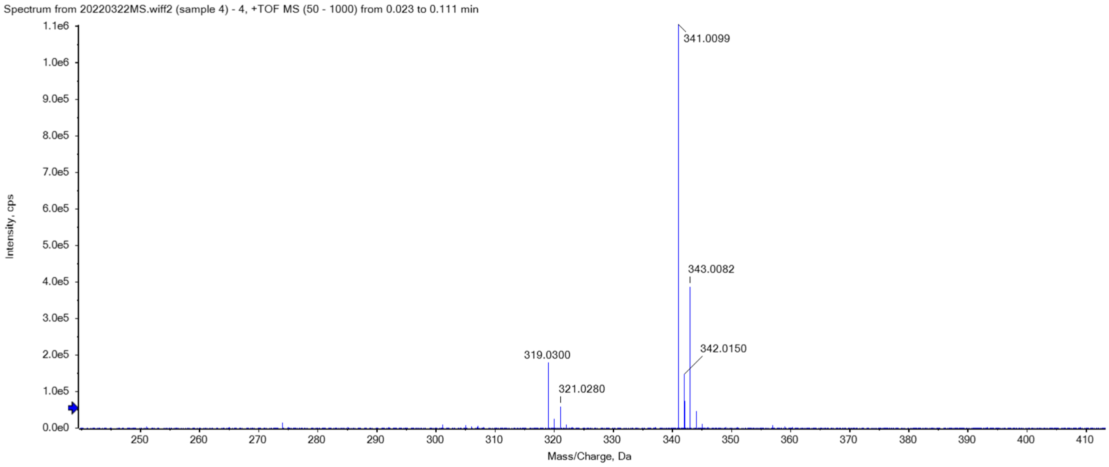

Figure S70. HRMS spectrum of compound **5b**.

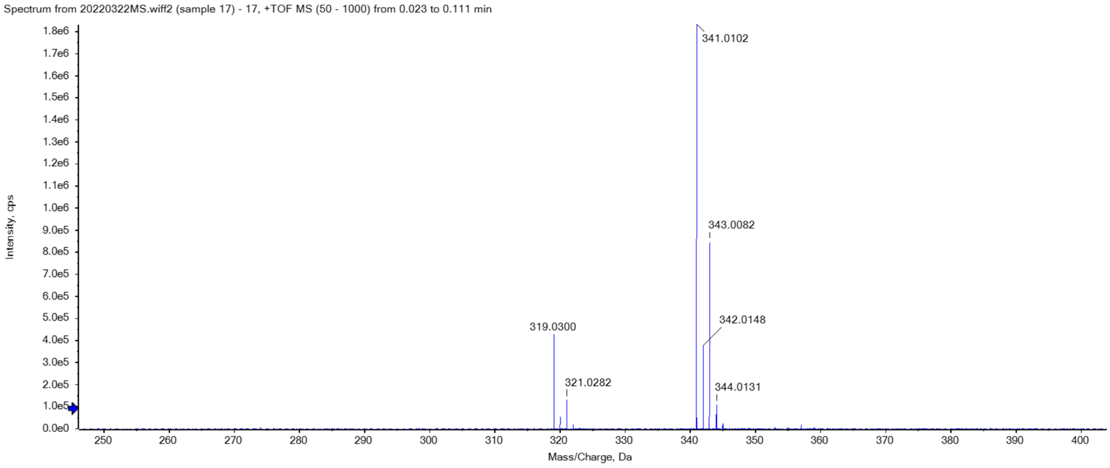

Figure S71. HRMS spectrum of compound **5c**.

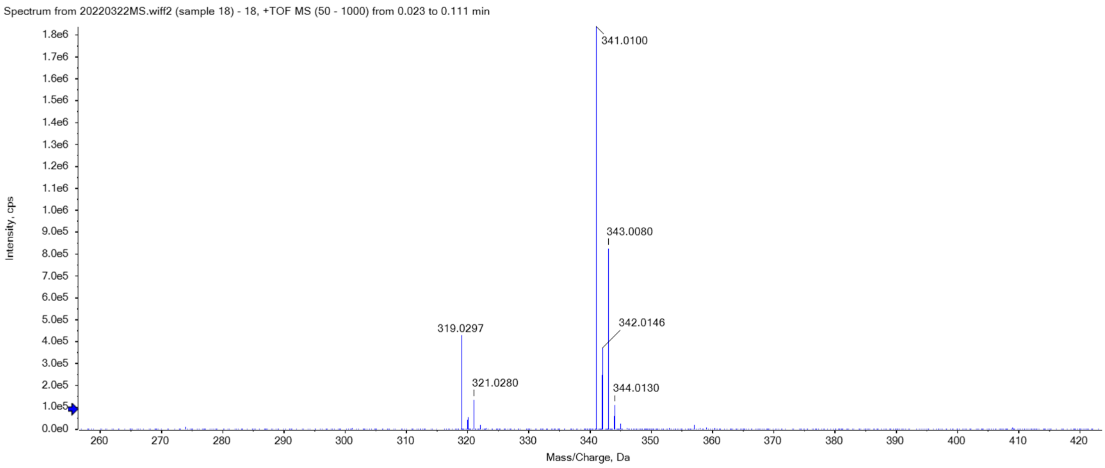

Figure S72. HRMS spectrum of compound **5d**.

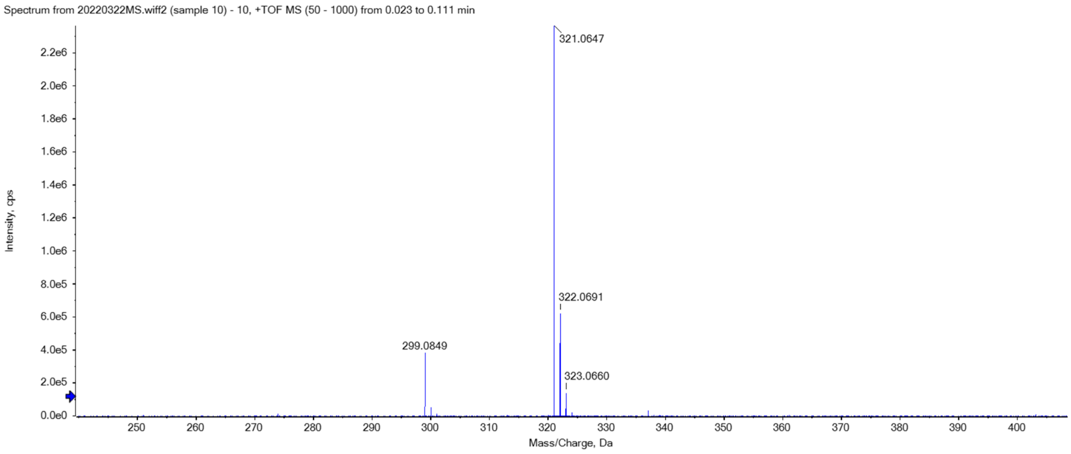

Figure S73. HRMS spectrum of compound **5e**.

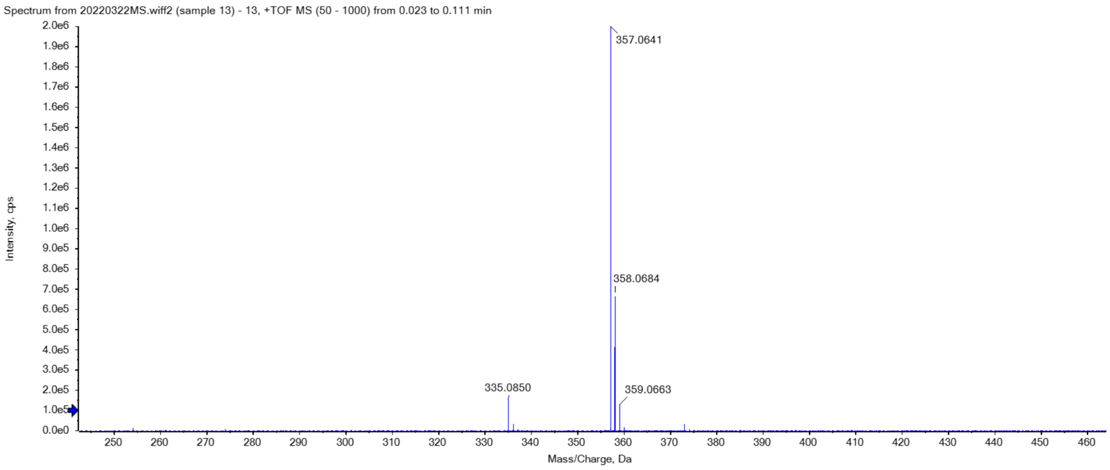

Figure S74. HRMS spectrum of compound **5f**.

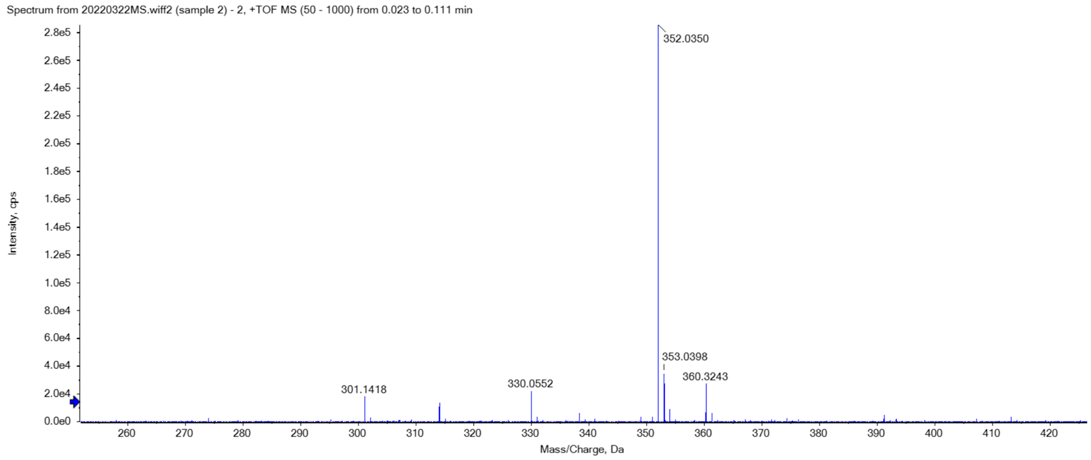

Figure S75. HRMS spectrum of compound **5g**.

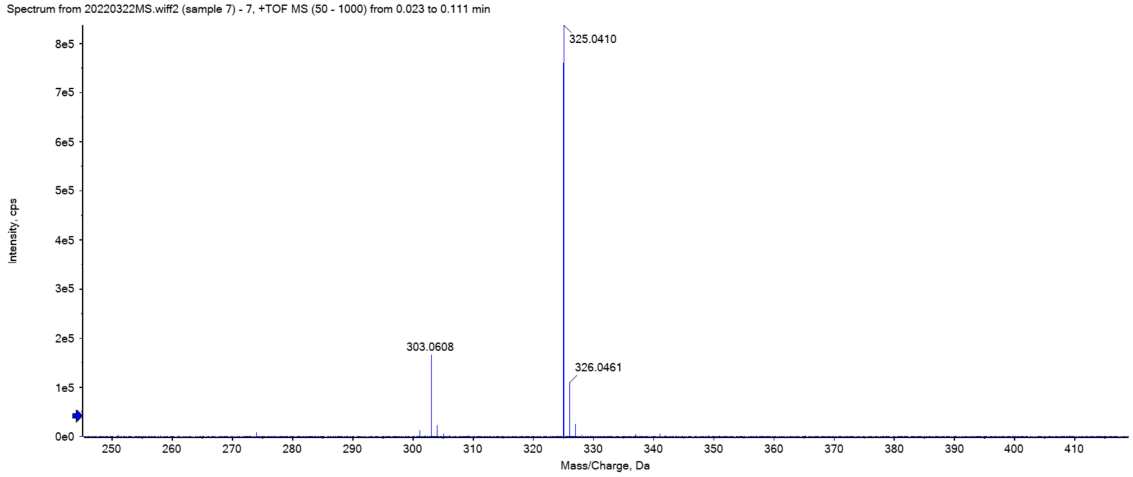

Figure S76. HRMS spectrum of compound **5h**.

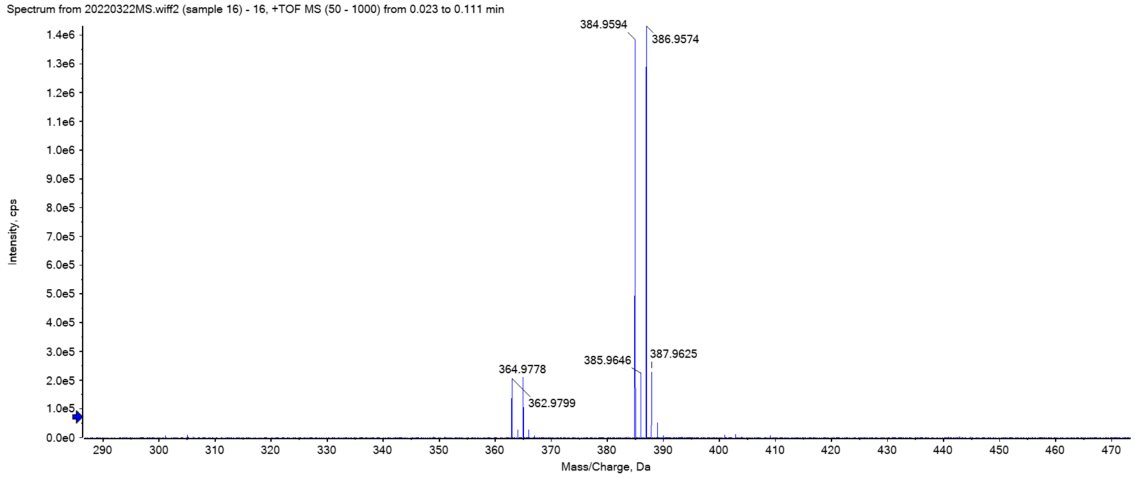

Figure S77. HRMS spectrum of compound **5i**.

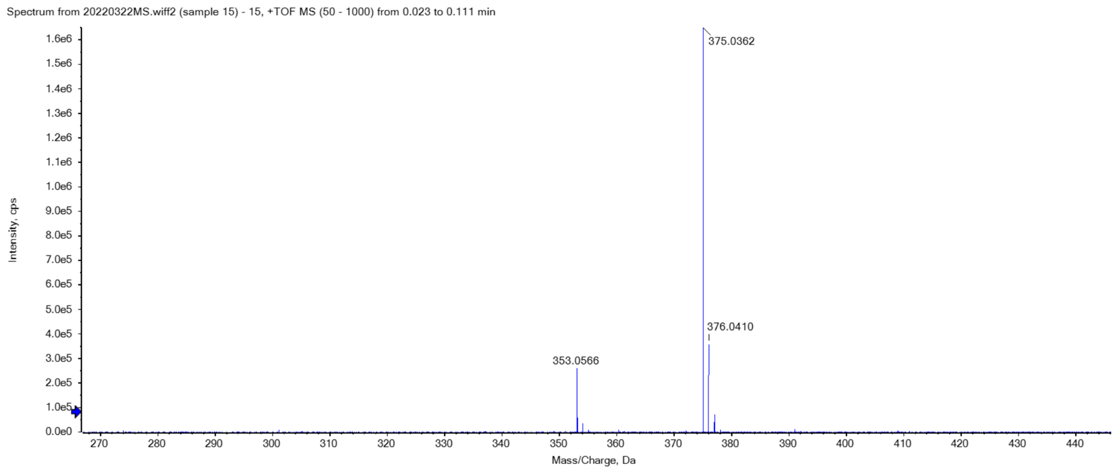

Figure S78. HRMS spectrum of compound **5j**.

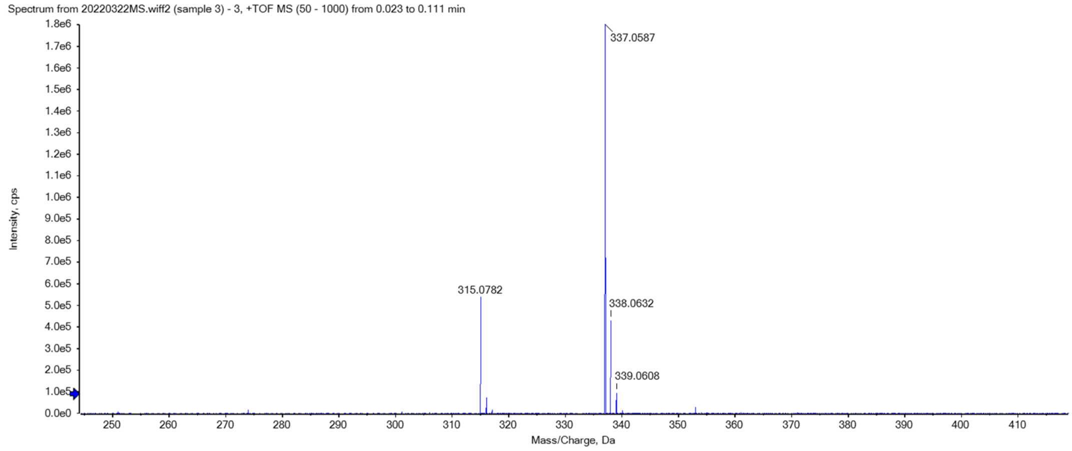

Figure S79. HRMS spectrum of compound **5k**.

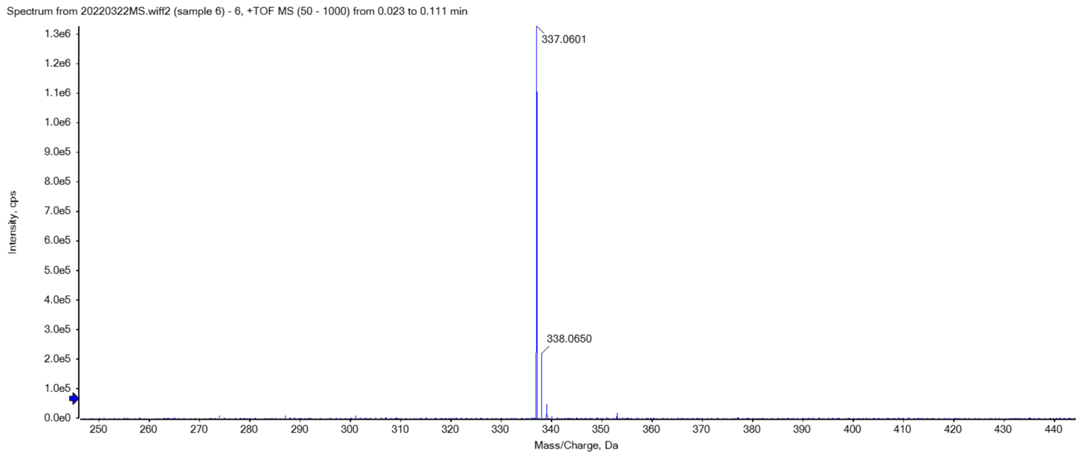

Figure S80. HRMS spectrum of compound **5l**.

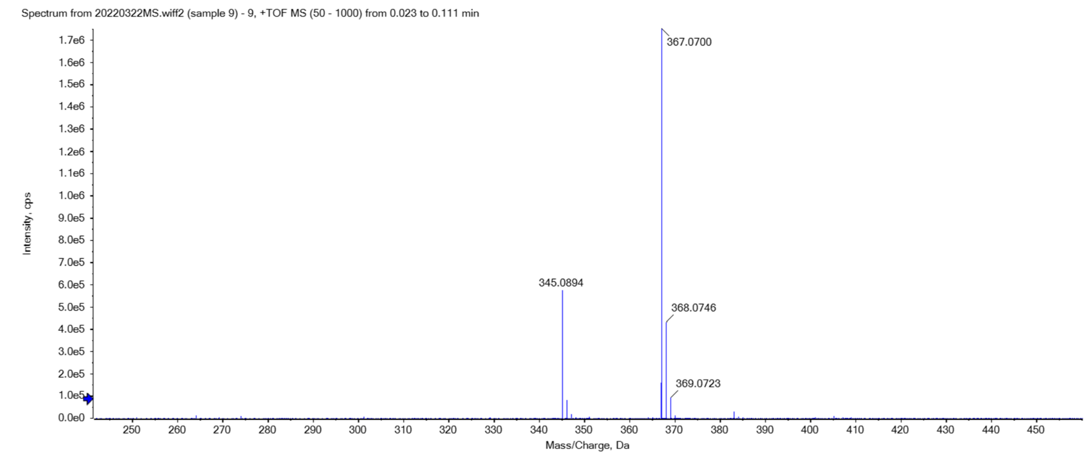

Figure S81. HRMS spectrum of compound **5m**.

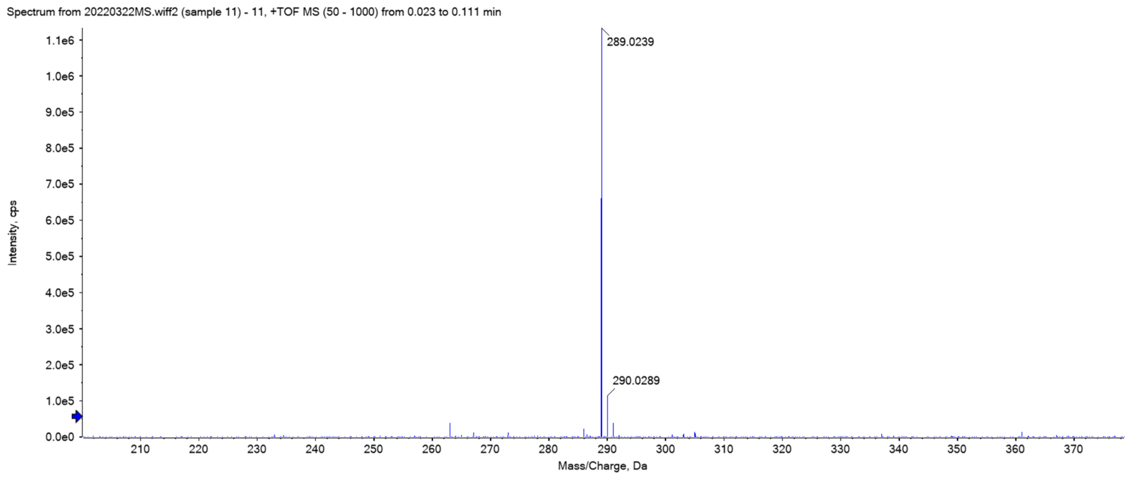

Figure S82. HRMS spectrum of compound **5n**.

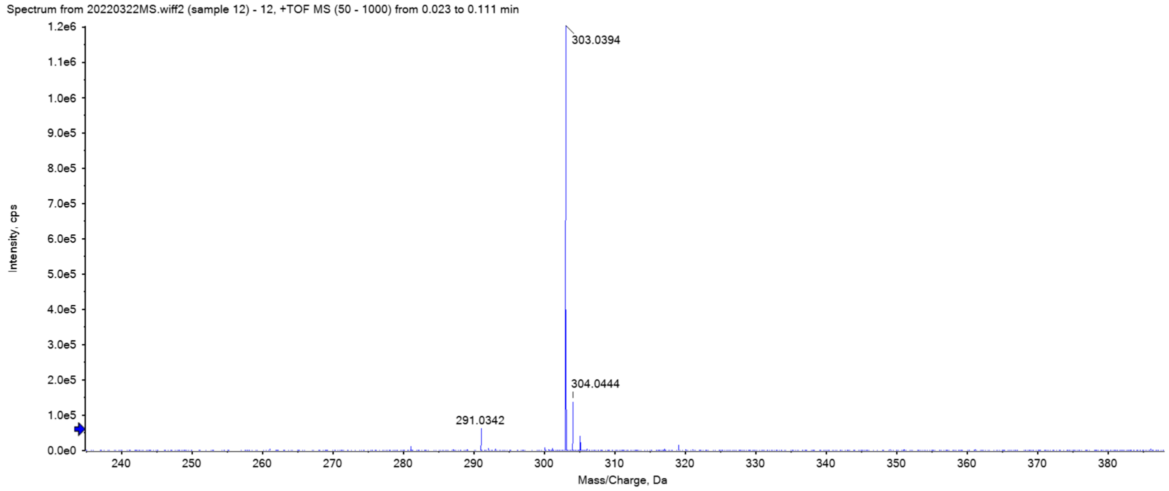

Figure S83. HRMS spectrum of compound **5o**.

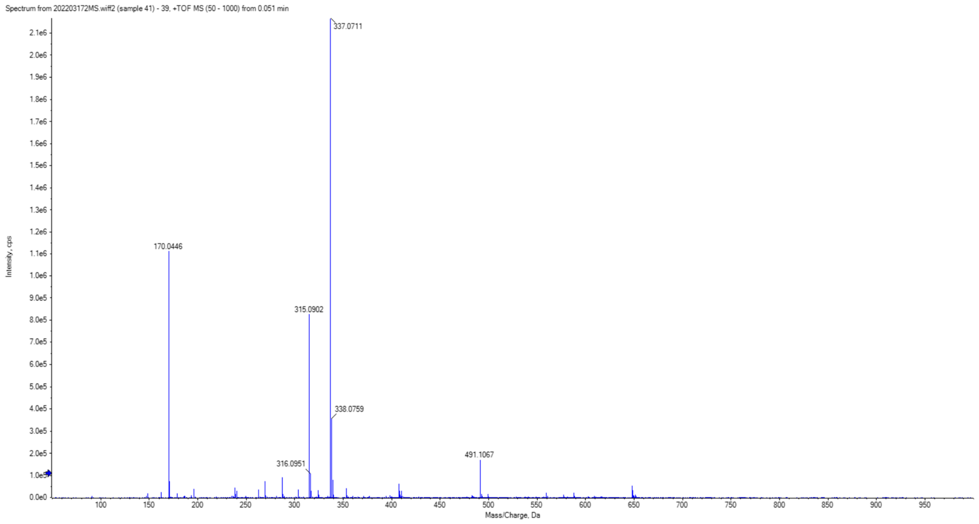

Figure S84. HRMS spectrum of compound **6a**.

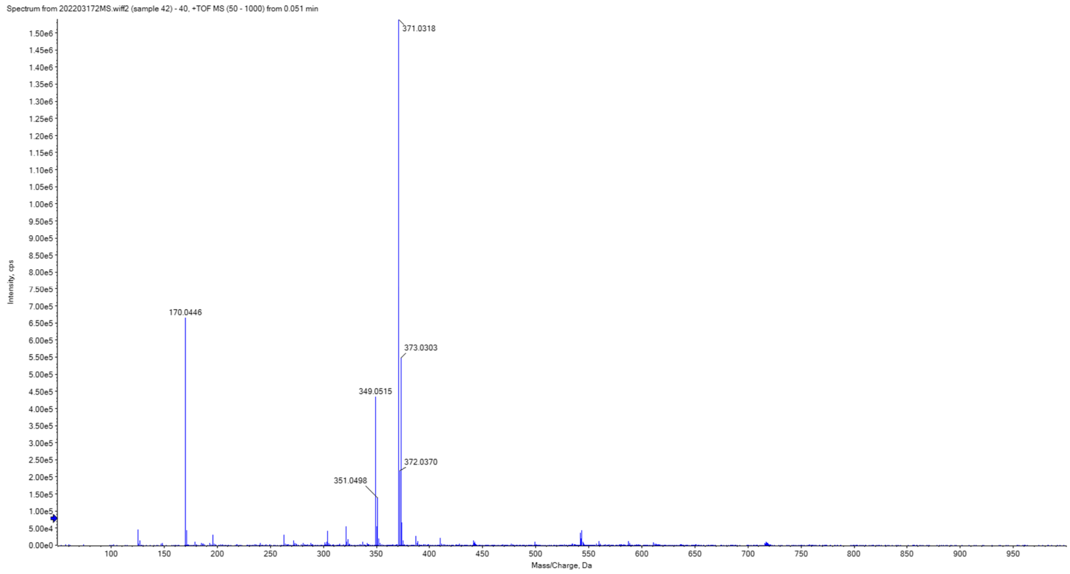

Figure S85. HRMS spectrum of compound **6b**.

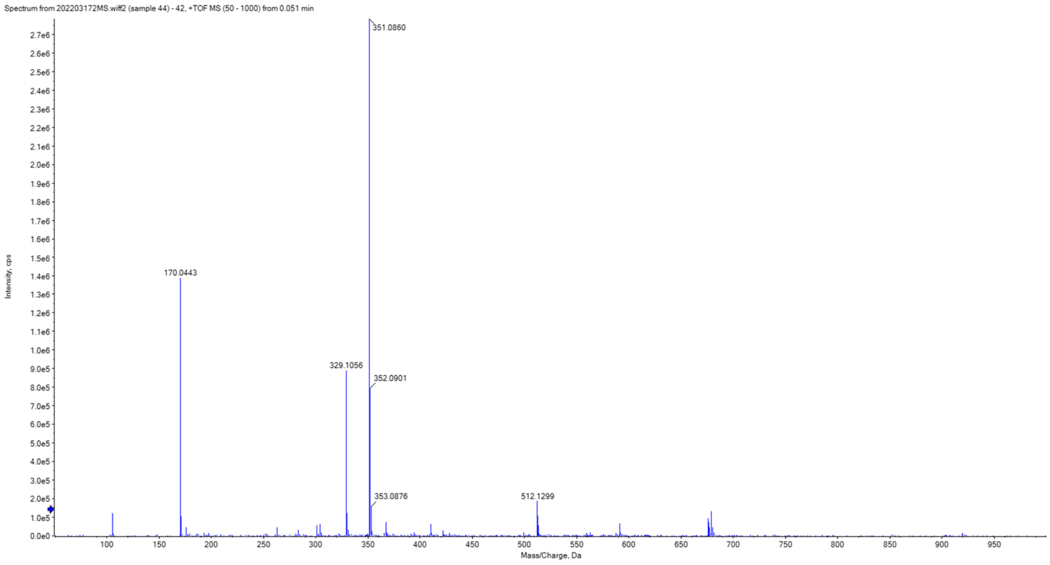

Figure S86. HRMS spectrum of compound **6e**.

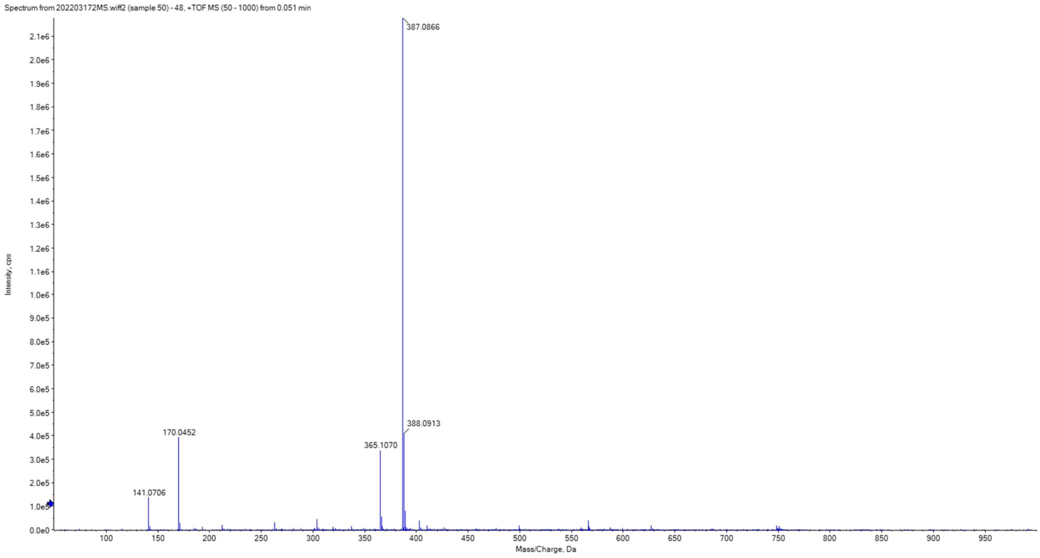

Figure S87. HRMS spectrum of compound **6f**.

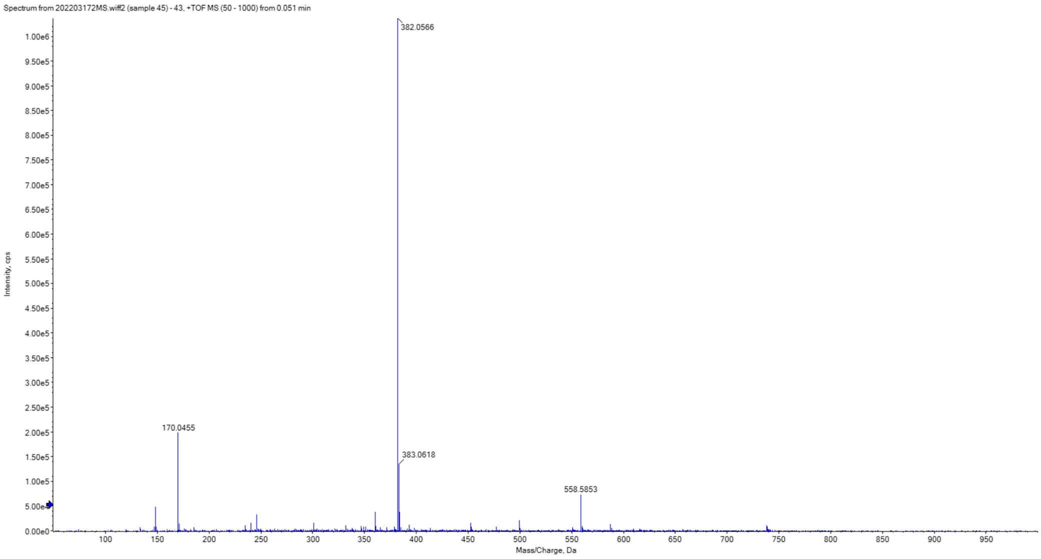

Figure S88. HRMS spectrum of compound **6g**.

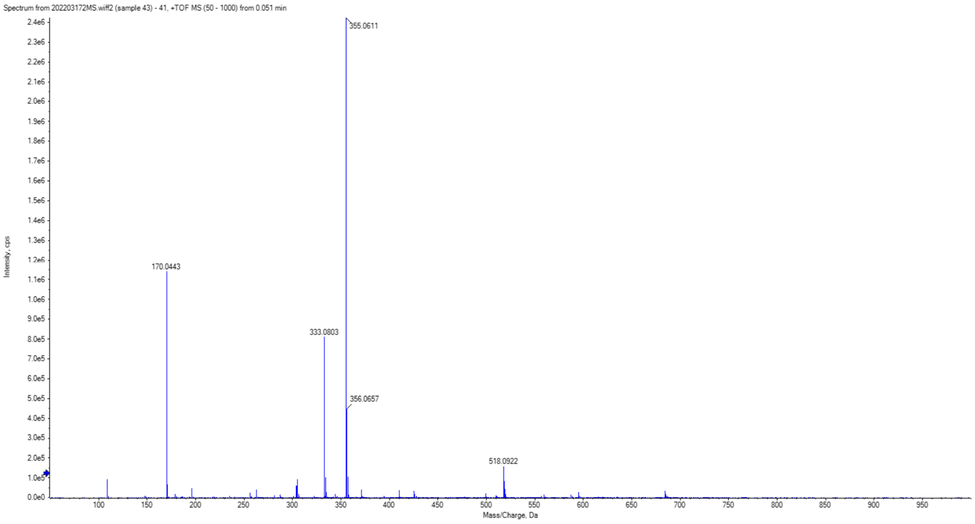

Figure S89. HRMS spectrum of compound **6h**

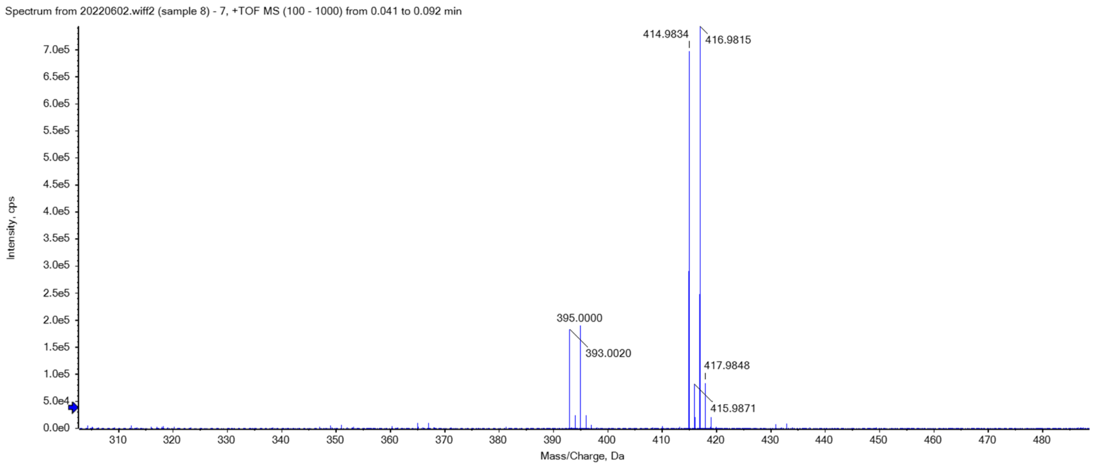

Figure S90. HRMS spectrum of compound **6i**

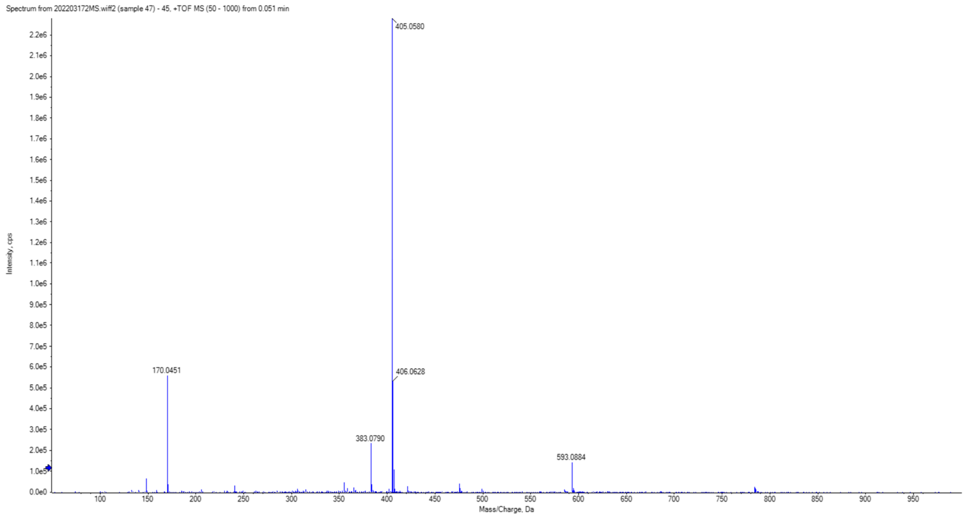

Figure S91. HRMS spectrum of compound **6j**.

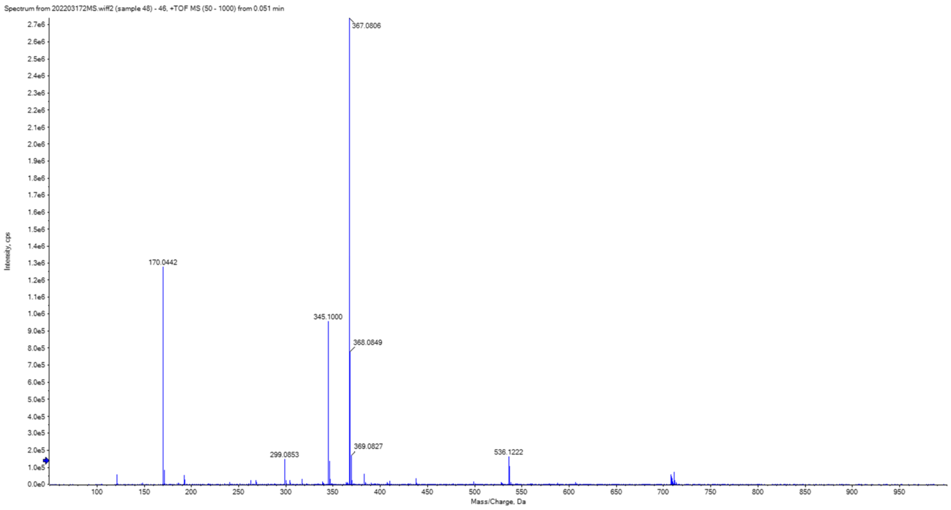

Figure S92. HRMS spectrum of compound **6k**.

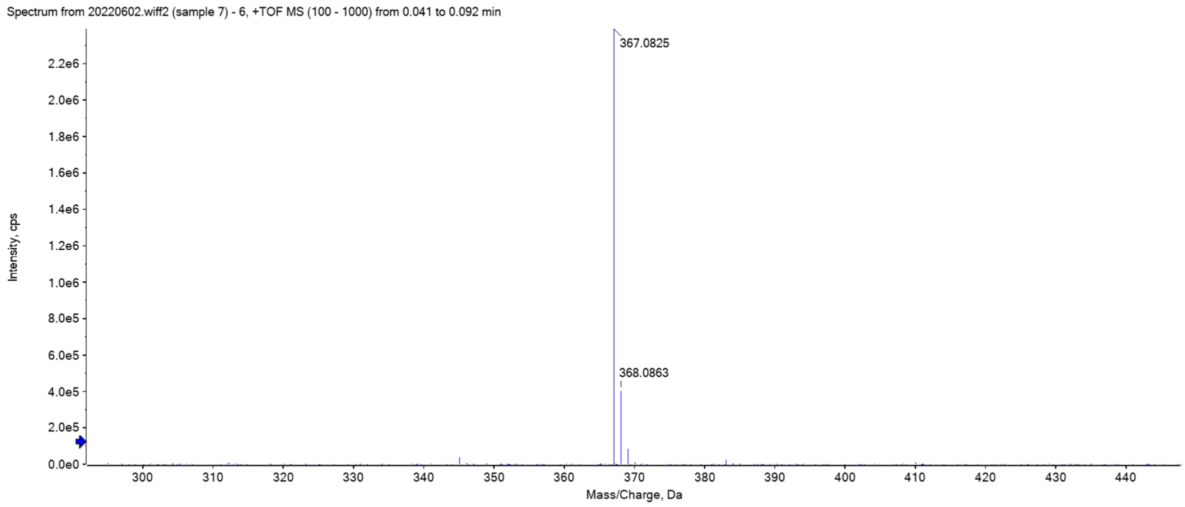

Figure S93. HRMS spectrum of compound **6l**.

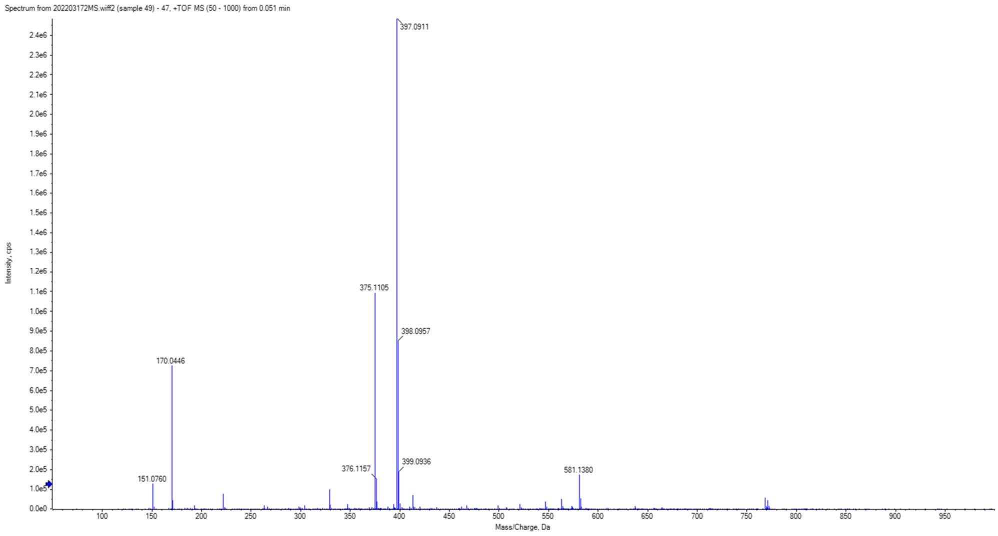

Figure S94. HRMS spectrum of compound **6m**.

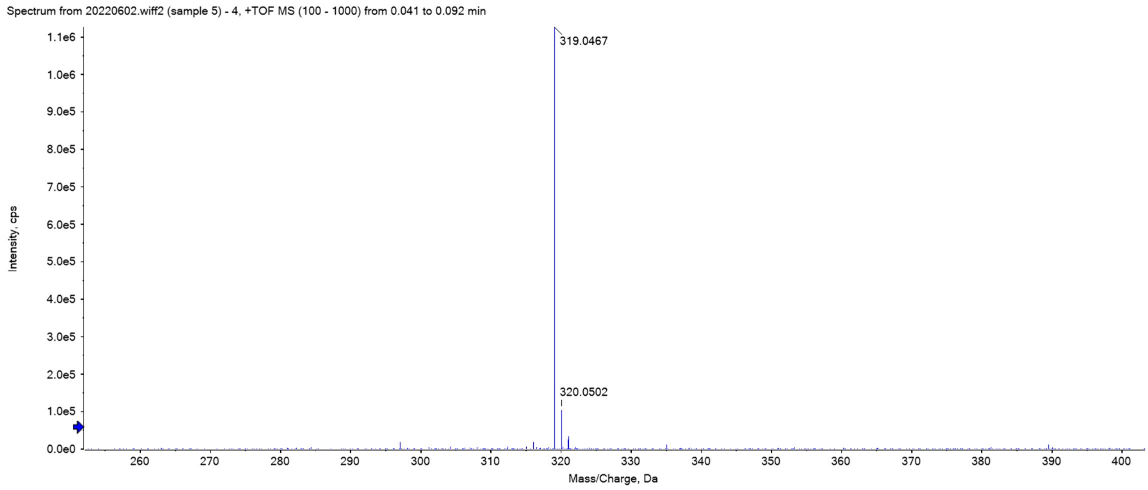

Figure S95. HRMS spectrum of compound **6n**.

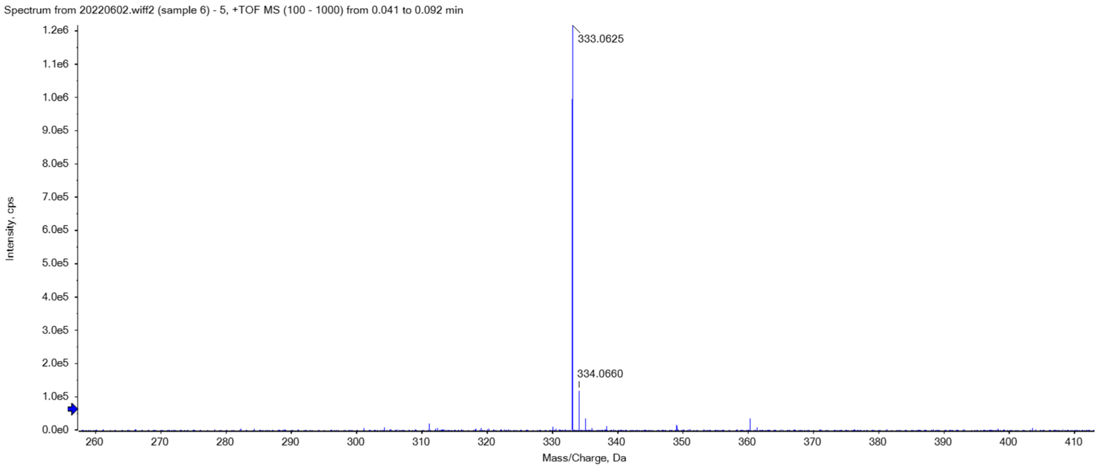

Figure S96. HRMS spectrum of compound **6o**.

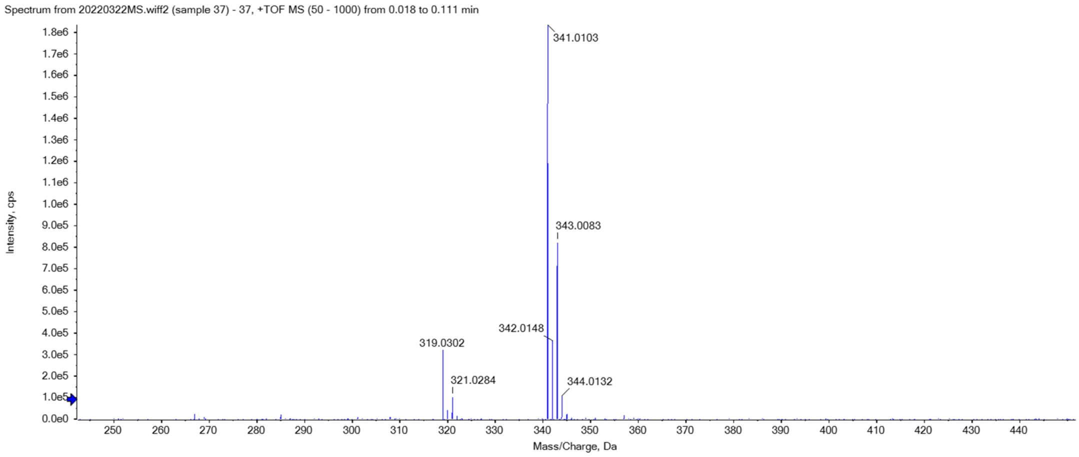

Figure S97. HRMS spectrum of compound **7a**.

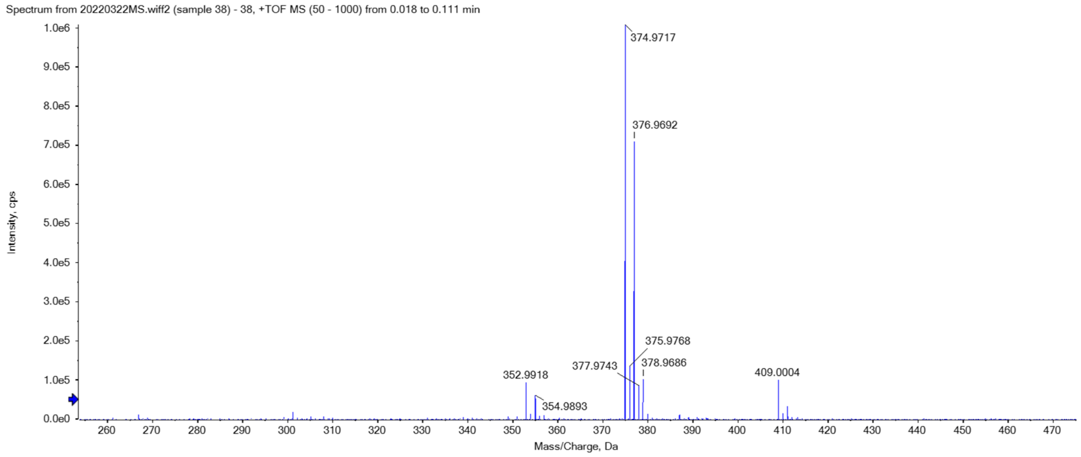

Figure S98. HRMS spectrum of compound **7b**.

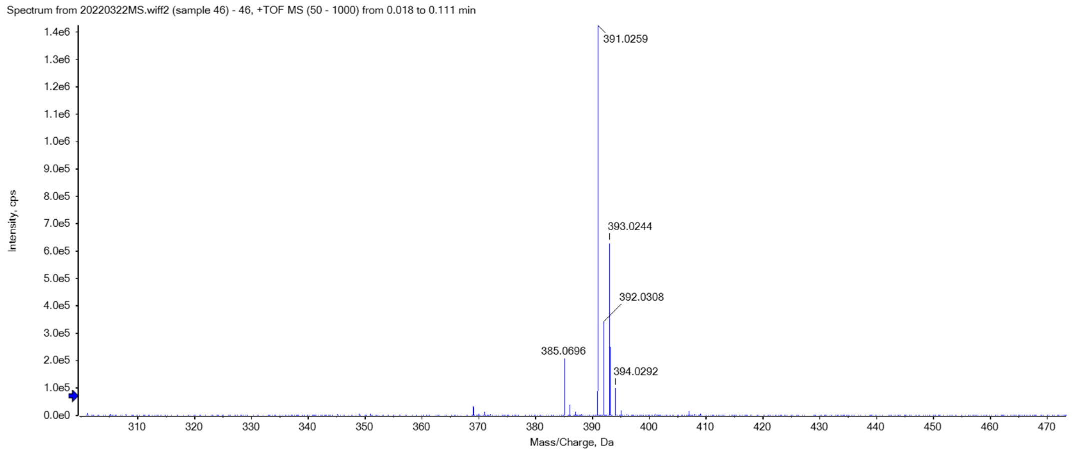

Figure S99. HRMS spectrum of compound **7f**.

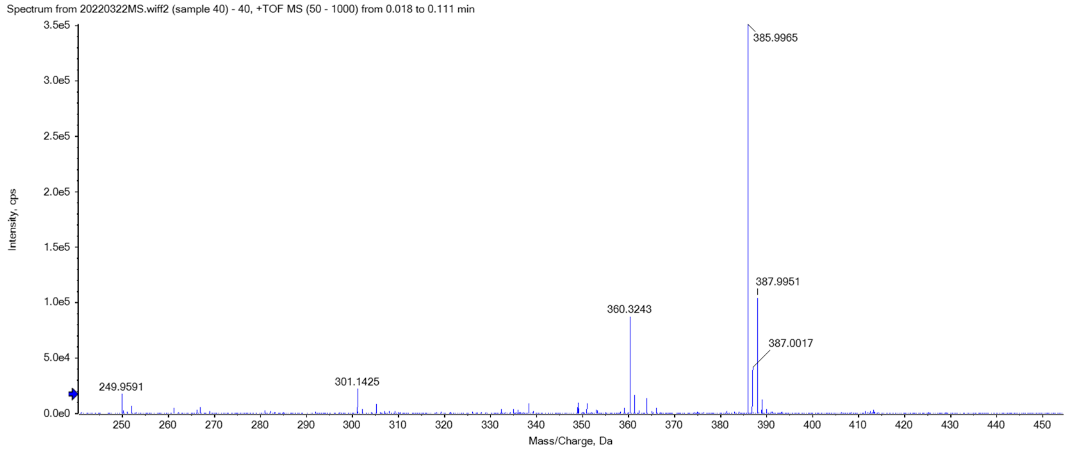

Figure S100. HRMS spectrum of compound **7g**.

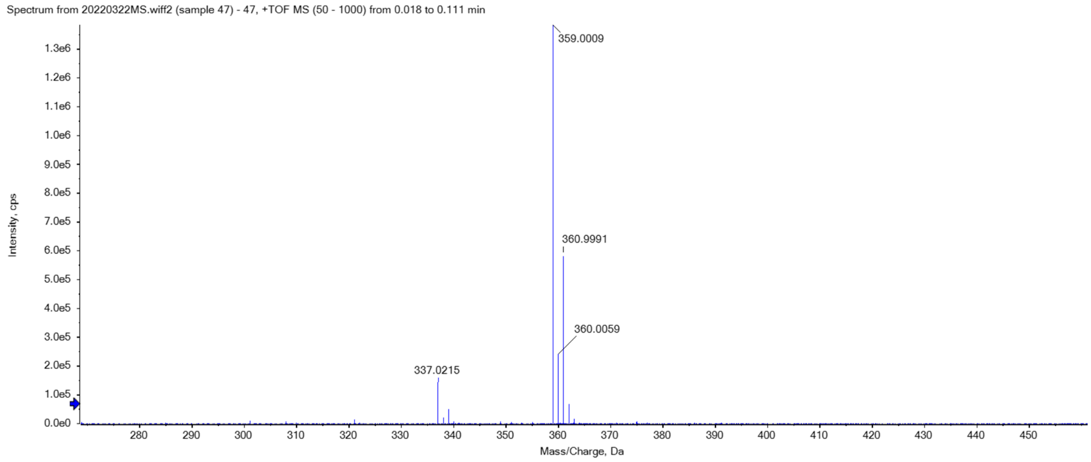

Figure S101. HRMS spectrum of compound **7h**.

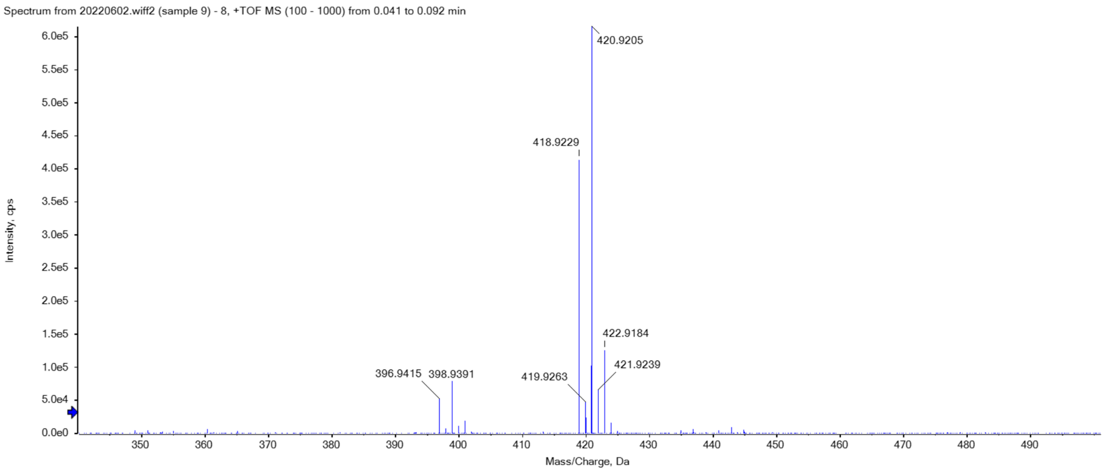

Figure S102. HRMS spectrum of compound **7i**.

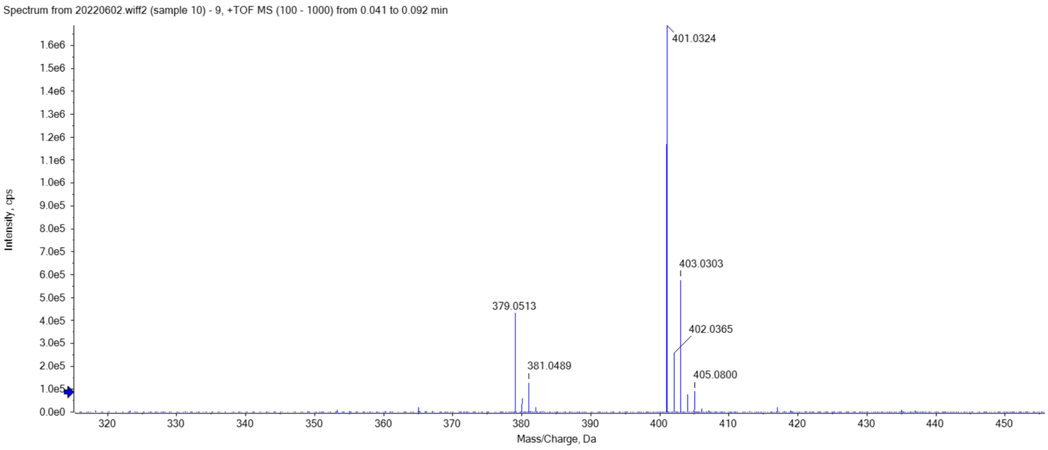

Figure S103. HRMS spectrum of compound **7m**.

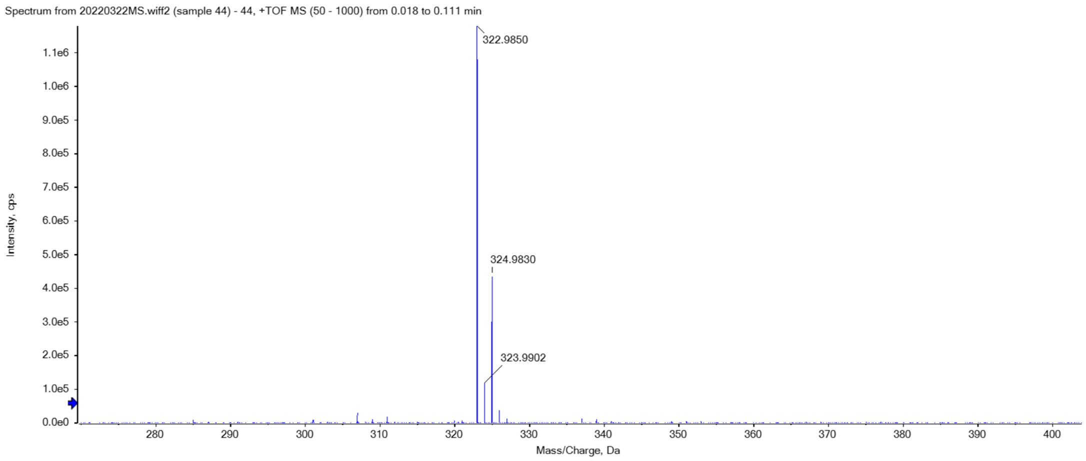

Figure S104. HRMS spectrum of compound **7n**.

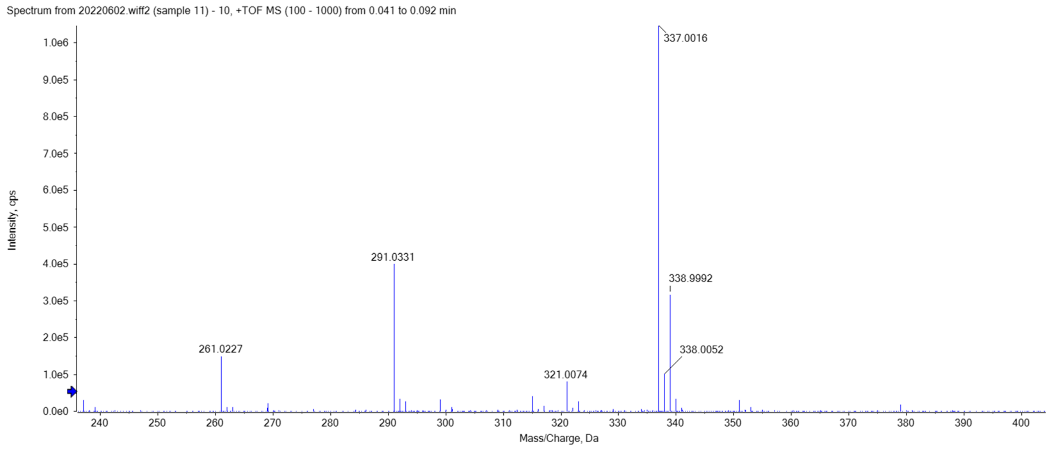

Figure S105. HRMS spectrum of compound **7o**.

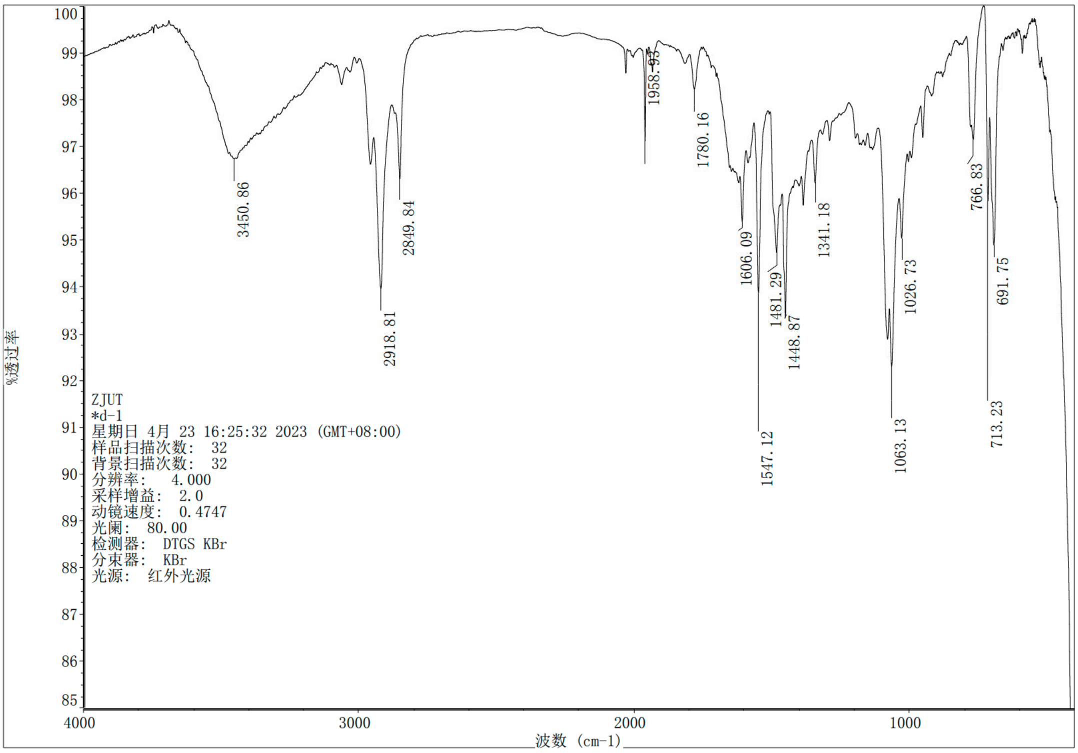

IR (KBr): 3450, 2918, 2849, 1606, 1547, 1448, 1063, 713 cm^{-1}

Figure S106. IR (KBr) spectrum of compound **5a**.

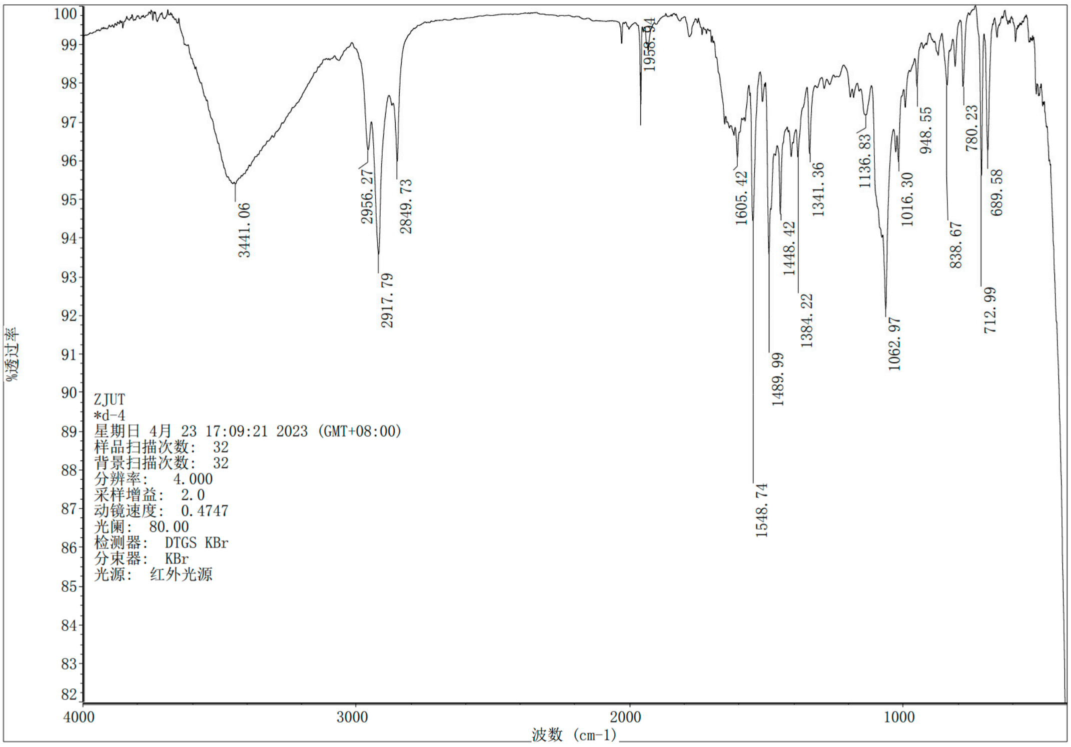

IR (KBr): 3441, 2917, 1548, 1489, 1062, 712, 689 cm^{-1}

Figure S107. IR (KBr) spectrum of compound **5b**.

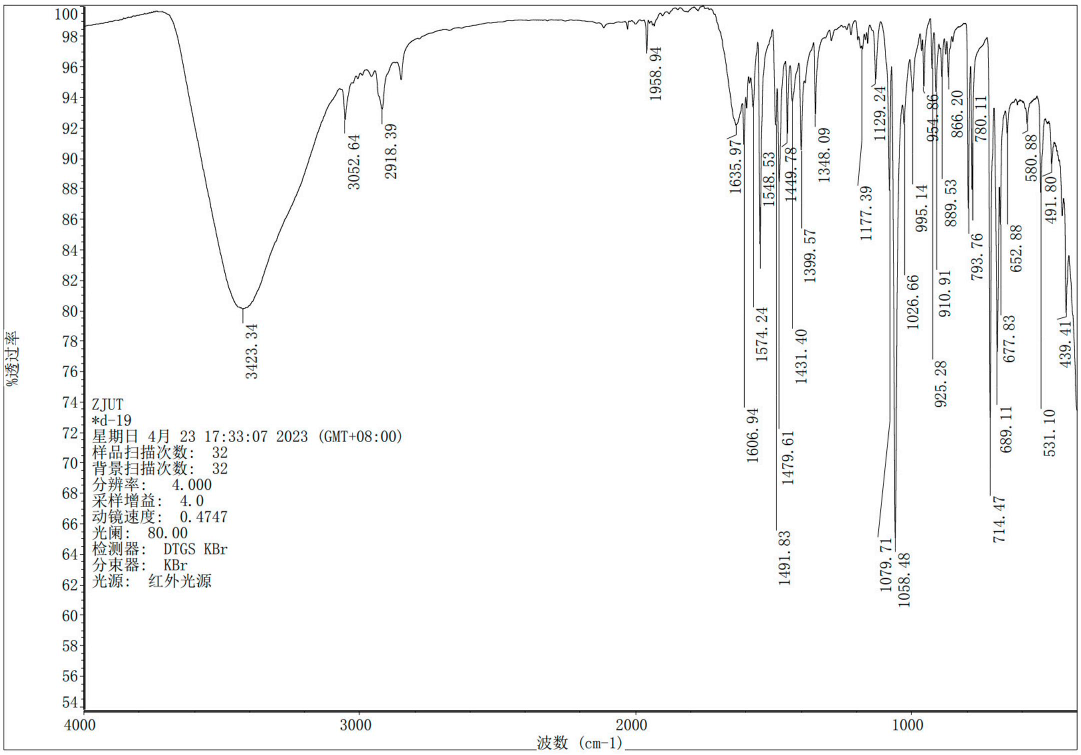

IR (KBr): 3423, 3052, 2918, 1548, 1491, 1079, 1058, 714, 689, 677 cm^{-1}

Figure S108. IR (KBr) spectrum of compound **5c**.

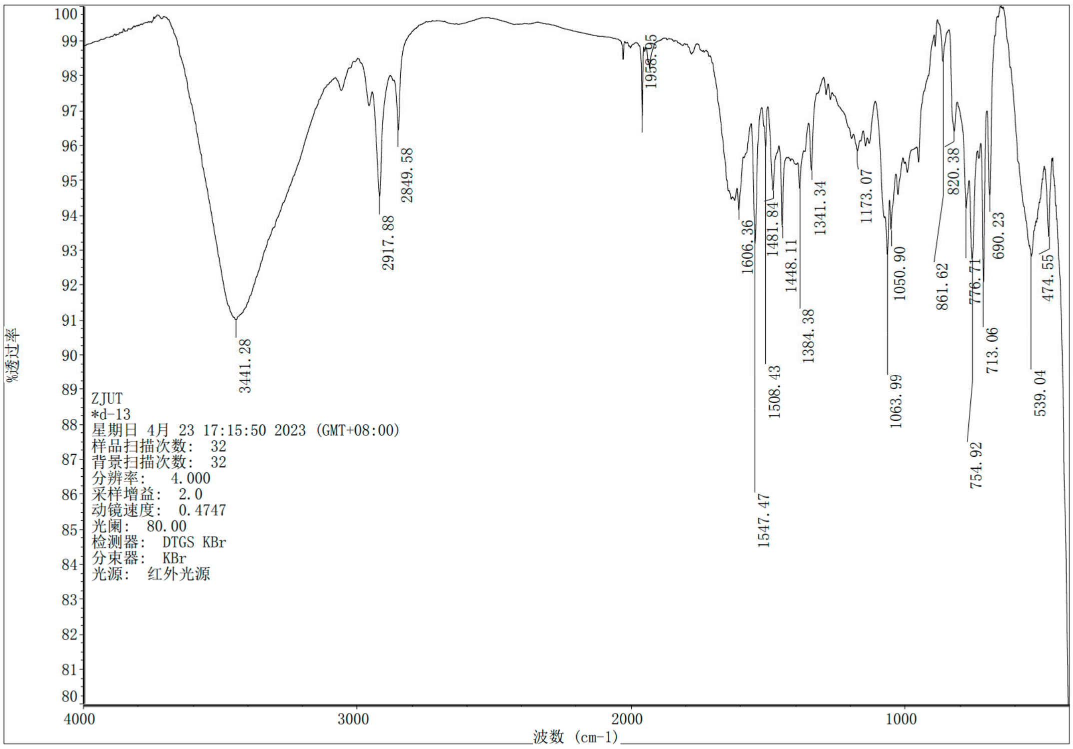

IR (KBr): 3441, 2917, 2849, 1547, 1508, 1063, 754, 713 cm^{-1}

Figure S109. IR (KBr) spectrum of compound **5f**.

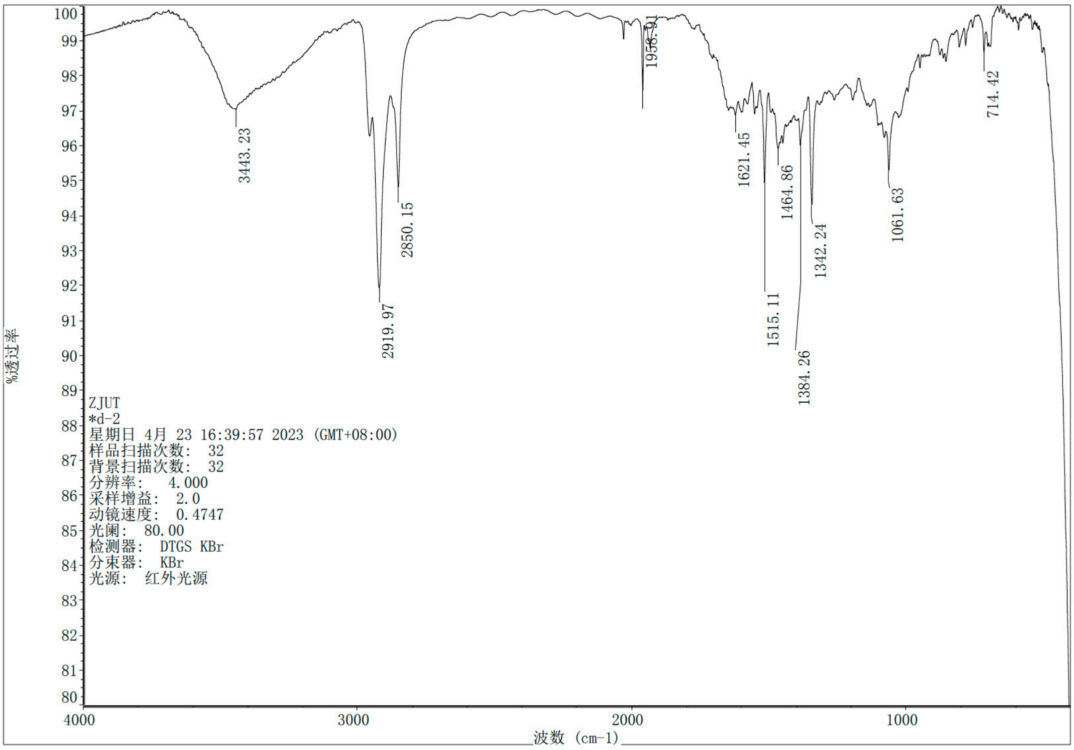

IR (KBr): 3443, 2919, 2850, 1515, 1384, 1342, 1061, 714 cm^{-1}

Figure S110. IR (KBr) spectrum of compound **5g**.

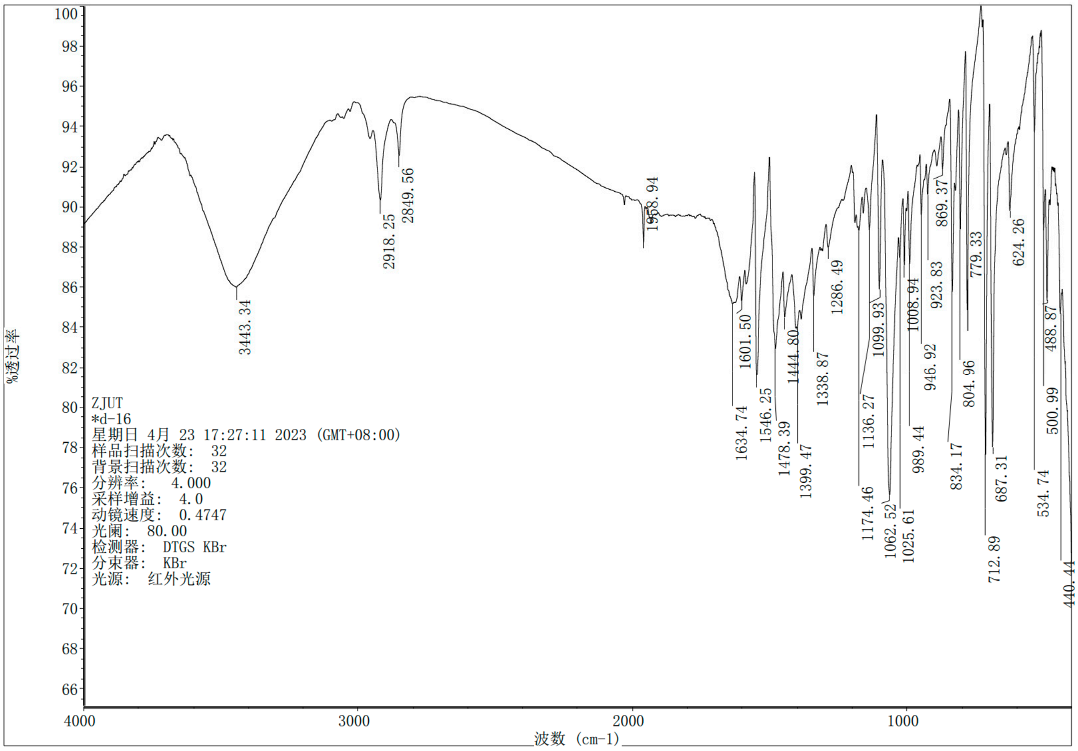

IR (KBr): 3443, 2918, 2849, 1546, 1478, 1499, 1174, 1062, 1025, 712, 687 cm^{-1}

Figure S111. IR (KBr) spectrum of compound **5i**.

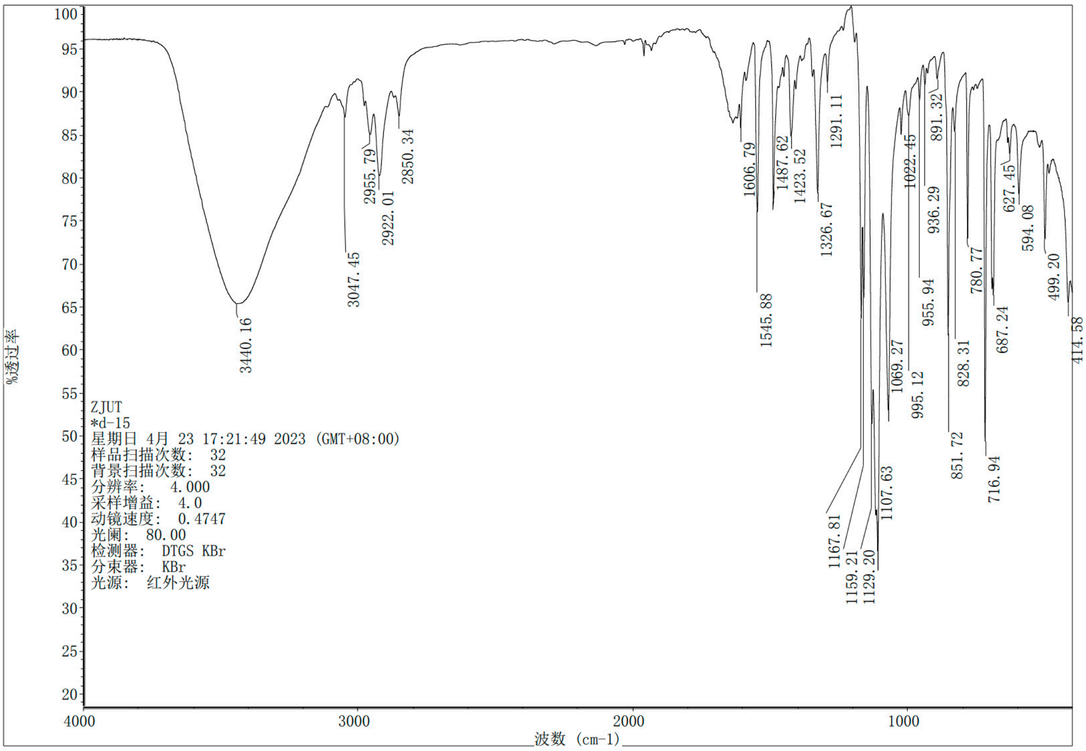

IR (KBr): 3440, 3047, 2955, 2922, 2850, 1545, 1326, 1167, 1159, 1129, 1107, 851, 716 cm^{-1}

Figure S112. IR (KBr) spectrum of compound **5j**.

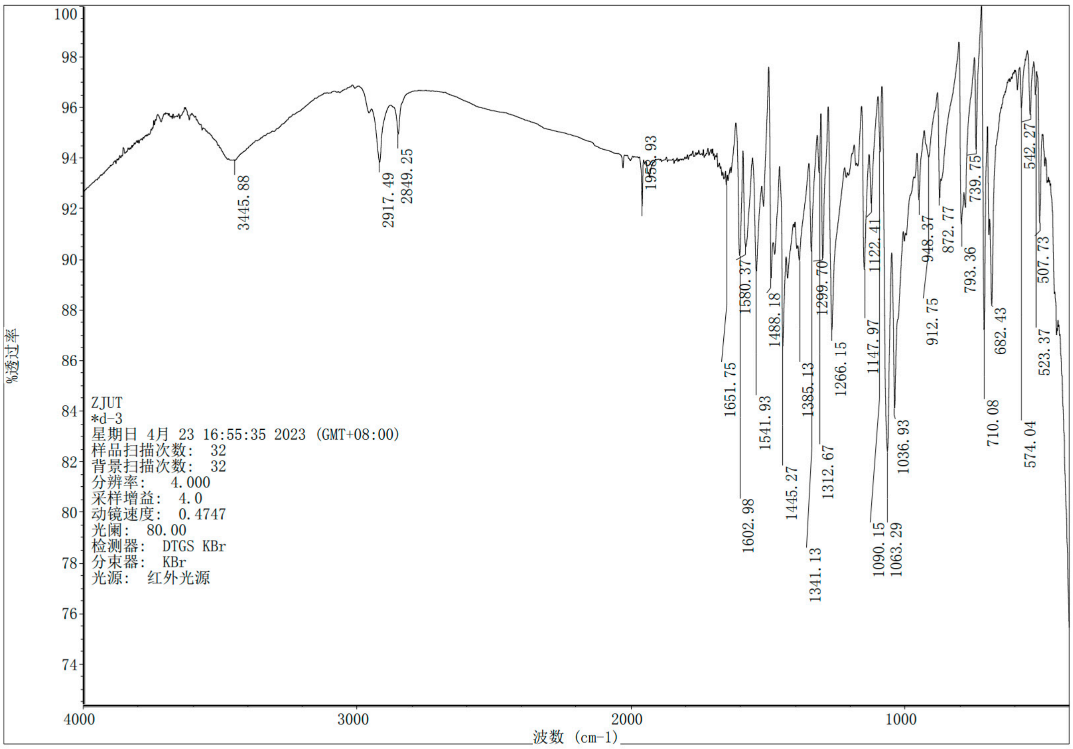

IR (KBr): 3445, 2917, 2849, 1602, 1541, 1445, 1341, 1063, 1036, 710, 682 cm^{-1}

Figure S113. IR (KBr) spectrum of compound **5k**.
